# Multifaceted catalytic hydrogenation of amides via diverse activation of a sterically confined bipyridine–ruthenium framework

**DOI:** 10.1038/s41598-017-01645-z

**Published:** 2017-05-16

**Authors:** Takashi Miura, Masayuki Naruto, Katsuaki Toda, Taiki Shimomura, Susumu Saito

**Affiliations:** Graduate School of Science, Nagoya University, Chikusa, Nagoya 464-8602 Japan

## Abstract

Amides are ubiquitous and abundant in nature and our society, but are very stable and reluctant to salt-free, catalytic chemical transformations. Through the activation of a “sterically confined bipyridine–ruthenium (Ru) framework (molecularly well-designed site to confine adsorbed H_2_ in)” of a precatalyst, catalytic hydrogenation of formamides through polyamide is achieved under a wide range of reaction conditions. Both C=O bond and C–N bond cleavage of a lactam became also possible using a single precatalyst. That is, catalyst diversity is induced by activation and stepwise multiple hydrogenation of a single precatalyst when the conditions are varied. The versatile catalysts have different structures and different resting states for multifaceted amide hydrogenation, but the common structure produced upon reaction with H_2_, which catalyzes hydrogenation, seems to be “H–Ru–N–H.”

## Introduction

As a result of high thermodynamic stability and kinetic inertness^1–3^, amides have been found in natural systems for millennia, as the repeating units of functional polypeptides (proteins), and have more recently become a valuable commodity as the monomer units (e.g., α, β-unsaturated carboxamides, caprolactams) of synthetic polymers including poly(acrylamide), nylons, and Kevlar produced on an enormous scale. Rapidly emerging C(sp^3^)–H and C(sp^2^)–H bond activation strategies that lead to C–N^4, 5^ and C–C^6, 7^ bond formation frequently utilize amides as directing groups. Were it possible to develop an effective method for the catalytic hydrogenation of amides (formally, a hydrogenolysis–hydrogen addition sequence) that leads to selective C–N bond cleavage in preference to C=O bond cleavage, alcohols and amines would be generated. Both are useful platform chemicals or intermediate building blocks for organic synthesis. Alcohol and amine monomer units could also be regenerated/recycled from waste polyamides, which would otherwise be disposed of via combustion, resulting in the emission of CO_2_ and NO_*x*_. For example, the hydrogenation leading to effective C–N bond cleavage in *N*, *N*-dimethyl formamide (DMF) produces CH_3_OH. This method, if it can be developed, would be potentially useful for enhancing the “anthropogenic chemical carbon cycle (methanol economy),” as suggested by Olah^8^, in conjunction with the elegant DMF synthesis reported by Noyori^9^ involving the reaction of supercritical CO_2_ with H_2_ and Me_2_NH, catalyzed by a ruthenium (Ru) complex with an extremely high turnover number (TON) (substrate/catalyst ratio: S/C = 370,000). A tandem stoichiometric amine- [(CH_3_)_2_NH-^10^ or 2-aminoethanol-^11^] and catalytic ruthenium (Ru)-promoted hydrogenation of CO_2_ to CH_3_OH was also reported.

Amide hydrogenation systems involving molecular catalysts developed separately thus far show different selectivity and reactivity, and are complementary to each other. Cole-Hamilton^12, 13^, Leitner/Klankermayer^13–15^, and Beller^16^ have developed different (triphos)Ru catalyst systems (triphos = CH_3_C[CH_2_PPh_2_]_3_; Ph = C_6_H_5_) for selective cleavage of the C=O bond of amides. When combined with catalytic Yb(OSO_2_CF_3_)_3_, hydrogen pressure (*P*_H2_) can be reduced as low as ca. 0.5 MPa^16^. Use of a combination of (*P*, *C*, *P*)Ir pincer complex and stoichiometric B(C_6_H_5_)_3_ was also proved to be effective for inducing the C=O bond cleavage^17^. In contrast, Ru complexes for the selective cleavage of the C–N bond of amides were intensively studied by Ikariya ((*P*, *N*H)Ru)^18–21^, Milstein ([*P*,(*N*, *N*)_bpy_]Ru, where bpy = bipyridine; (*N*, *N*)_bpy_ = bipyridine nitrogens)^22–25^, Bergens ([(*P*, *N*H_2_)(*P*, *N*H_2_)]Ru)^26–28^, Leitner/Klankermayer, ((triphos)Ru)^15^, and Beller ((*P*,*N*H, *N*)Ru)^29^. Milstein’s milestone discovery, in which deprotonation at the 2-(pyridyl)methylene unit in the (*P*, *N*_py_, P)Ru pincer complexes induces an active catalyst^30^, has had a great impact on the ensuing molecular design of hydrogenation catalysts (py = pyridine; *N*_py_ = pyridine nitrogen). Iron (Fe) complexes catalyze the C–N bond cleavage^31, 32^ with the aid of the tridentate (*P*, *N*H, *P*) ligand originally developed by Takasago Co. for ester hydrogenation^33, 34^. A (*P*, *N*_py_, *P*)Fe pincer complex showed comparable catalytic activity^35^. Unfortunately, however, those metal complexes hydrogenate majorly a range of strongly or moderately activated amides, including *N*-aryl-, *N*-acyl-, *N*-(di)methyl-, and α-alkoxy amides and morpholino ketones, as well as relatively simple and small amides (e.g., formamides, acetamides, trifluoroacetamides). Very recently, Zhang^36^ reported an extremely active (*P*, *N*_py_, *N*H, *P*)Ru catalyst that hydrogenates, in most cases, activated amides, where a rather low hydrogenation temperature (100 °C) was only tested. Mashima^37^ reported the use of a combination of a Ru complex (2 mol %) bearing two bidentate (*P*, *N*H_2_) ligands, KO*t*Bu (20 mol %) and Zn salts (4 mol %), which promoted the hydrogenation of *N*-methyl amides at 100 °C, but the reactivity of sterically more demanding amides and primary amides was scant to moderate; the catalysis does not seem to be sustainable at elevated temperatures.

Thus, a possibility of a new *robust* and versatile catalyst system for amide hydrogenation that tolerates not only mild but also harsh (high reaction temperature (*T*) and high hydrogen pressure (*P*_H2_)) reaction conditions has remained elusive. An exploration of hydrogenation precatalyst, generally and potentially be applicable for both C–N and C=O bond cleavage, as well as for different classes of amides such as those found in DMF, oligopeptides and artificial polyamides, remains a grand challenge; however, there is a lack of basic knowledge concerning the molecular design of a structurally robust, multifunctional catalysts derived diversely from a single precatalyst competent under various reaction conditions necessary to achieve such multifaceted amide hydrogenation.

**Figure 1:**
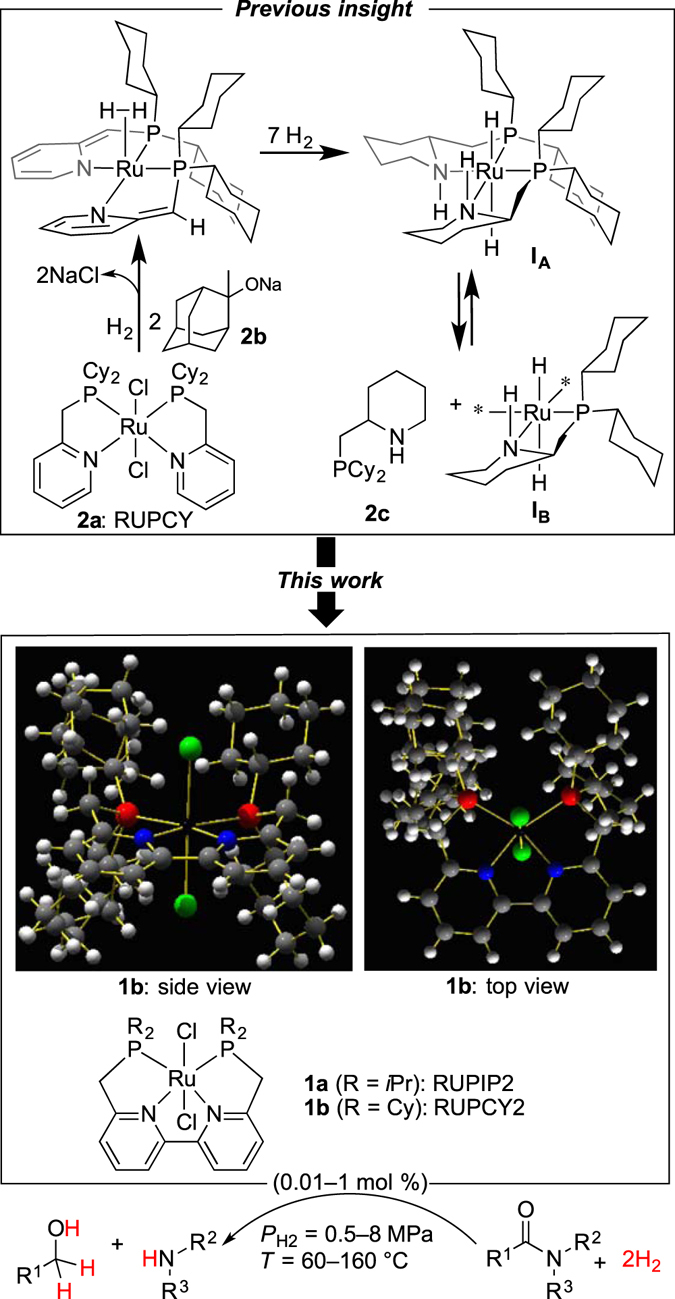
Summary of this work. Our earlier work (in the upper square) illuminated possible catalysts **I**_**A**_ and **I**_**B**_ derived from **2a**, which is modified to new Ru complexes **1a** and **1b** (in the lower square); and the X-ray single crystal structure of **1b**. Ru (black), Cl (green), N (blue), P (red), C (grey), H (white).

We recently reported preliminary research on the molecular design of [(*P*, *N*_py_) (*P*, *N*_py_)]Ru complexes including RUPCY (**2a**, Cy = C_6_H_11_)^38–41^, which was shown to be effective for the hydrogenation of unactivated amides^38, 40, 41^. Unfortunately, however, the catalyst system is only viable under harsh reaction conditions (S/C = 50; *P*_H2_ = 4–8 MPa; *T* = 140–180 °C, reaction time (*t*) = 24–216 h). Herein is reported a greatly improved method for the hydrogenation of unactivated amides (Fig. 1), realized by (*P*,(*N*, *N*)_bpy_, *P*)Ru complexes RUPIP2 (**1a**, *i*Pr = (CH_3_)_2_CH) and RUPCY2 (**1b**) as precatalysts, from which versatile catalysts are generated by incorporation of multiple hydrogen atoms to a sterically confined bpy–Ru framework (molecularly well-designed site to confine adsorbed H_2_ in). Through simple but rational structural modification of **2a** to **1a** ^41^ and **1b** ^41^, catalyst performance for C–N bond and C=O bond cleavage has been significantly advanced (TON up to 7700) under both mild (*P*_H2_ = 0.5–2 MPa; *T* = 60–120 °C) and harsh (*P*_H2_ = 3–8 MPa; *T* = 130–160 °C) conditions.

## Results and Discussion

In our previous study^38^, it was shown that a plausible catalyst, either **I**_**A**_ or **I**_**B**_, was likely induced upon treatment of precatalyst **2a** with bulky base **2b** and H_2_ (Fig. 1). Under the preactivation conditions for **2a**, full hydrogenation of the pyridines of **2a** gave a new bidentate ligand **2c** incorporating the piperidine (N–H) unit. It is therefore likely that hydrogen(s) transfer takes place from the four-centered “H–Ru–N–H” unit to an amide carbonyl group (Noyori’s bifunctional mechanism)^42–44^, affording a *N*, *O*-hemiacetal R^1^CH(OH)(NR^2^R^3^). Thus, this amide hydrogenation mechanism may involve an outer-sphere, rather than inner-sphere, mechanism that involves a two-step pathway, wherein the Ru catalyst having an H–Ru–N–H functionality is generated in the first step, followed by the amide carbonyl group interacting with both of the H of the H–Ru–N–H component to facilitate the hydrogen (H^−^ and H^+^) transfer. However, the question as to which catalyst (**I**_**A**_ or **I**_**B**_) is more active for hydrogenation has yet to be answered. To probe this, Ru complex **1b** bearing a tetradentate bpy analog ligand was synthesized, in which the two bidentate ligands of **2a** are connected. This simple ligand manipulation rules out the possibility of facile detachment of the ligand from the Ru center, which the bidentate ligand **2c** of **I**_**A**_ and **I**_**B**_ underwent. According to the X-ray single crystal structure analysis of **1b** (Fig. 1, ball-and-stick models; additional structural parameters are available in Supplementary Fig. 11), there are fifteen (non-H) atoms (the bpy, Ru, and two phosphorus atoms) in a planar orientation constituting a “sterically confined bpy–Ru framework” with four sterically bulky cyclohexyl (Cy) architectures on top and bottom. The sterically encumbered H_2_-adsorption site could preferentially confine the small molecule in, rather than more sterically bulky substrates such as amides. Steric repulsion between an incoming substrate and the four Cy groups reasonably suppresses substrate–Ru interactions that may cause catalyst inhibition and/or decomposition of catalysts.

**Figure 2:**
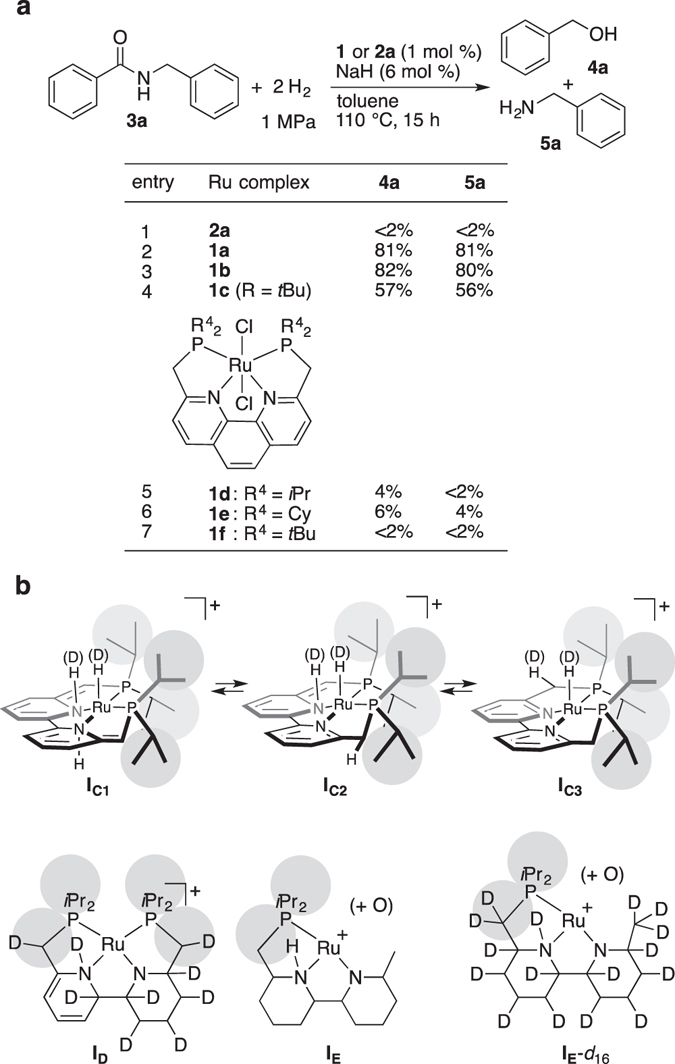
Precatalysts tested and diverse catalysts derived from **1a**. (**a**) Hydrogenation of **3a** using different Ru-complexes **1a**–**f** and **2a** ([Ru]_0_ = 3.3 mM) as precatalysts. (**b**) Major resting states of catalysts. Calculated exact masses: **I**_**C1**_–**I**_**C3**_ (519.1626), **I**_**C**_-*d*_2_ (521.1752), **I**_**D**_ (534.2661), **I**_**E**_ (429.1603) and **I**_**E**_-*d*_16_ (445.2608).

At the outset, the performance of **1b** as catalyst was compared with that of **2a** (Fig. 2a): toluene solutions of each Ru complex (1 mol %), amide **3a** (100 mol %), and NaH (6 mol %) were reacted under identical reaction conditions ([Ru]_0_ = 3.3 mM, S/C = 100; *P*_H2_ = 1 MPa, *T* = 110 °C, *t* = 15 h). The apparent reaction rate obtained using **1b** (**4a**: 82%) was more than 40-fold faster than that with **2a** (**4a**: <2%) under mild conditions (entry 1 vs. 3). By replacing **1b** with **1a**, the initial load of the Ru complex can be reduced to not less than 0.25 mol % (S/C = 400; NaH: 5 mol %), giving **4a** and **5a** in 73% and 74% yield, respectively (Supplementary Table 2, entry 2; a TON of ca. 300, calculated as product (mol)/**1a** (mol)) ([**1a**]_0_ = 0.83 mM, *P*_H2_ = 1 MPa, *T* = 110 °C, *t* = 24 h). The catalytic activity of **1a** and **1b** exceed the best reported values obtained under similar conditions by a wide margin (**4a**: 57%: S/C = 100, *P*_H2_ = 1 MPa, *T* = 110 °C, *t* = 48 h, TON = 57)^22^. From this, the turnover frequency (TOF = TON **·** h^−1^) can be roughly estimated to be not more than 10-fold greater in this new system (300/24 vs. 57/48). In the meantime, remarkably high TON of 8800 was observed in the hydrogenation of **3a** catalyzed by a (*P*, *N*_py_, *N*H, *P*)Ru complex, albeit with much higher *P*_H2_ (*P*_H2_ = ca. 5 MPa, *t* = 20 h)^36^.

In order to find a more competent catalyst, several derivatives of **1** (**1c** ^41, 45^, **1d** ^41^, **1e** ^41^, and **1f** ^46^) were synthesized as different precatalysts and tested in the hydrogenation of **3a** under similar conditions ([**1**]_0_ = 3.3 mM, S/C = 100; **1**:NaH:**3a** (mol %) = 1:6:100; *P*_H2_ = 1 MPa, *T* = 110 °C, *t* = 15 h) (Fig. 2a)^41^. The phenanthroline series **1d**–**f**, which has a more extended π-conjugation system as H_2_-adsorbent, was totally ineffective (entries 5–7). The sterically more bulky **1c** (R = *t*Bu) also showed less promising results (entry 4). In order to minimize steric repulsion between the catalyst and the incoming substrate, including less reactive, sterically bulky amides, **1a** was chosen for further investigation.

Bulky base **2b** (Fig. 1), which was used previously^38^ as an additive for the preactivation of **2a** in the absence of amide, was as effective as NaH for the amide hydrogenation catalyzed by **1a** and **1b**. Hydrogenation of three different, rather activated amides was also tested using **1a** (0.01 or 0.1 mol %) preactivated with **2b** or NaH (0.1 or 1 mol %) under H_2_ pressure (*P*_H2(pre)_) = 1 or 8 MPa and temperature (*T*_pre_) = 160 °C, and the results were compared with those reported by Bergens^27^, in which [Ru(Ph_2_P(CH_2_)_2_NH_2_)_2_(η^3^-allyl)]^+^(BF_4_^−^) was used with a larger amount of a base (4 mol % of KN[Si(CH_3_)_3_]_2_ or 5 mol % of NaOCH_3_) (Supplementary Table 1 for reaction conditions and comparison of hydrogenation results). Higher to comparable TON and TOF under identical hydrogenation conditions (*P*_H2_ = 5 MPa, *T* = 100 °C, *t* = 24 h) were consistently shown by **1a** in the hydrogenation of *N*, *N*-dimethylacetamide (TON = 530 vs. 500), *N*-phenylacetamide (TON = ~800 vs. 700), and *N*-phenyl-2-pyrrolidone (TON = 7700 vs. 7120) under more neutral p*H*. It turned out, however, that this preactivation step in the absence of amide could be eliminated using NaH. The resting state of a matured catalyst generated upon activation of **1a** in the presence or in the absence of amide **3a** ([**1a**]_0_ = 3.3 mM in toluene; **1a**:NaH (mol %) = 1:10; [**3a**]_0_ = 5.0 mM or 0; *P*_H2(pre)_ = 1 MPa, *T*_pre_ = 110 °C, *t*_pre_ = 5 h) was thus evaluated. Solutions of catalyst (**1a** activated with and without **3a**) were prepared separately, and the samples were measured directly via electrospray ionization-mass spectroscopy (ESI-MS) (Supplementary Figs 1 and 2). Both samples showed a Ru species that retained a tetradentate ligand-incorporated structure during the induction period of the catalyst: organic frameworks comprised of a Ru with slightly different molecular weights (*m*/*z* = 519.1626 ± 0.0052 (strong intensity), 565.1317 ± 0.0021 (**I**_**C**_ − 2 H + 3 O: small to moderate intensity)) were consistently detected. Both signals correspond to structures less the two Cl groups from the original **1a**. The former intense signal corresponds to **I**_**C1**_–**I**_**C3**_ (^1^H NMR (ppm): δ −8.62 (t (dd), *J* = 29.7 Hz, RuH)), produced by deprotonation of two methylene groups (CH_2_–bpy–CH_2_) of **1a** (with two NaCl formation), followed by H_2_ adsorption (Fig. 2b). The deprotonation^30, 38^ and subsequent capture of one H_2_ molecule was confirmed using D_2_ instead of H_2_ (Supplementary Fig. 3). The ^1^H NMR signal at δ −8.62 became significantly weaker, and the ESI-MS signal of **I**_**C1**_–**I**_**C3**_ disappeared; instead, a signal at 521.1752 ± 0.0070 (**I**_**C**_ − H_2_ + D_2_) was observed as the only intense signal. In contrast, when activation of **1a** was carried out under the identical conditions, except for *P*_H2(pre)_ = 8 MPa and *T*_pre_ = 160 °C or *P*_H2(pre)_ = 4 MPa and *T*_pre_ = 140 °C being used, the base peak (*m*/*z* = 429.1603 ± 0.0016) was consistent with **I**_**E**_ (or its tautomers): fully hydrogenated bipyridine with concurrent cleavage of one C–P bond (Supplementary Figs 4–6)^47, 48^. The corresponding **I**_**E**_-*d*_16_ (*m*/*z* = 445.2608 ± 0.0090) was also detected using D_2_ instead of H_2_, suggesting hydrogenolysis of the C–P bond. Under milder conditions (*P*_D2(pre)_ = 1 MPa, *T*_pre_ = 160 °C), **I**_**D**_ (*m*/*z* = 534.2661 ± 0.0019 or its tautomers) was also detected as a major product (Supplementary Figs 7 and 8) which would correspond to an intermediate during the structural changes from **I**_**C**_ to **I**_**E**_, during which time deuterium atoms from multiple D_2_ (one to eight molecules) are in turn incorporated. The oxygenation of **I**_**E**_ likely occurred during aerobic sample injection into the ESI-MS instrument^38^, so that any oxygen atom-incorporating processes affecting the hydrogenation steps could be fully ruled out. These experiments show that different catalyst resting states, in which the common structure produced upon reaction with H_2_ is “H–Ru–N–H” (Fig. 2b), are generated to catalyze hydrogen transfer. The hydride- and proton-transfer to an amide carbonyl most likely occurs from a “H(δ^−^)–Ru–N–H(δ^+^)” fragment, in which both the Ru–H and N–H face the same direction. To summarize, boundary conditions as to whether multiple hydrogen addition would occur to the H_2_ adsorption site of **1a** depend on both *P*_H2(D2)(pre)_ and *T*_pre_. When a lower *P*_H2(D2)(pre)_ = 1 MPa was used, intermediate **I**_**C**_ was the major species with *T*_pre_ = 110 °C, while **I**_**C**_ was multiply in turn hydrogenated (*T*_pre_ < 160 °C), giving **I**_**E**_(-*d*_16_) as a minor species through the formation of partially hydrogenated **I**_**D**_. On the other hands, with *P*_H2(pre)_ = 4–8 MPa and *T*_pre_ = 140–160 °C, **I**_**C**_ was converted more rapidly into **I**_**E**_ as a major or exclusive species. The diverse mechanism hidden behind the activation process of **1a** would be entirely different from that in the ester hydrogenation poorly promoted by **1f** ^46^ and effectively catalyzed with its relevant Ru complexes bearing tetradentate (*N*,(*N*, *N*)_bpy_, *P*) ligands (*P*_H2_ = 5–10 MPa, *T* = 25–100 °C) reported by Zhou^45^, in which a possibility of multiple hydrogen additions to precatalyst was not totally investigated under milder *T*. The scant catalytic activity derived from phenanthroline series **1d**–**f** could partly be explained by a more acidic nature of the NH group of aniline structures in the catalyst induced by activation of **1d**–**f** (Supplementary Figs 9 and 10; Supplementary Table 2, entry 3). In any event, the H_2_-adsorption site with bulky *i*Pr (or Cy) groups is non-innocent and undergoes meaningful structural changes via multiple hydrogen additions before producing the diverse, active forms of catalyst. These results further suggest that a Ru complex bearing a (*P*,(*N*, *N*)_bpy_, *P*) ligand is more versatile and robust in the production of diverse catalysts than Ru precatalysts bearing simple bidentate (*P*, *N*_py_)^38, 40^ and (*P*, *N*H)^18–21, 26–28, 37^, tridentate (*P*,(*N*, *N*)_bpy_)^22–25^, and tetradentate (*P*, *N*_py_, *N*H, *P*)^36^ ligand(s).

**Figure 3:**
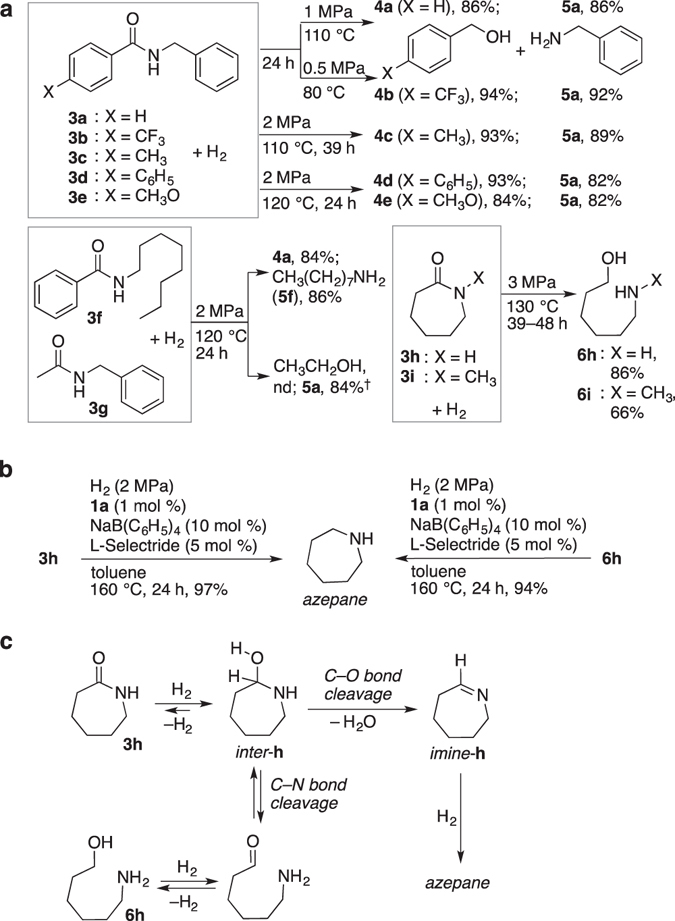
Hydrogenation of aromatic and cyclic amides. Unless otherwise specified, the reaction was performed using **1a** (1 mol %), **3** (100 mol%), and NaH (6–10 mol %) in toluene. Pressure indicated is of H_2_ at 25 °C. nd: not determined. See Supplementary Information for experimental details. (**a**) Hydrogenation with *P*_H2_ = 0.5–3 MPa, *T* = 80–130 °C. (**b**) Additive effects on selective C=O bond cleavage in the hydrogenation of a cyclic amide and in the dehydrogenation/hydrogenation of a linear amino alcohol. (**c**) Possible reaction pathways starting from **3h** and **6h** under hydrogenation conditions. ^†^ **1a** was preactivated: *P*_H2(pre)_ = 1 MPa, *T*_pre_ = 160 °C, *t*_pre_ = 5 h.

**Figure 4:**
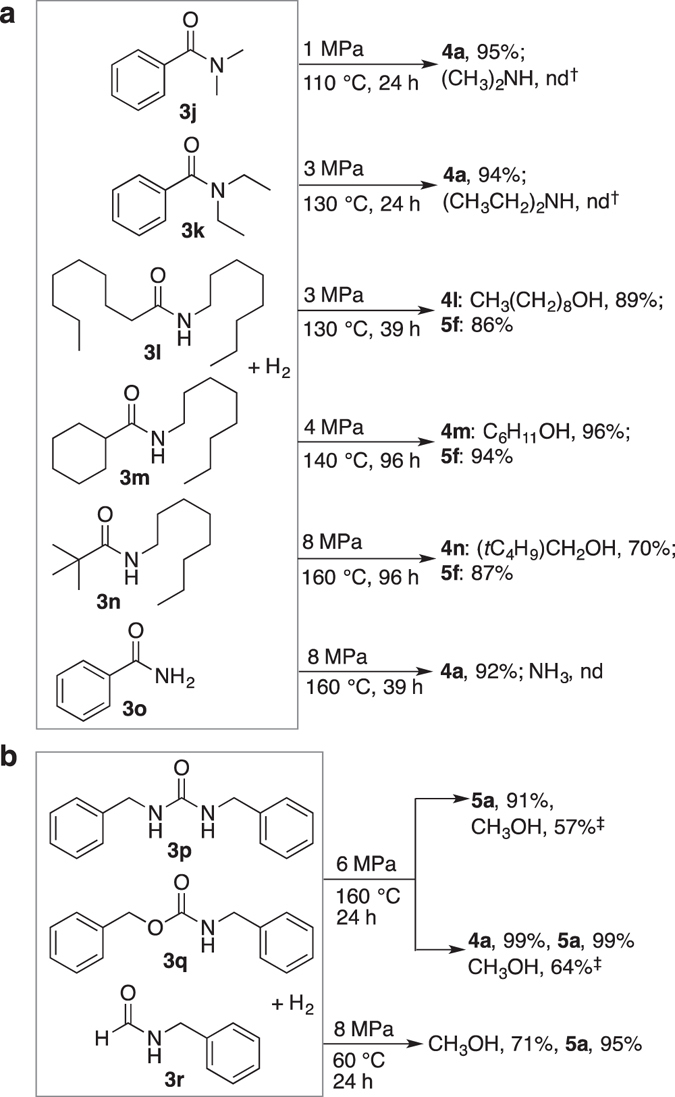
Hydrogenation of various unactivated amides. Unless otherwise specified, the reaction was performed using **1a** (1 mol %), **3** (100 mol%), and NaH (6–10 mol %) in toluene. Pressure indicated is of H_2_ at 25 °C. nd: not determined. See Supplementary Information for experimental details. (**a**) Comparison of effects of steric bulkiness of **3** on reaction conditions. ^†^ **1a** was preactivated: *P*_H2(pre)_ = 1 MPa, *T*_pre_ = 160 °C, *t*_pre_ = 5 h. (**b**) Hydrogenation of CO_2_-derived **3**. ^‡^1 mol % **1b** preactivated was used instead of **1a**: *P*_H2(pre)_ = 8 MPa, *T*_pre_ = 160 °C, *t*_pre_ = 2 h.

Hydrogenation can be started conveniently by mixing air-stable **1a**, amide **3**, and NaH together, followed by pressurizing the reaction vessel with H_2_ and then elevating *T*. Catalyst preactivation procedures in a separate reaction vessel is not necessarily needed. The hydrogenation of various unactivated amides under different conditions was tested, and the results are given in Figs 3 and 4 (Supplementary Table 2 gives detailed reaction conditions). Primary, secondary, and tertiary amides showed excellent compatibility with the same precatalyst, regardless of steric demands or whether aromatic/aliphatic. In order to shorten the reaction time for practical application, it is better to slightly increase the *T* to 120–130 °C, and *P*_H2_ to 2–3 MPa (Fig. 3a). The hydrogenation of ε-caprolactam (**3h**), a cyclic amide which serves as the monomer of nylon-6, showed a similar pattern of C–N bond cleavage, giving amino alcohol **6h** predominantly (azepane: 1%). For the hydrogenation of unactivated amide **3h**, the catalytic activity of **1a** (0.1 mol %) preactivated with a less amount of **2b** was far superior to that of the Bergens’s catalyst^27^ (TON = 970 with 1 mol % of **2b** vs. 230 with 5 mol % of KN[Si(CH_3_)_3_]_2_ under the identical conditions (*P*_H2_ = 5 MPa, *T* = 100 °C, *t* = 24 h), giving **6h** in 97% yield (Supplementary Table 1 for details). Products **6h** and **6i** are synthetic precursors of *N*, *N*-dimethyl-6-amino-1-hexanol, a polymerization initiator in polyurethane synthesis^49^. In marked contrast, when co-additives, L-Selectride (lithium tri-*sec*-butylborohydride) and NaB(C_6_H_5_)_4_, were used instead of NaH, the selective C–N bond cleavage was switched entirely to C=O bond cleavage, giving azepane in a quantitative yield (Fig. 3b). The hydrogenation of **3h** to azepane proceeded under much milder conditions (*P*_H2_ = 2 MPa), compared with a very recent example in which catalytic (triphos)Ru derivatives were used (*P*_H2_ = ca. 10 MPa)^14^. Although **3h** and *inter*-**h** might be in an equilibrium even under H_2_^40^, the net reaction would proceed via either one of (or both of) the following two pathways involving the same reaction intermediate, *N*, *O*-hemiacetal *inter*-**h**, which undergoes (Fig. 3c): (1) direct cleavage of the C–O bond and subsequent hydrogenation of the resulting *imine*-**h**; or (2) the C–N bond cleavage that leads to the equilibrium (hydrogenation of the aldehyde ⇆ dehydrogenation of the resulting CH–OH group ⇆ intramolecular cyclization to *inter*-**h**), followed by the reaction steps shown in (1). Indeed, when aminoalcohol **6h** was used instead of **3h**, the identical hydrogenation conditions gave azepane almost quantitatively.

Hydrogenation of the more sterically demanding amides **3k**, **3m**, and **3n** also took place, capitalizing on the structural robustness (negligible ligand detachment) of the catalyst even under harsher reaction conditions (*P*_H2_ = 3–8 MPa, *T* = 130–160 °C) (Fig. 4a). The mercury test^38^ was also employed, in which Hg(0) (150 mol %) was added during the hydrogenation of **3n** to probe the possibility of catalysis by a Ru nanoparticle under the harshest conditions (*P*_H2_ = 8 MPa, *T* = 160 °C, *t* = 96 h). The catalytic activity was not perturbed during the course of the reaction (**4n**: 71%; **5f**: 92%). This is in good contrast to previous results using the less stable precatalyst **2a** ^38^, in which only marginal hydrogenation of **3m** and **3n** took place. In general, the more sterically demanding the amide, the less reactive the amide.

The hydrogenation rate of urea **3p** with **1b** was comparable to that obtained with **1a**, while urethane derivative **3q** was hydrogenated more effectively using **1b** than **1a** (Fig. 4b). Methanol was produced in ca. 60–70% yield in both cases, along with **4a** and **5a** in almost quantitative yields. These reactions are vitally important to the methanol economy^8, 10, 11, 23, 24^, as urea specifically is an excellent chemical reservoir and carrier of CO_2_.

**Figure 5:**
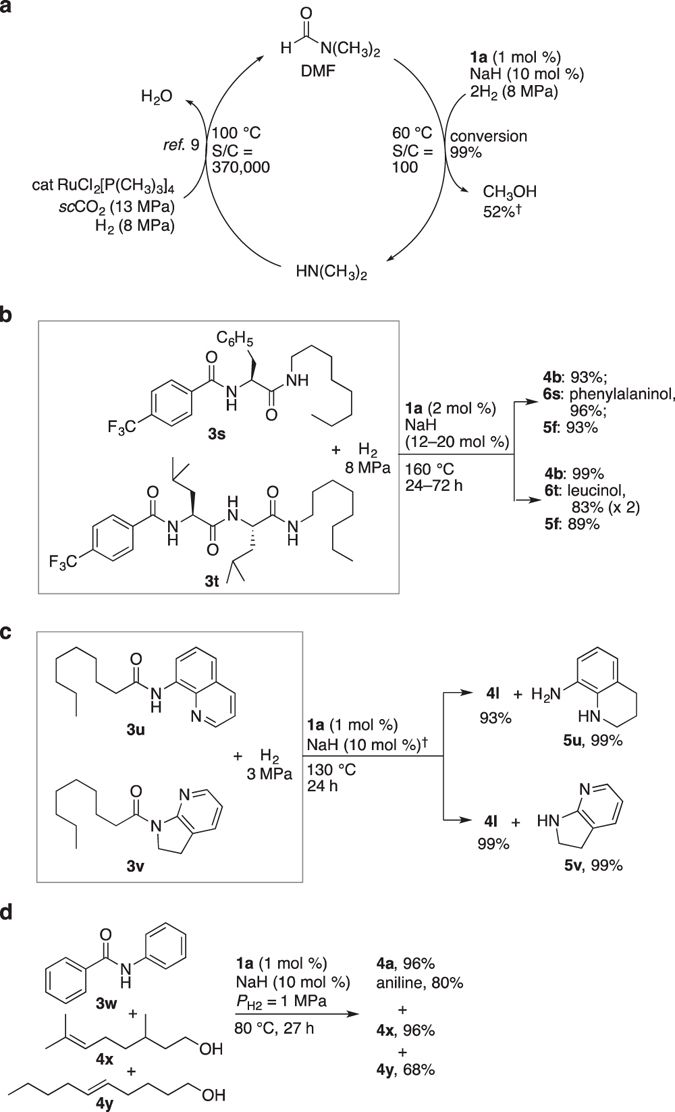
Multifaceted features of hydrogenation of small and highly functionalized amides. (**a**) DMF hydrogenation (**b**), Oligoamide hydrogenation. (**c**) Hydrogenation of **3u** and **3v** bearing directing groups. ^†^ **1a** was preactivated: *P*_H2(pre)_ = 1 MPa, *T*_pre_ = 160 °C, *t*_pre_ = 5 h. (**d**) Chemoselective hydrogenation of **3w** (**3w**:**4x**:**4y** = 100:50:50 mol %). See Supplementary S63 for experimental details.

Compared with **3p** and **3q**, hydrogenation of another CO_2_ derivative, DMF, proceeded far more smoothly, giving full conversion at 60 °C with *P*_H2_ = 8 MPa (Fig. 5a), and at 120 °C with *P*_H2_ = 2 MPa, producing CH_3_OH in ca. 60% yield in both cases. To hydrogenate tertiary amides DMF, **3j** and **3k**, and acetamide **3g**, preactivation of **1a** was required (*P*_H2(pre)_ = 1 MPa, *T*_pre_ = 160 °C, *t*_pre_ = 5 h) before addition of the corresponding **3** to the catalyst mixture. Since the chemical immobilization of CO_2_ as DMF^9^ and hydrogenation of DMF^10, 31^ have well been investigated in addition to the present result, a combination of the previous and present methods could provide an alternative route that benefits the methanol economy at low *T* and/or *P*_H2_, in a future effort to improve the method of recovering/recycling Me_2_NH.

To the best of our knowledge, the selective and stepwise cleavage of the different C–N bonds in oligoamides such as diamide **3s** and triamide **3t** (a dipeptide with protection at the N-terminus) was successfully accomplished for the first time (Fig. 5b). The chiral centers epimerized, giving a racemic mixture of β-amino alcohols **6s** and **6t**. More intricately functionalized, commercial polyamides available from Toray Co. (AQ nylon P-70 and T-70 (105–120 mg each)) were also hydrogenated (**1a**, 2.9 mg; *P*_H2_ = 8 MPa, *T* = 160 °C, *t* = 48 h), giving different monomer units, where the mass balance before and after the reaction was ca. 80% consistent (the structures of the two monomer units cannot be disclosed here due to a confidentiality agreement with Toray). This might pave a new avenue for recycling monomer derivatives, as an alternative to the depolymerization of polyamides (e.g., 6-nylon) that proceeded at 300 °C^50^.

Finally, the synthetic potential, including the chemoselectivity, of the hydrogenation was investigated. A directing group of C–H bond functionalization (8-aminoquinoline)^6^ in **3u** and of a catalytic amide aldol reaction (**5v**)^7^ in **3v** were easily detached from a main alkyl chain through the catalytic C–N bond cleavage by H_2_ (Fig. 5c). The partial structure of 8-aminoquinoline (pyridine) was concomitantly hydrogenated, giving **5u** in quantitative yield; in contrast, the pyridine moiety of **5v** also obtained quantitatively remained unreacted with H_2_. The hydrogenation method using **1a** is proven to have potential for converting rather thermodynamically stable, directing group-incorporated products to synthetically more useful alcohols. **5u** and **5v** are recyclable, and the former would also be reusable upon exploration of its rearomatization. Although the directing groups could be expected to strongly coordinate with a transition metal, H_2_ reacts even more favorably with the Ru center by taking advantage of a sterically confined H_2_-adsorption site. A more activated amide, anilide **3w**, was hydrogenated rapidly, giving near quantitative yields of **4a** and aniline with 0.25 mol % of **1a** and 6 mol % of NaH (*P*_H2_ = 0.5 MPa, *T* = 80 °C, *t* = 48 h; TON = ~400). Furthermore, the amide group of **3w** was hydrogenated (*P*_H2_ = 1 MPa, *T* = 80 °C, *t* = 27 h) preferentially even in the presence of a tri- and di-substituted olefins **4x** and **4y** (Fig. 5d). Since olefins are more likely to be hydrogenated via an inner-sphere mechanism^51^ (through direct interaction of the olefin with a metal center), these results again justify an outer-sphere mechanism^18–21, 26–29, 33, 34, 37, 38, 42–44^ that operates specifically for the hydrogenation of amides in preference to olefins, at least under mild conditions.

In conclusion, the effectiveness of a versatile “sterically confined bipyridine-Ru framework” for the C–N bond and C=O bond cleavage via hydrogenation of a variety of amides (from DMF to polyamides including diamides, triamides, and the synthetic polymers) under both mild and harsh reaction conditions has been demonstrated. Chemoselective amide hydrogenation, as well as hydrogenation of compounds potentially useful for the “methanol economy” under mild conditions, was also accomplished. Bipyridine (bpy) bearing two CH_2_ groups, two phosphorus atoms with sterically bulky alkyl substituents, and Ru are the unique constituents of the H_2_-adsorption site. These groups and elements are cooperative (to facilitate dearomatization and hydrogenation of bpy through deprotonation of the CH_2_ groups) and crucial for inducing robust ((*P*,(*N*, *N*)_bpy_, *P*)Ru structure which is electronically and sterically stabilized) and diverse (structures derived by different degrees of partial or full hydrogenation of bpy) catalysts. Further improvement of the robust (pre)catalyst based on our concept, including adopting a coordinatively-saturated Ru center, may significantly benefit the development of a better-performing catalyst for amide hydrogenation producing nonstandard peptides of pharmaceutically great importance, and even for facilitating a general method for the selective C=O bond cleavage of amide bonds.

## Methods

### Typical hydrogenation procedure

Under a continuous Ar flow, RUPIP2 (**1a**) (2.94 mg, 0.005 mmol), NaH (55% oil dispersion, 1.31 mg, 0.03 mmol), anhydrous toluene (1.5 mL), *N*-benzylbenzamide (**3a**) (105.6 mg, 0.5 mmol) and a magnetic stirring bar were placed in a dried Teflon tube (21 mL capacity). The Teflon tube was quickly inserted into an autoclave, and the autoclave inside was purged 10 times with hydrogen gas (1 MPa). The autoclave was pressurized with a 1 MPa of hydrogen gas at 25 °C, and heated at 110 °C for 24 h under stirring (800 rpm). The autoclave was cooled to ~25 °C in an ice–water bath, and the reaction mixture was quenched with a 2.0 M Et_2_O solution of HCl (15 μL, 0.03 mmol). The organic phase was removed in vacuo (ca. 100 mmHg, 40 °C). The residue was diluted with CDCl_3_, and analyzed by ^1^H NMR. The yields of benzyl alcohol (**4a**) (81%) and benzylamine (**5a**) (81%) were calculated based on the integral ratio among the signals of these compounds with respected to an internal standard (1,1,2,2-tetrachloroethane).

## Electronic supplementary material

Supplementary materials
