# Supplementary material for: Multifaceted catalytic hydrogenation of amides via diverse activation of a sterically confined bipyridine–ruthenium framework
Source: Sci Rep. 2017 May 16;7:1586. doi: 10.1038/s41598-017-01645-z (PMC5434022; doi:10.1038/s41598-017-01645-z)
Supplement: Supplementary file 1 — Supplementary materials [file 41598_2017_1645_MOESM1_ESM.pdf]

Supplementary Information

Multifaceted catalytic hydrogenation of amides via diverse activation of a sterically confined bipyridine–ruthenium framework

Takashi Miura, Masayuki Naruto, Katsuaki Toda, Taiki Shimomura, and Susumu Saito*

Graduate School of Science, Nagoya University, Chikusa, Nagoya 464-8602, Japan.

Correspondence to: saito.susumu@f.mbox.nagoya-u.ac.jp

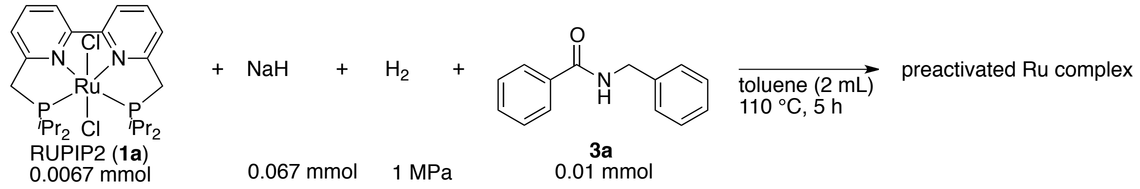

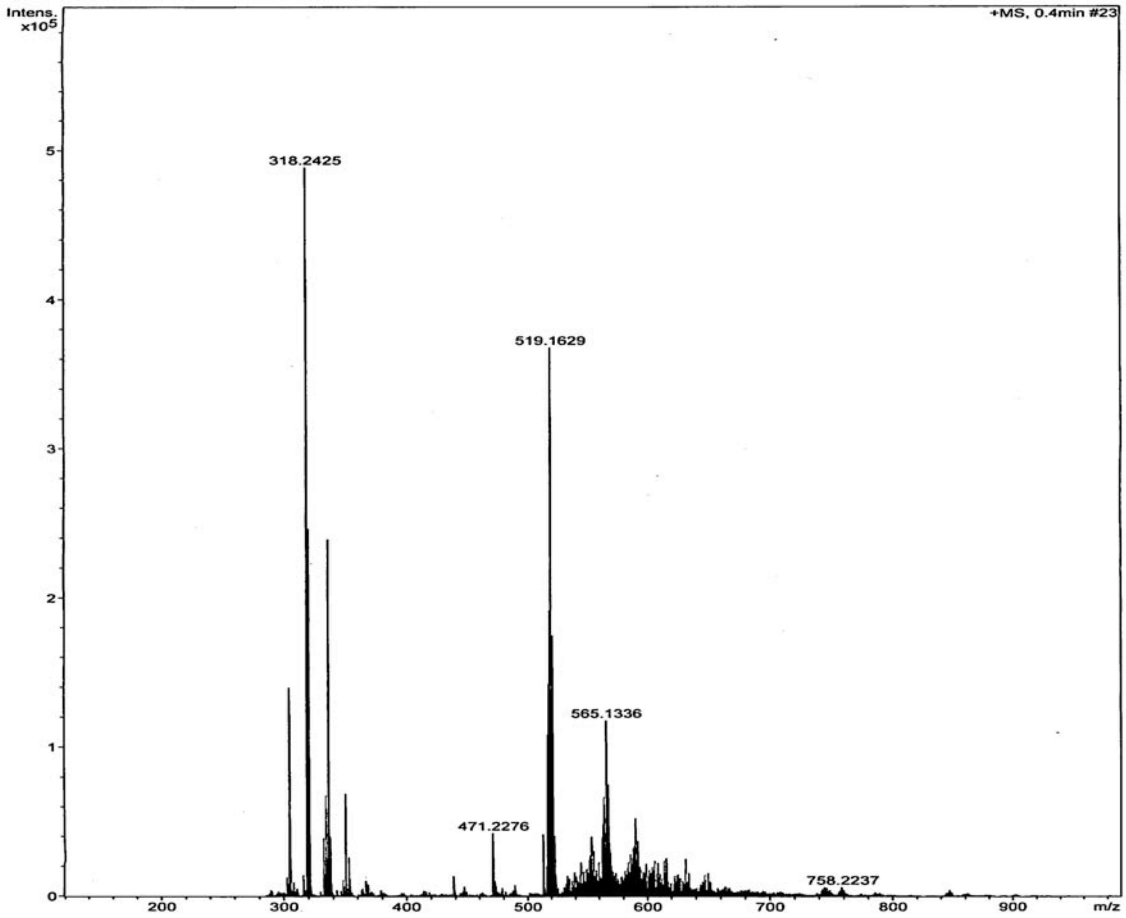

Supplementary Figure S1 | ESI-MS data of a preactivated catalyst: with *N*-benzylbenzamide (**3a**) ([**1a**]$_0$ = 3.3 mM, $P_{H2(pre)}$ = 1 MPa, T_{pre} = 110 °C).

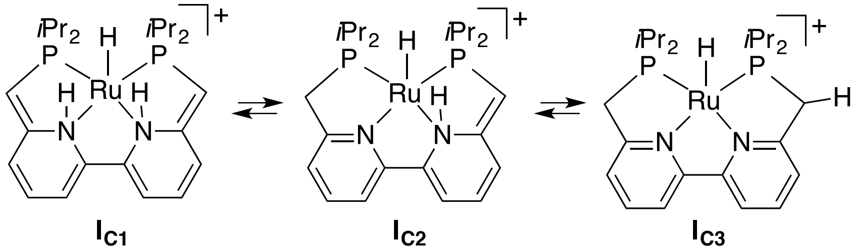

Calculated exact masses: **I$_{C1}$**–**I$_{C3}$** (519.1626)

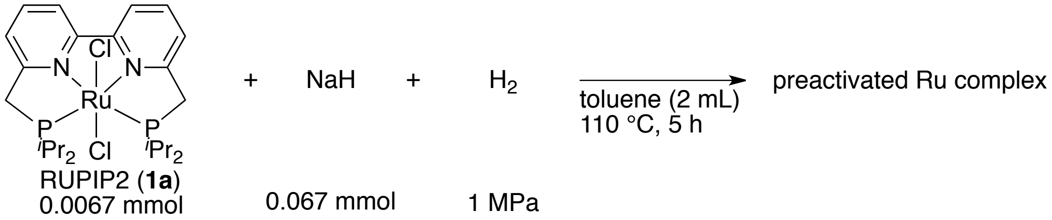

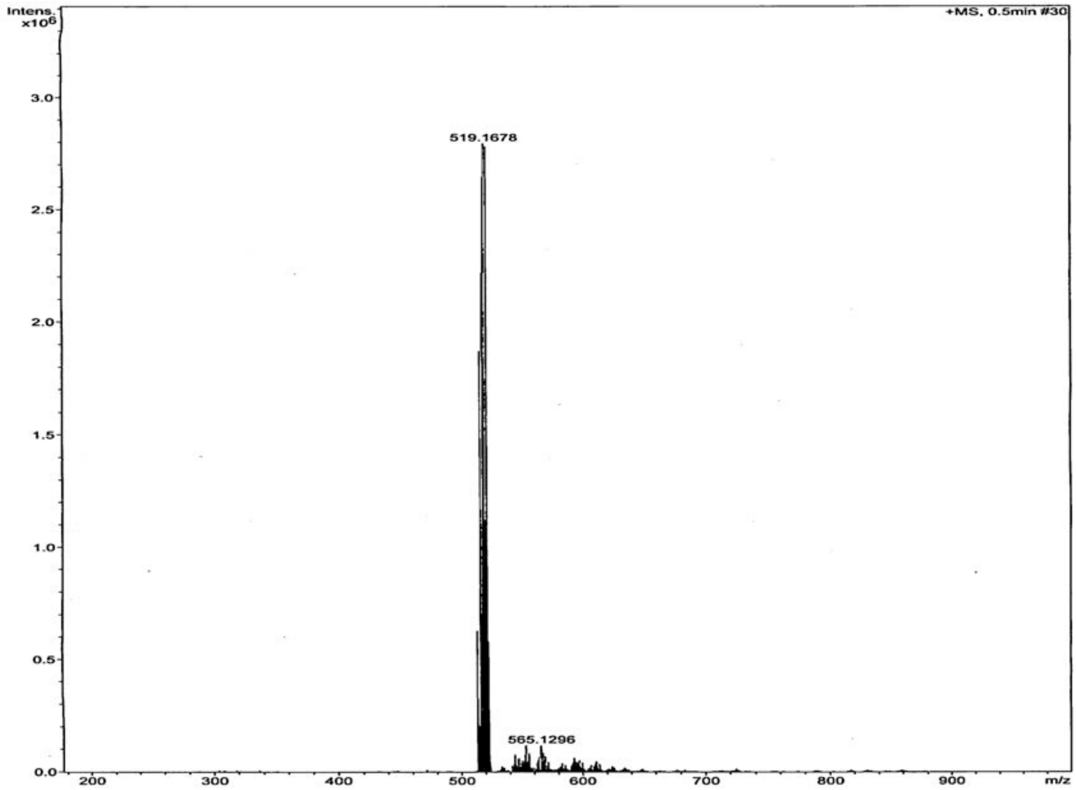

Supplementary Figure S2 | ESI-MS data of a preactivated catalyst: without *N*-benzylbenzamide (**3a**) ([**1a**]$_0$ = 3.3 mM, $P_{H2(pre)}$ = 1 MPa, T_{pre} = 110 °C).

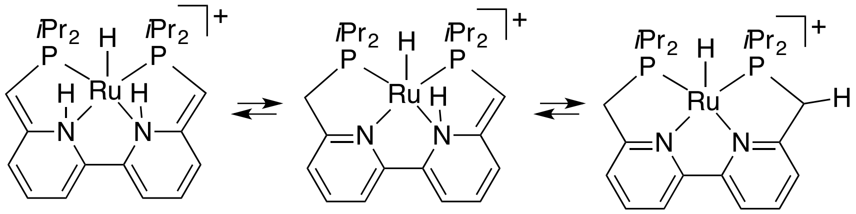

$\mathbf{I_{C1}}$ $\mathbf{I_{C2}}$ $\mathbf{I_{C3}}$

Calculated exact masses: $\mathbf{I_{C1}}$–$\mathbf{I_{C3}}$ (519.1626)

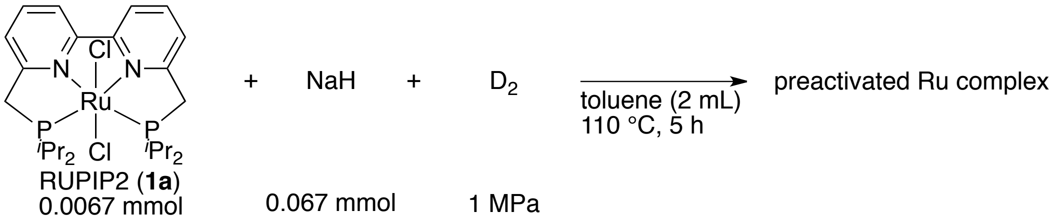

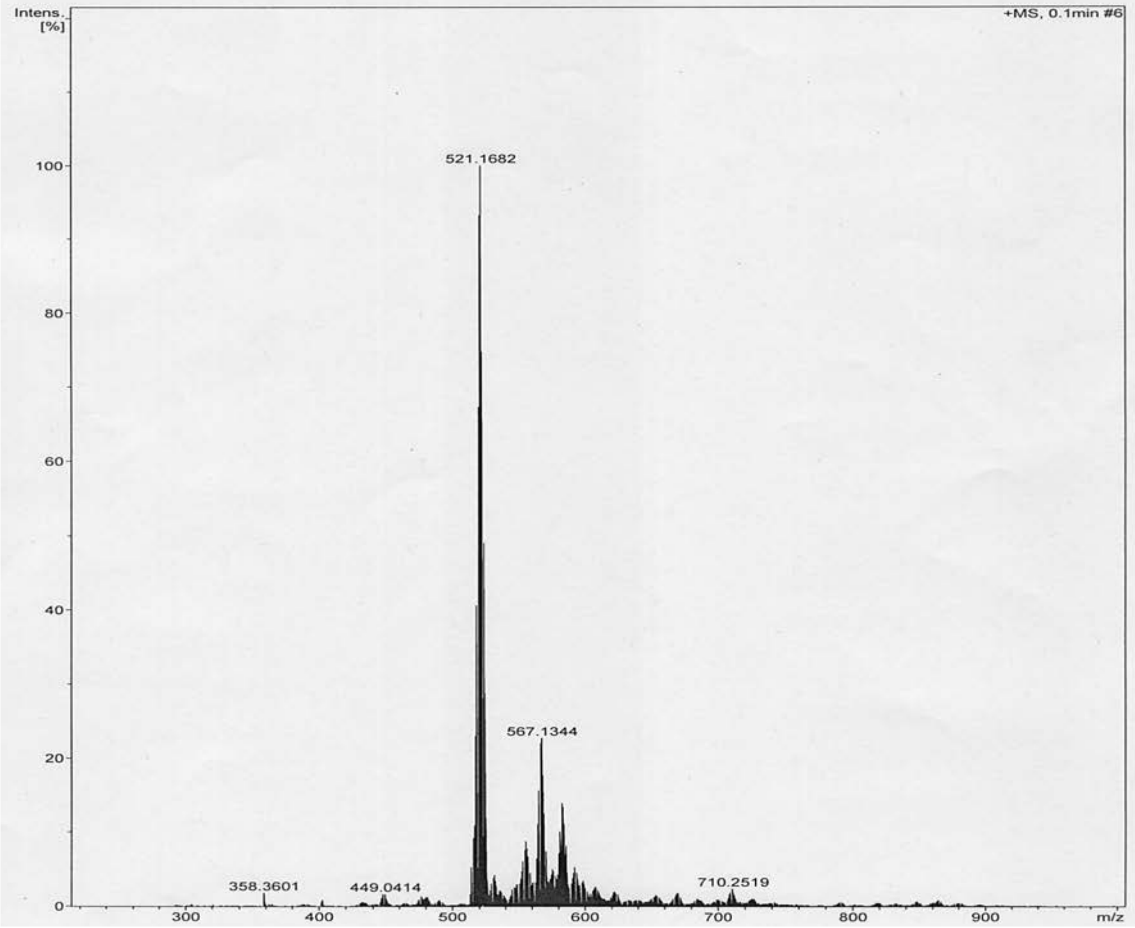

Supplementary Figure S3 | ESI-MS data of a preactivated catalyst: without *N*-benzylbenzamide (**3a**) ([**1a**]$_0$ = 3.3 mM, $P_{\mathrm{D2(pre)}}$ = 1 MPa, T_{pre} = 110 °C).

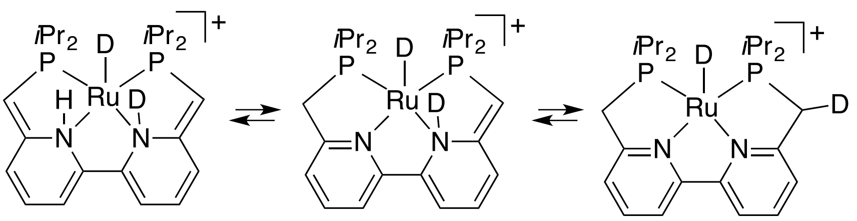

Calculated exact masses: **I**$_{\mathbf{C}}$-d_2 (521.1752)

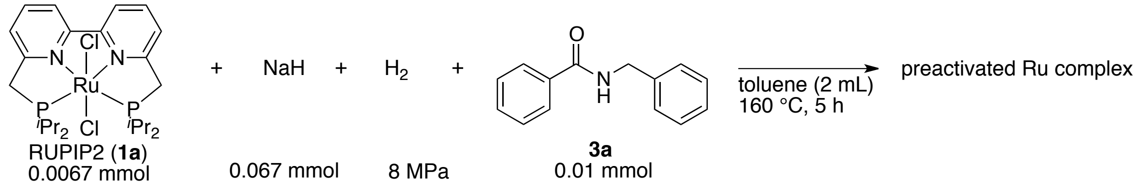

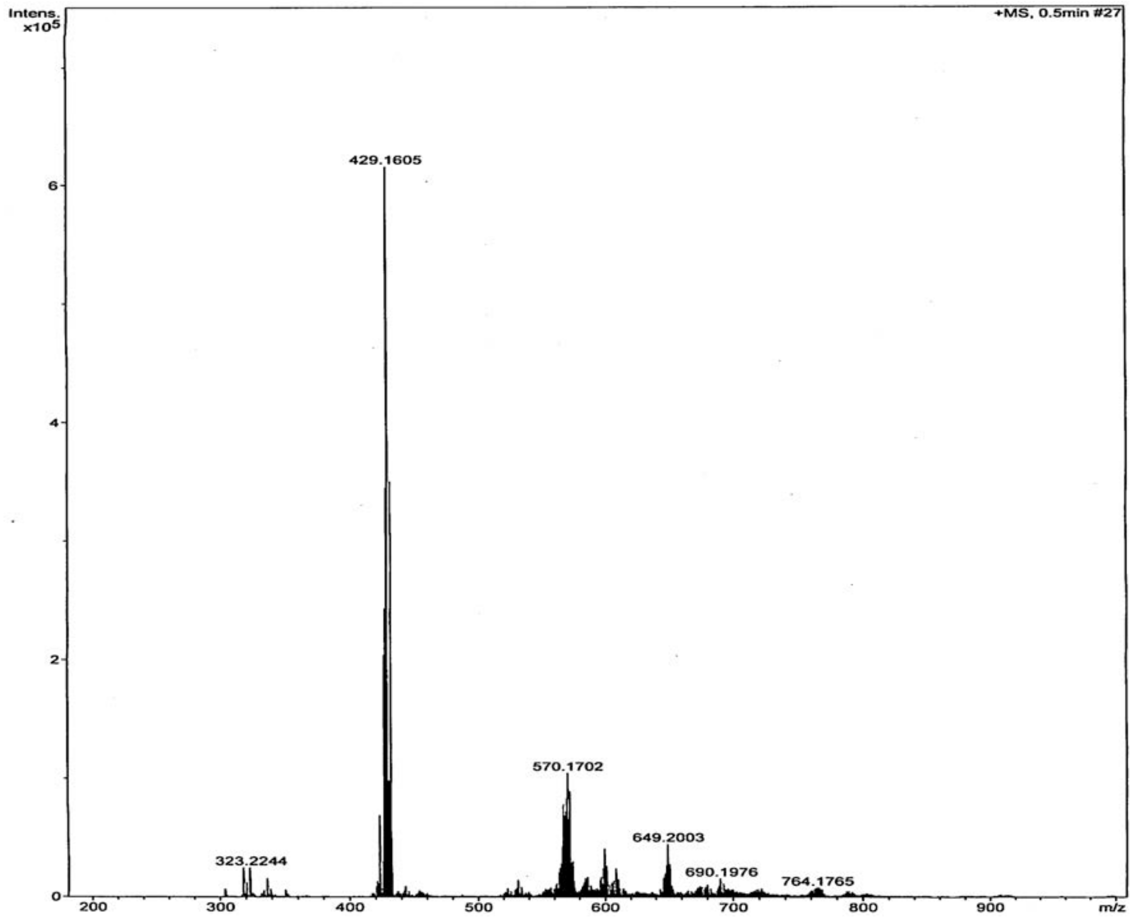

Supplementary Figure S4 | ESI-MS data of a preactivated catalyst: with *N*-benzylbenzamide (**3a**) ([**1a**]$_0$ = 3.3 mM, $P_{H2(pre)}$ = 8 MPa, T_{pre} = 160 °C).

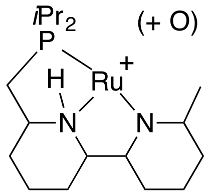

$\mathbf{I_E}$

Calculated exact masses: $\mathbf{I_E}$ (429.1603)

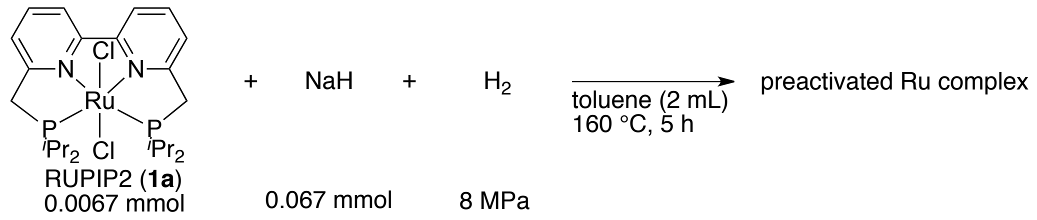

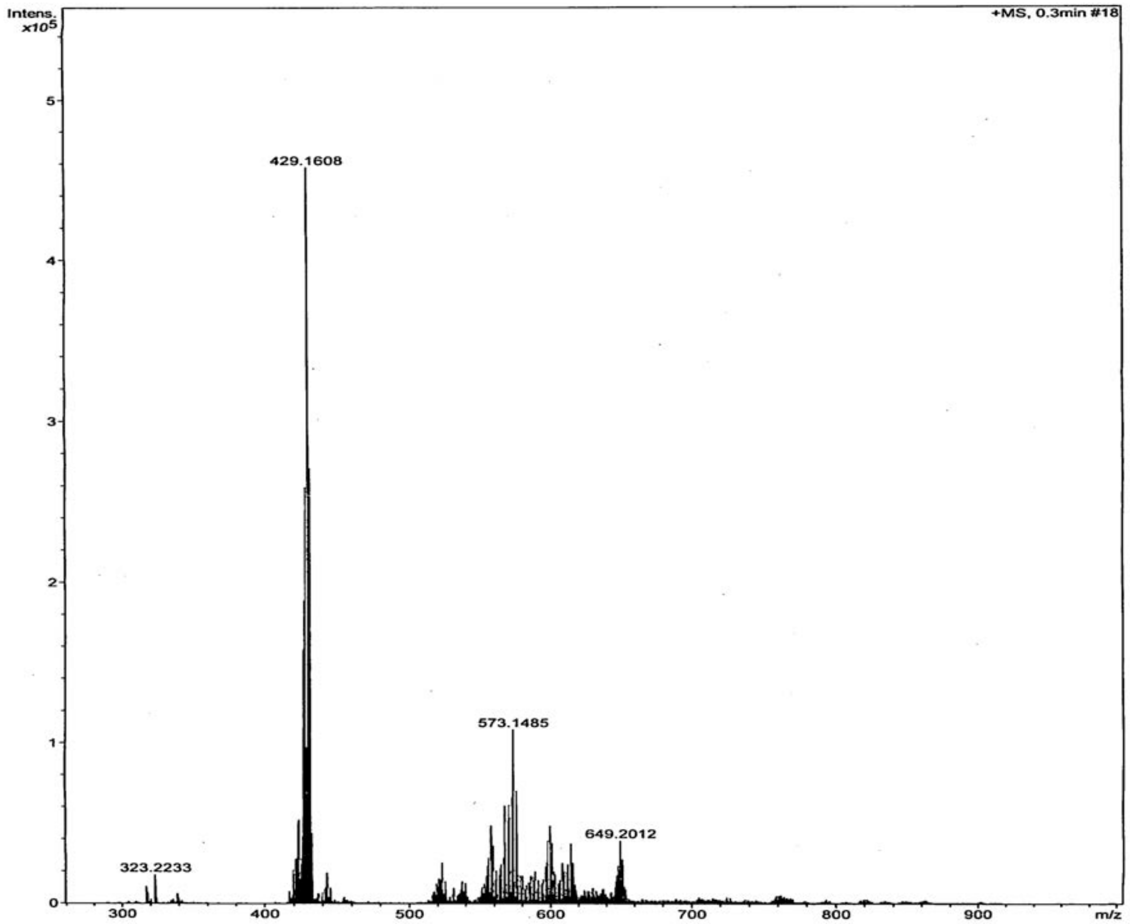

Supplementary Figure S5 | ESI-MS data of a preactivated catalyst: without *N*-benzylbenzamide (**3a**) ([**1a**]$_0$ = 3.3 mM, $P_{H2(pre)}$ = 8 MPa, T_{pre} = 160 °C).

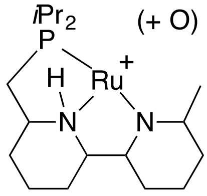

I$_E$

Calculated exact masses: **I$_E$** (429.1603)

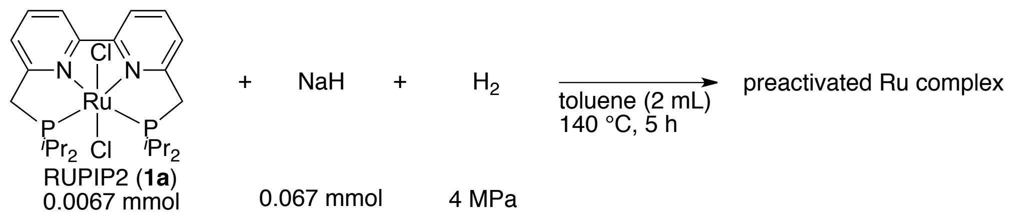

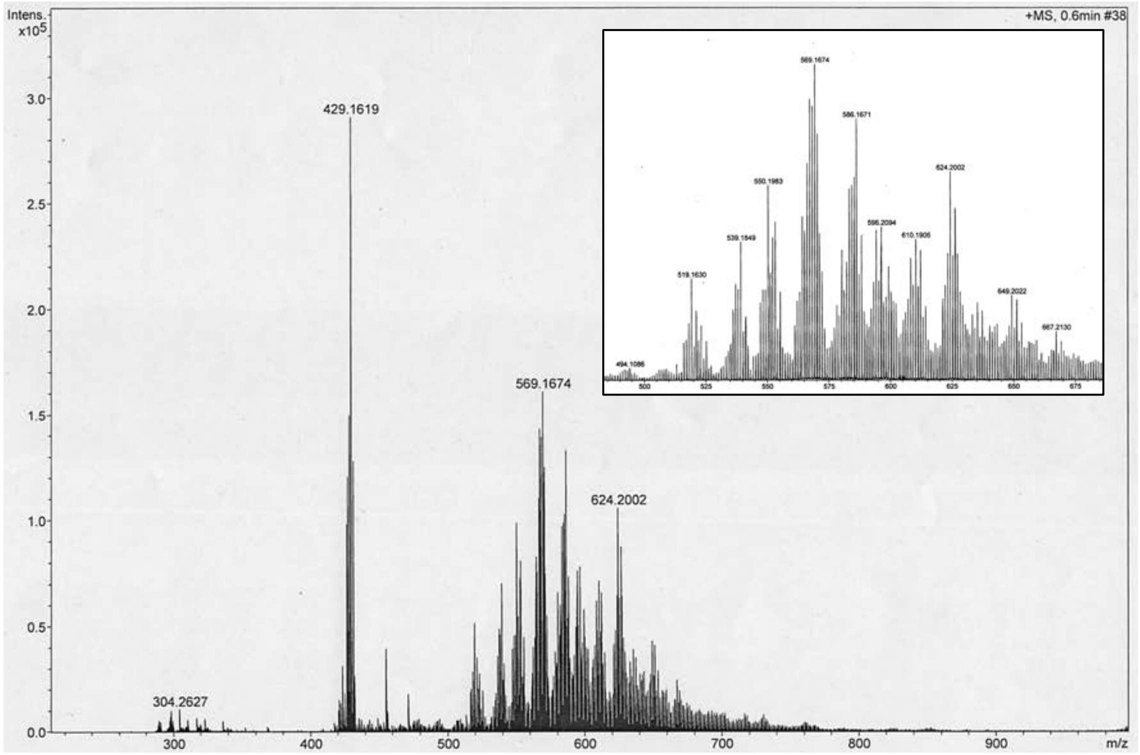

Supplementary Figure S6 | ESI-MS data of a preactivated catalyst: without *N*-benzylbenzamide (**3a**) ([**1a**]$_0$ = 3.3 mM, $P_{H2(pre)}$ = 4 MPa, T_{pre} = 140 °C).

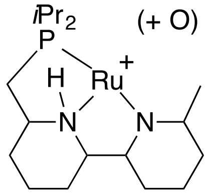

I_E

Calculated exact masses: **I_E** (429.1603)

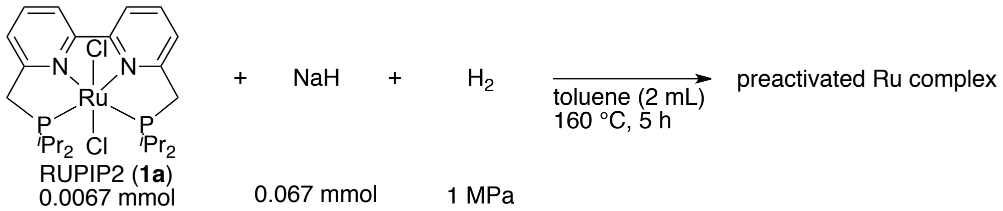

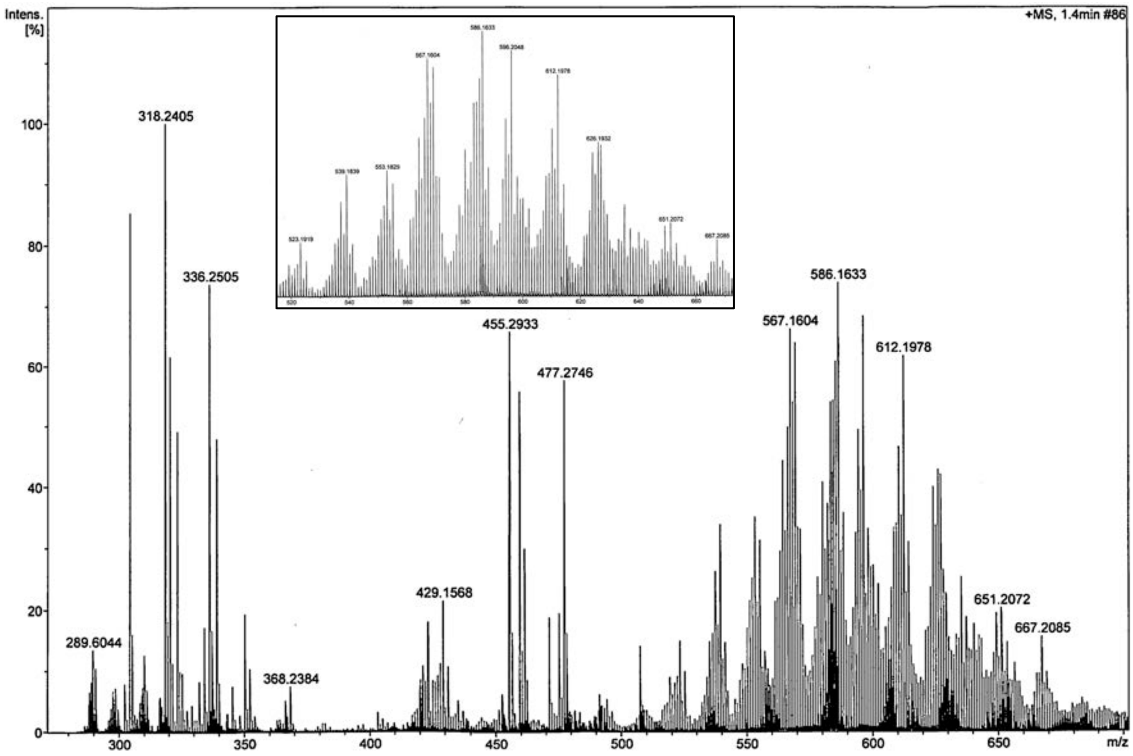

Plausible tautomers

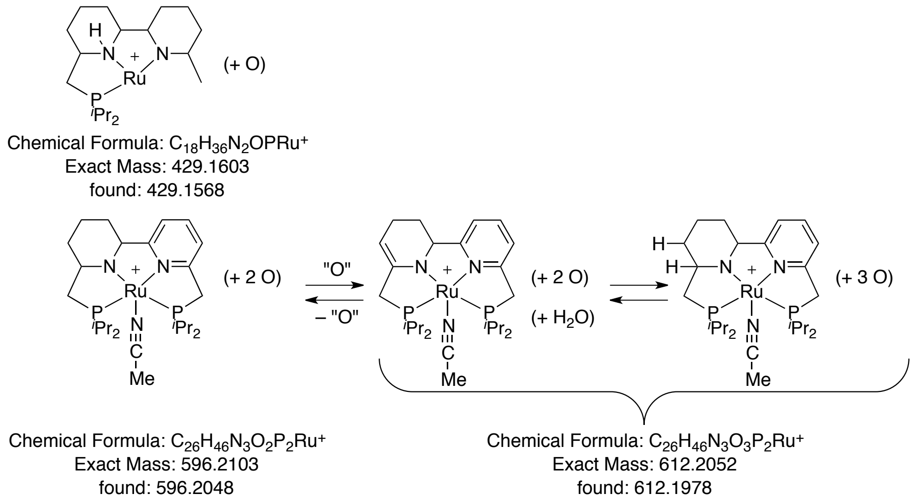

Supplementary Figure S7 | ESI-MS data of a preactivated catalyst: without *N*-benzylbenzamide (**3a**) ([**1a**]$_0$ = 3.3 mM, $P_{H2(pre)}$ = 1 MPa, T_{pre} = 160 °C).

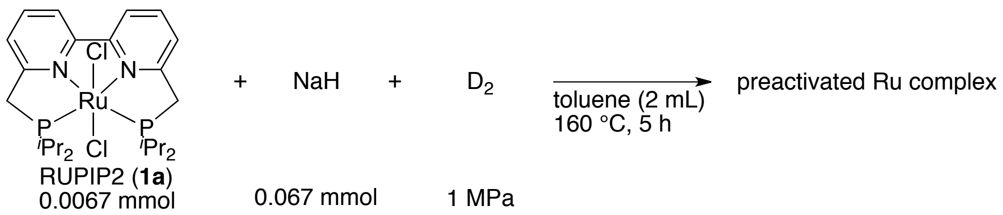

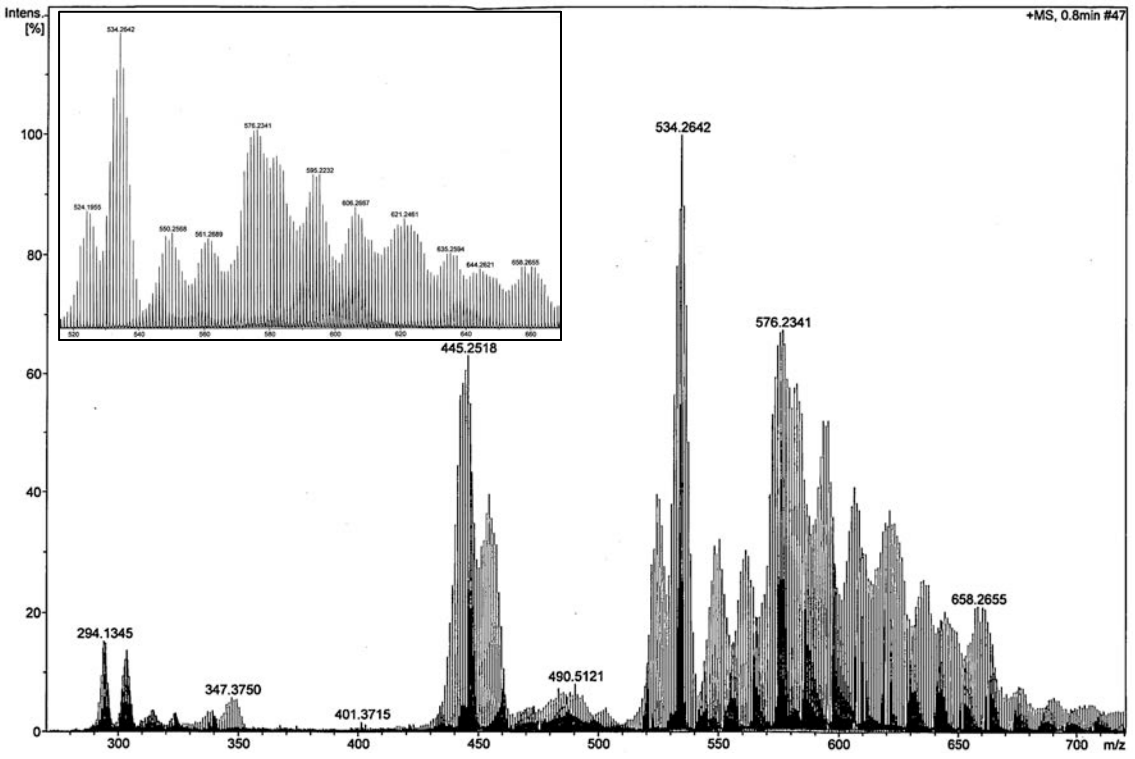

Plausible tautomers

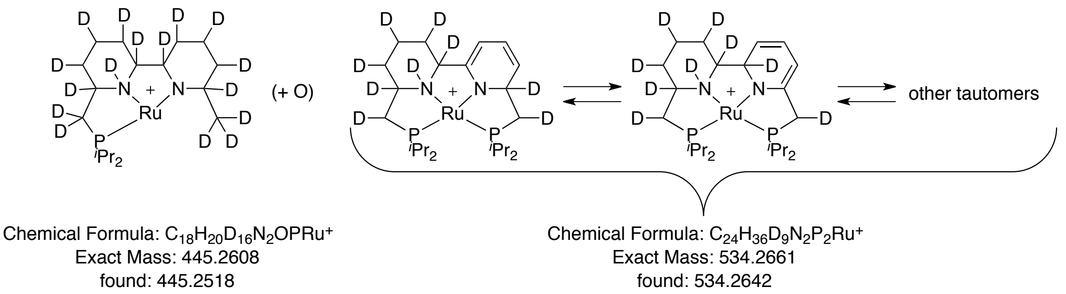

Supplementary Figure S8 | ESI-MS data of a preactivated catalyst: without *N*-benzylbenzamide (**3a**) ([**1a**]$_0$ = 3.3 mM, $P_{D2(pre)}$ = 1 MPa, T_{pre} = 160 °C).

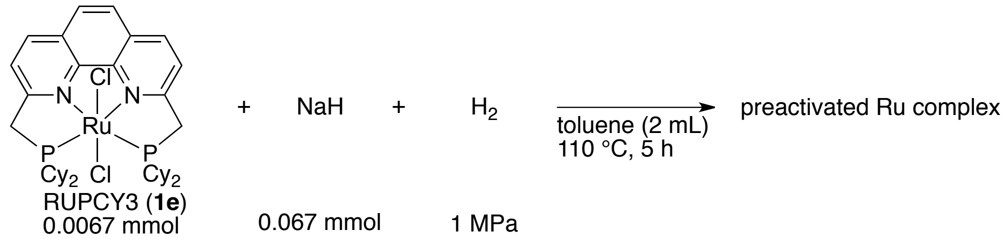

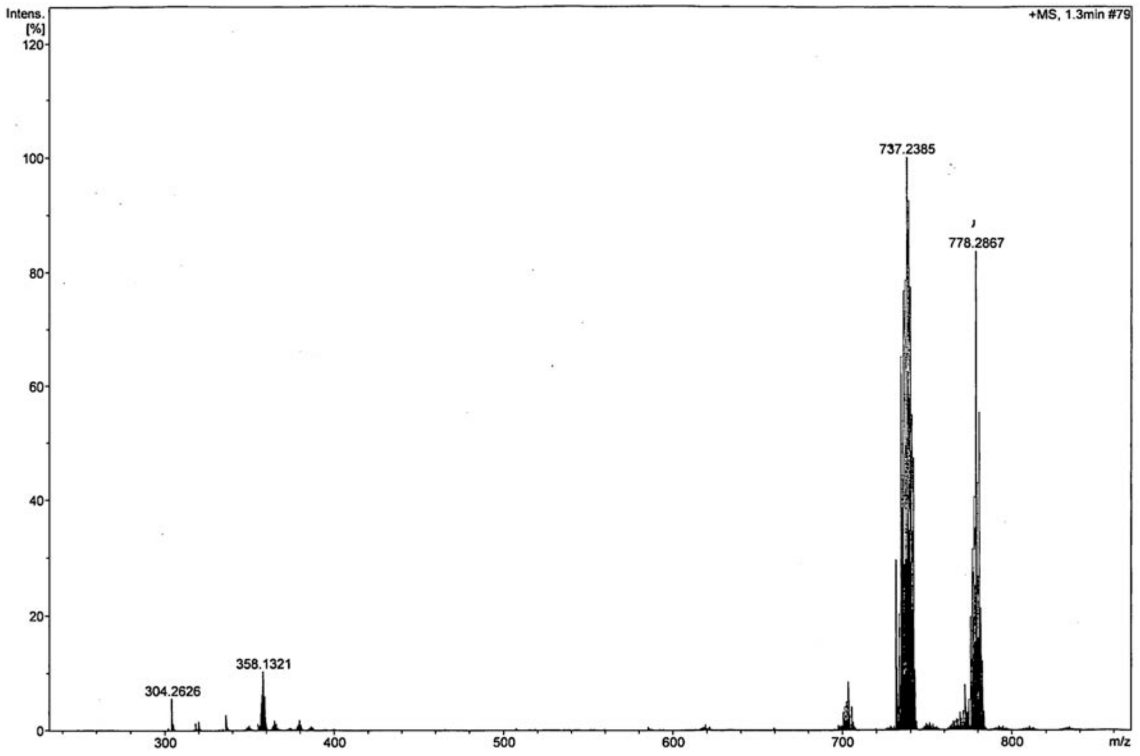

Plausible tautomers

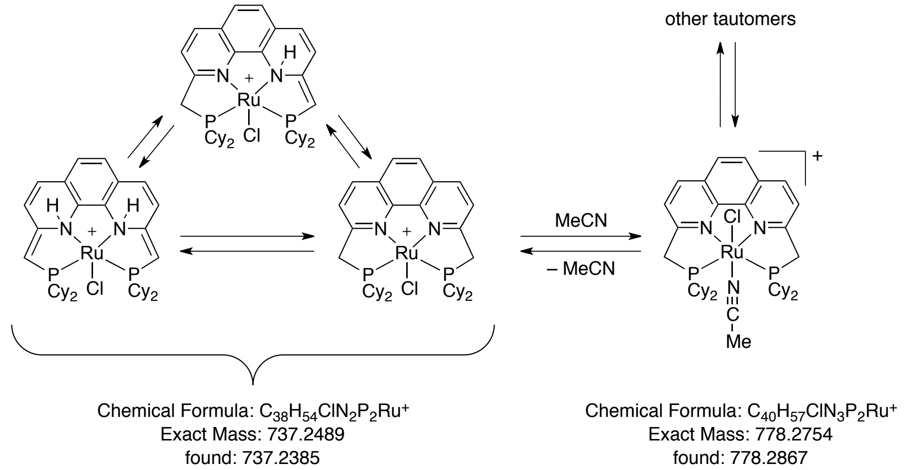

Supplementary Figure S9 | ESI-MS data of a preactivated catalyst: without *N*-benzylbenzamide (**3a**) ([**1e**]$_0$ = 3.3 mM, $P_{\mathrm{H2(pre)}}$ = 1 MPa, T_{pre} = 110 °C).

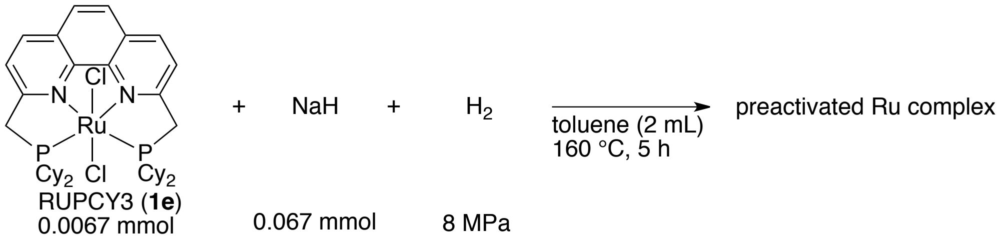

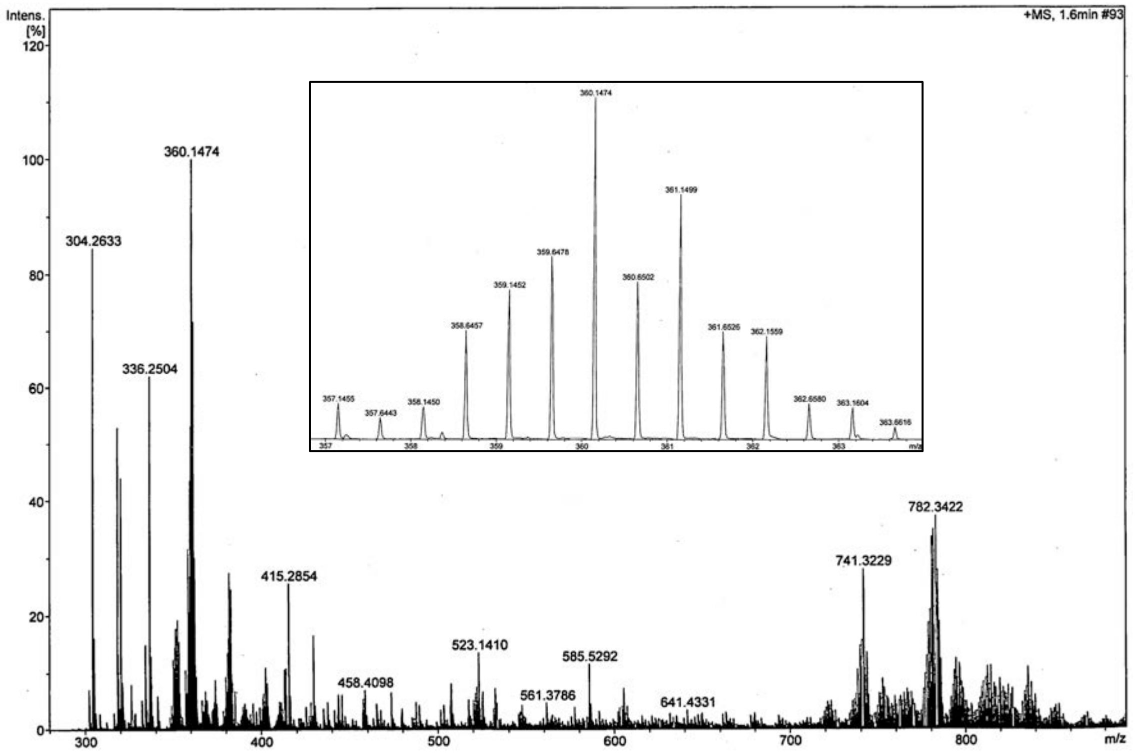

Plausible tautomers

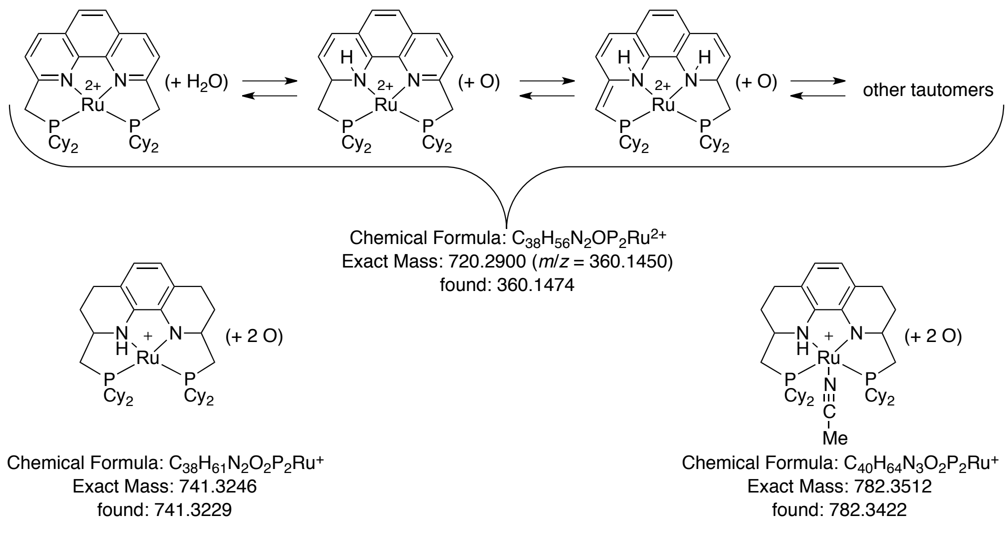

Supplementary Figure S10 | ESI-MS data of a preactivated catalyst: without *N*-benzylbenzamide (**3a**) ([**1e**]$_0$ = 3.3 mM, $P_{H2(pre)}$ = 8 MPa, T_{pre} = 160 °C).

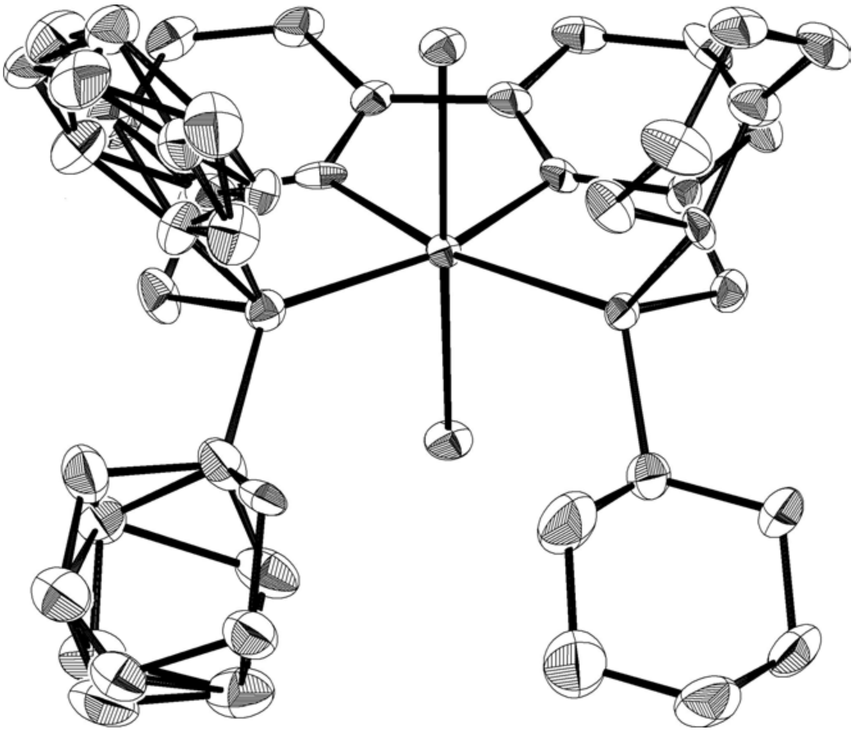

Supplementary Figure S11 | ORTEP drawing of RUPCY2 (**1b**) (50% probability ellipsoids). Hydrogen atoms were omitted for clarity

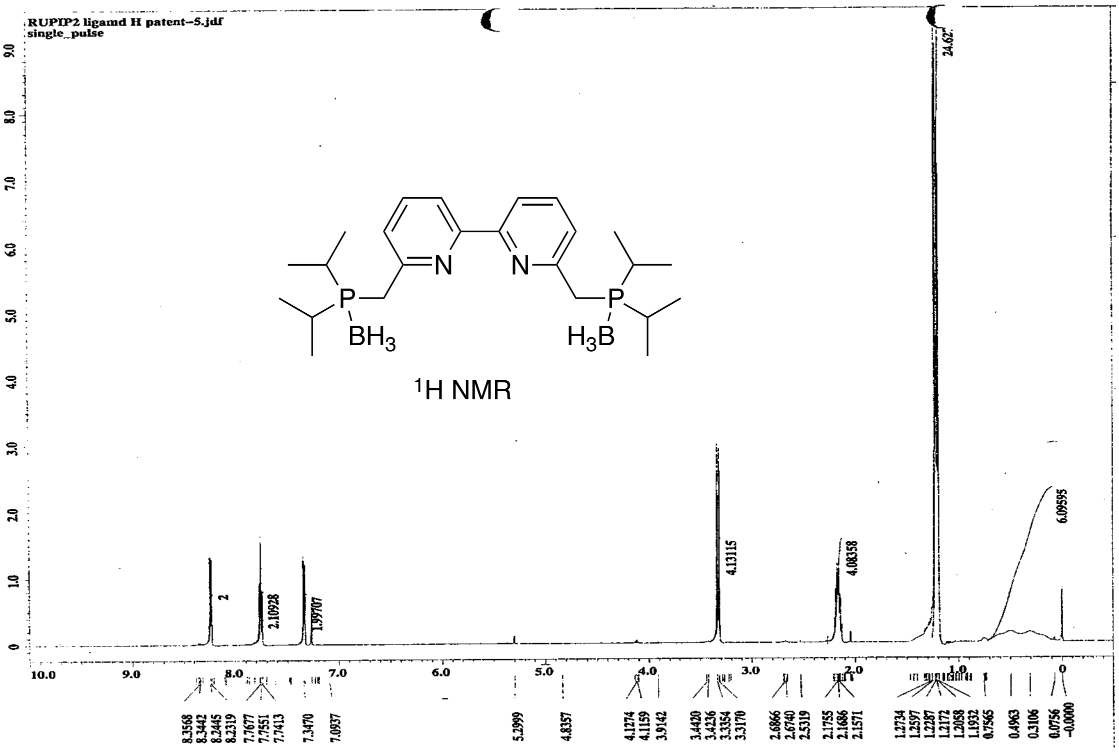

Supplementary Figure S12 | ^{1}H NMR spectrum of 6,6'-bis((diisopropylphosphino)methyl)-2,2'-bipyridine –diborane complex

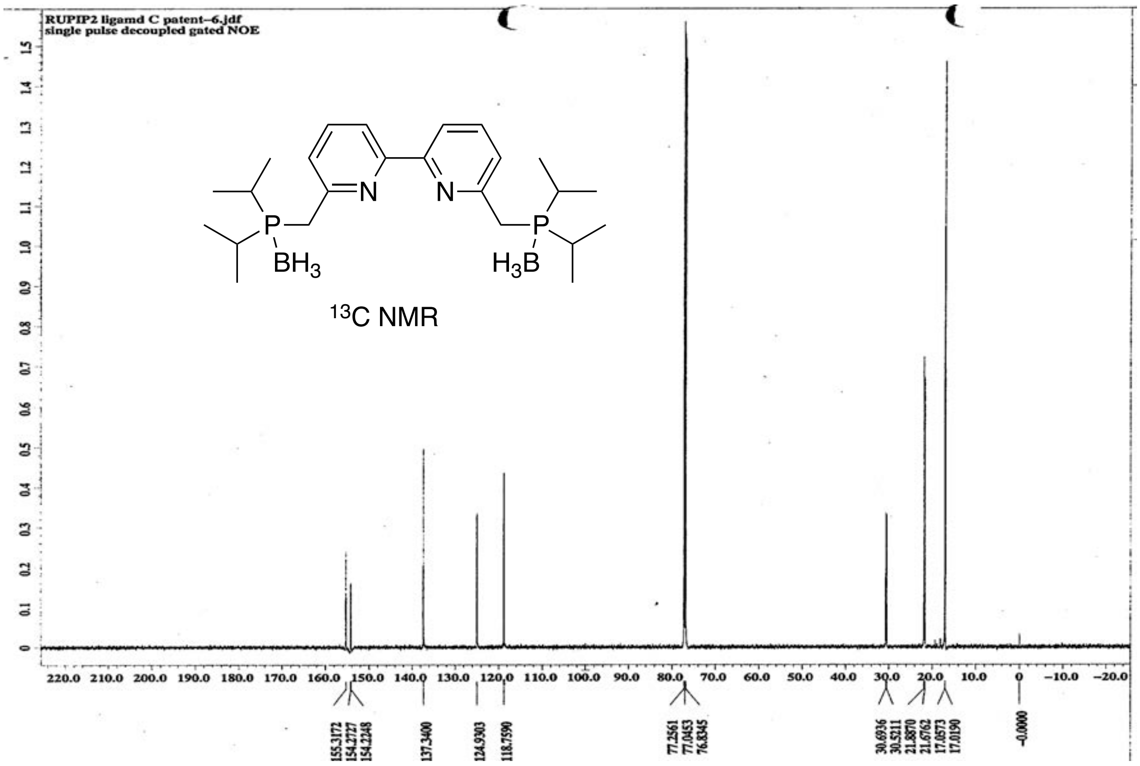

Supplementary Figure S13 | ^{13}C NMR spectrum of 6,6'-bis((diisopropylphosphino)methyl)-2,2'-bipyridine –diborane complex

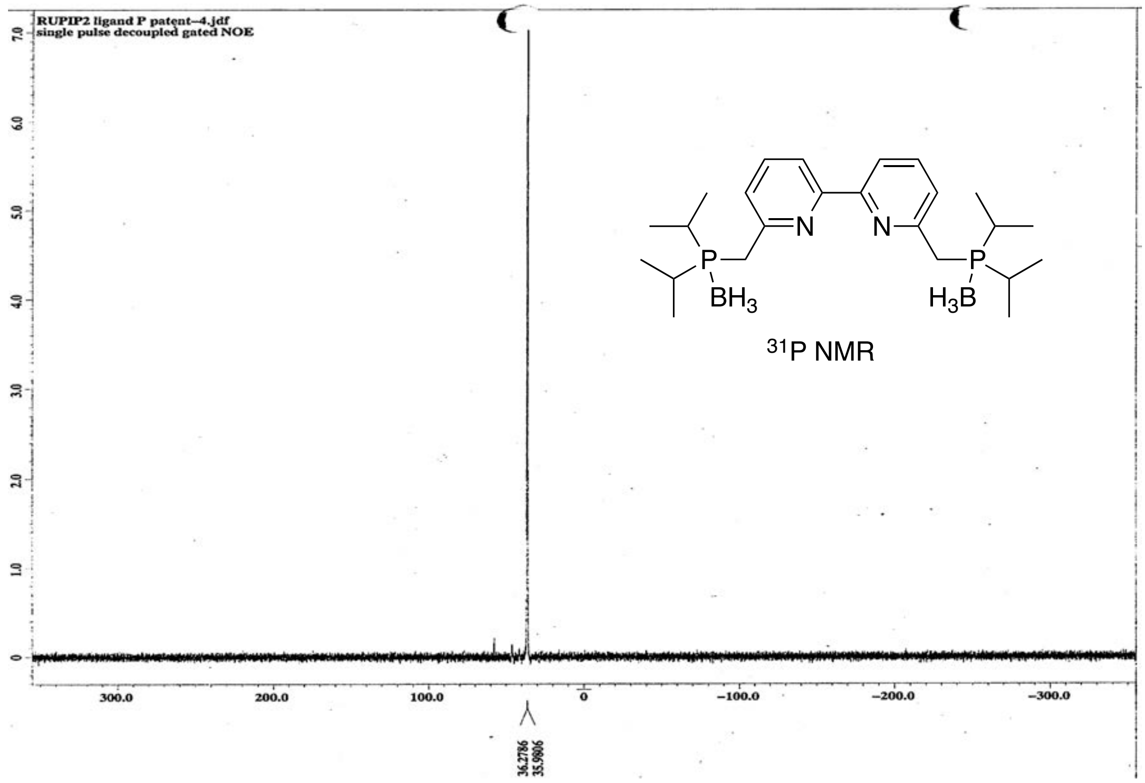

Supplementary Figure S14 | ^{31}P NMR spectrum of 6,6'-bis((diisopropylphosphino)methyl)-2,2'-bipyridine –diborane complex

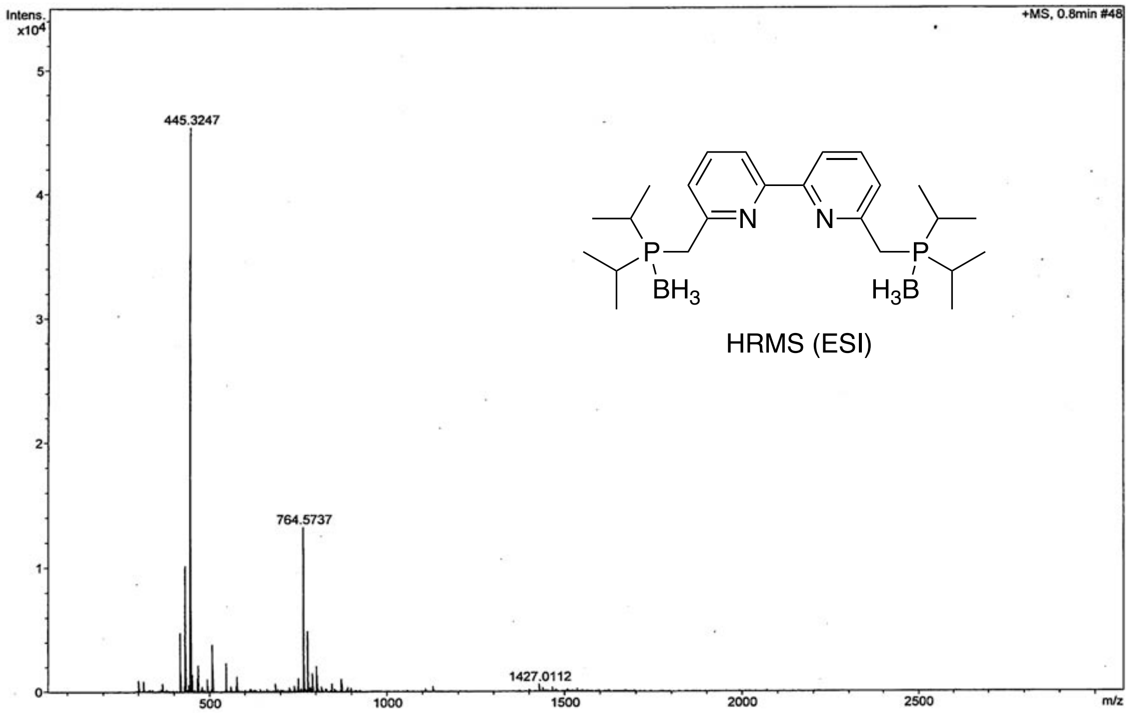

Supplementary Figure S15 | HRMS (ESI) of 6,6'-bis((diisopropylphosphino)methyl)-2,2'-bipyridine –diborane complex

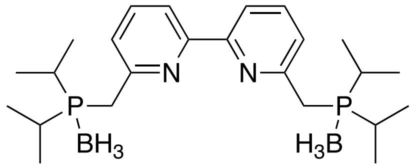

IR

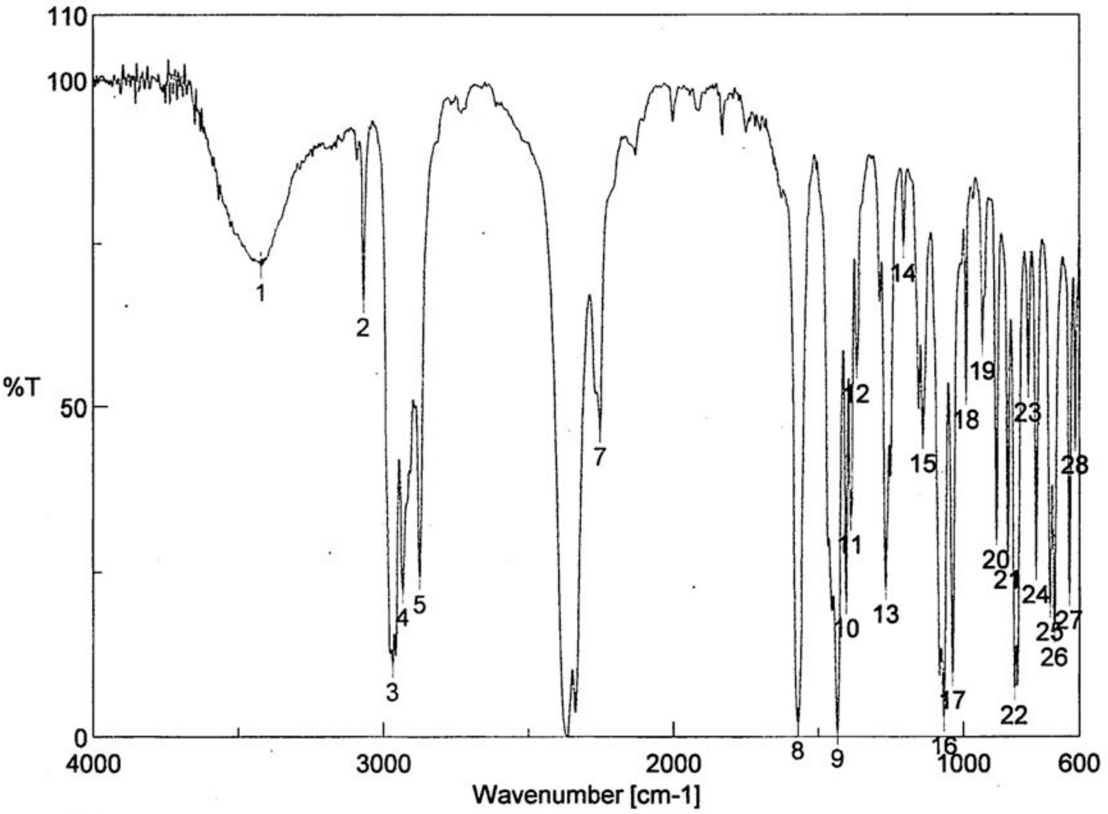

Supplementary Figure S16 | IR spectrum of 6,6'-bis((diisopropylphosphino)methyl)-2,2'-bipyridine –diborane complex (KBr)

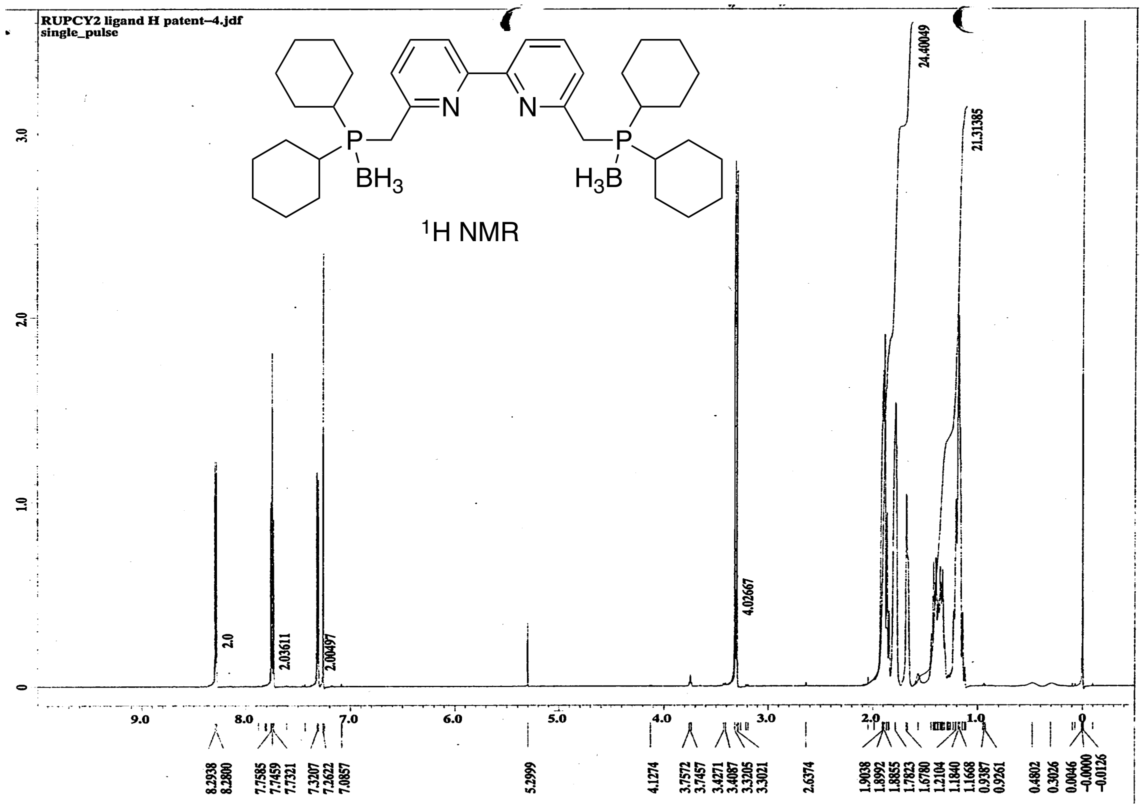

Supplementary Figure S17 | ^{1}H NMR spectrum of 6,6'-bis((dicyclohexylphosphino)methyl)-2,2'-bipyridine−diborane complex

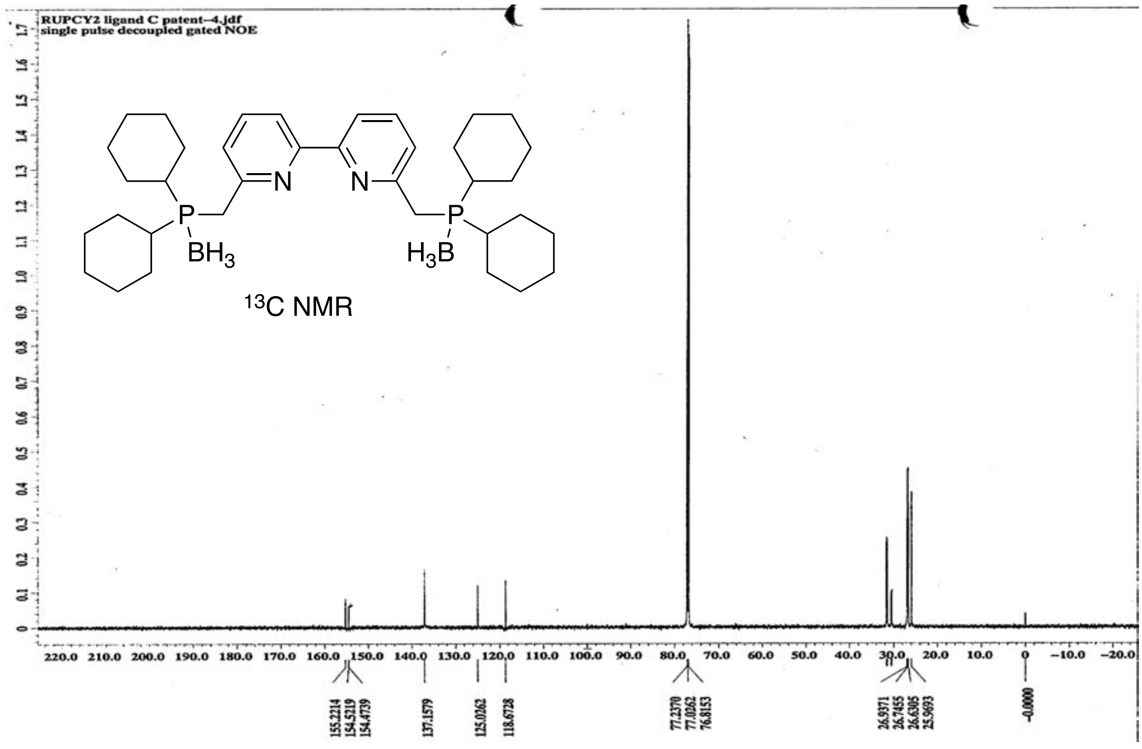

Supplementary Figure S18 | ^{13}C NMR spectrum of 6,6'-bis((dicyclohexylphosphino)methyl)-2,2'-bipyridine−diborane complex

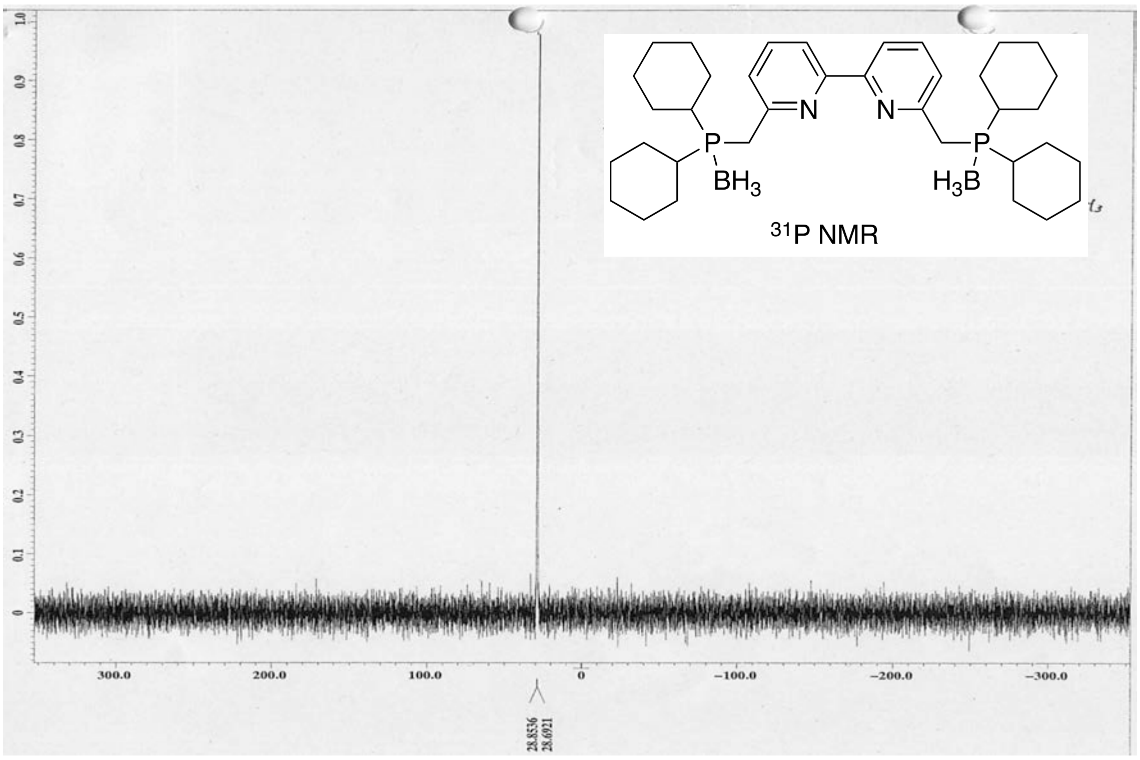

Supplementary Figure S19 | ^{31}P NMR spectrum of 6,6'-bis((dicyclohexylphosphino)methyl)-2,2'-bipyridine−diborane complex

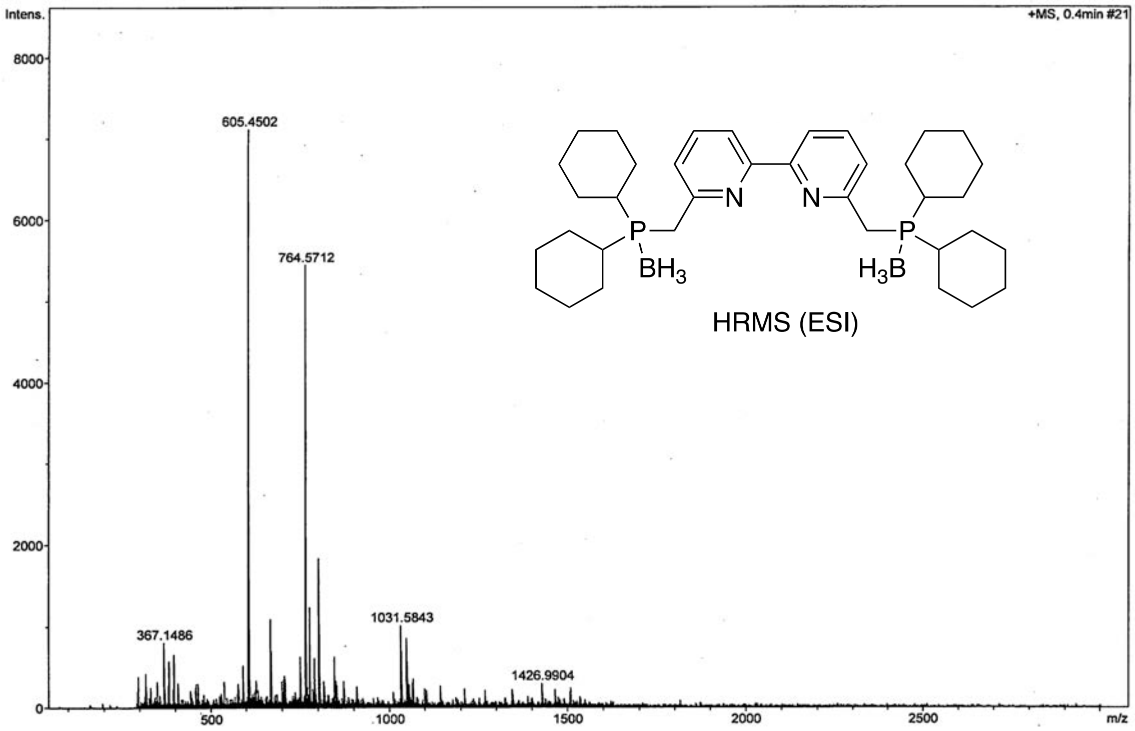

Supplementary Figure S20 | HRMS (ESI) of 6,6'-bis((dicyclohexylphosphino)methyl)-2,2'-bipyridine−diborane complex

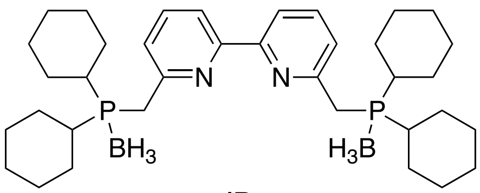

IR

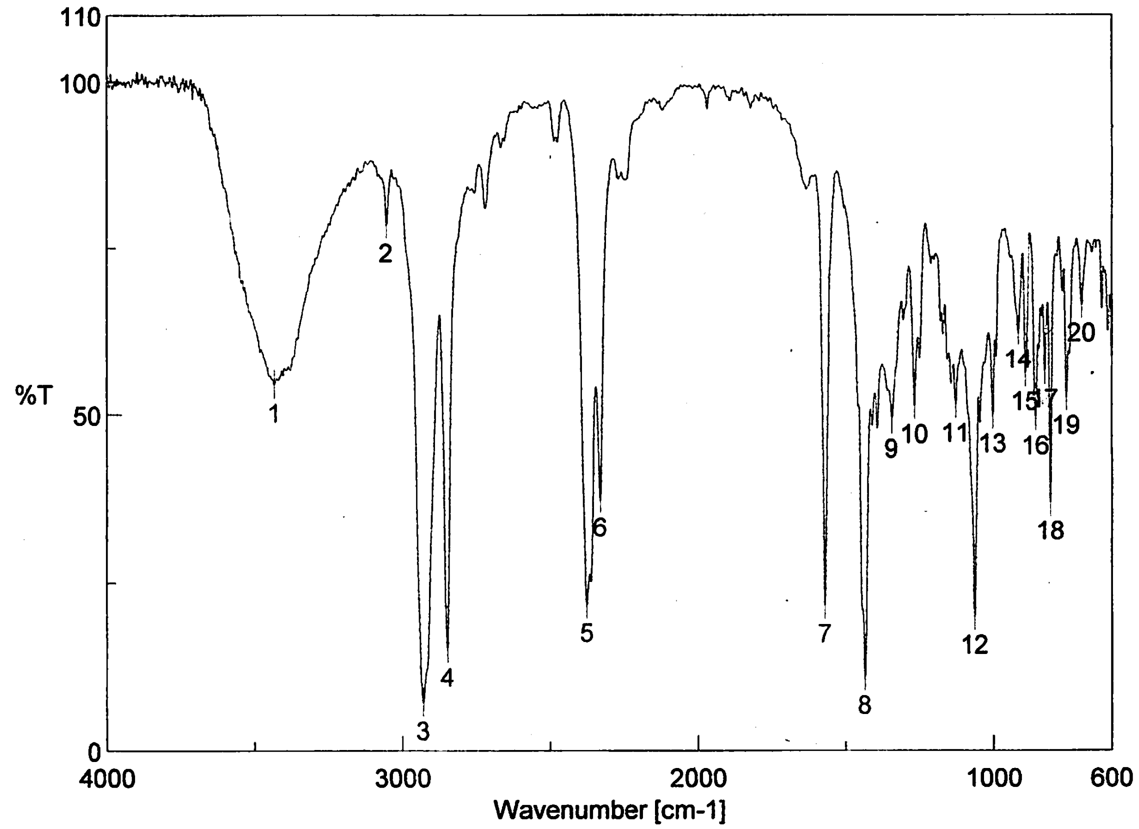

Supplementary Figure S21 | IR spectrum of 6,6'-bis((dicyclohexylphosphino)methyl)-2,2'-bipyridine−diborane complex (KBr)

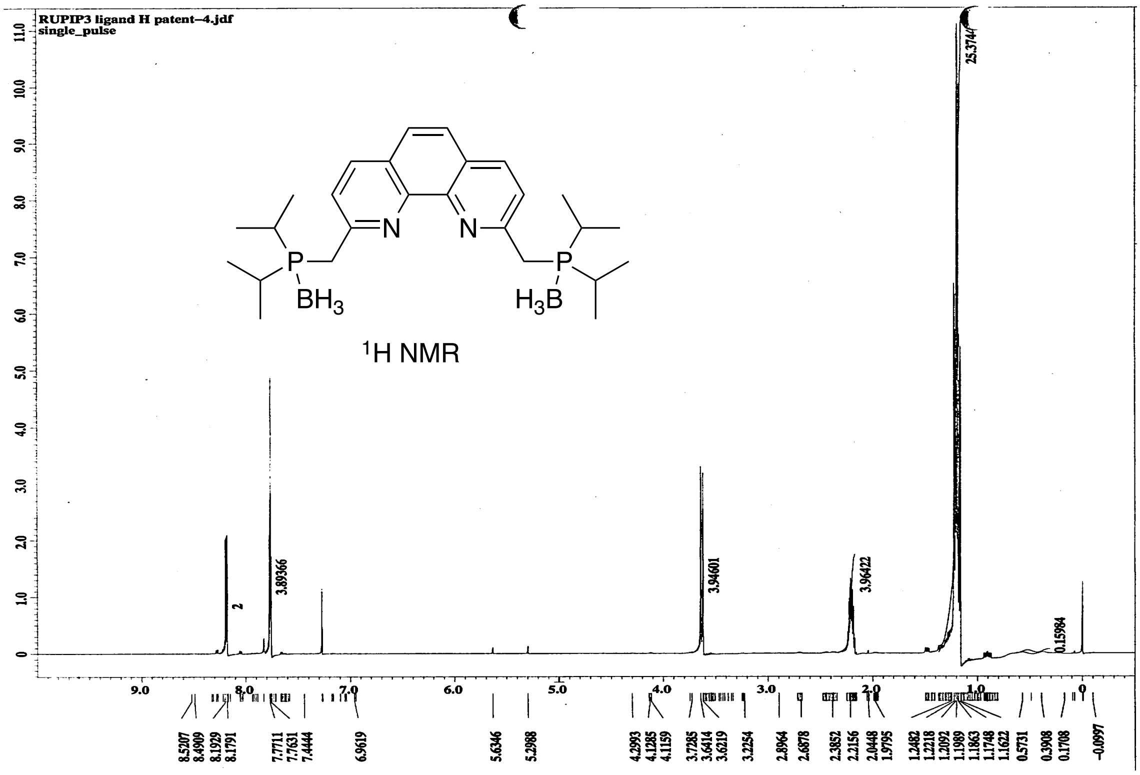

Supplementary Figure S22 | ^{1}H NMR spectrum of 2,9-bis((diisopropylphosphino)methyl)-1,10-phenanthroline−diborane complex

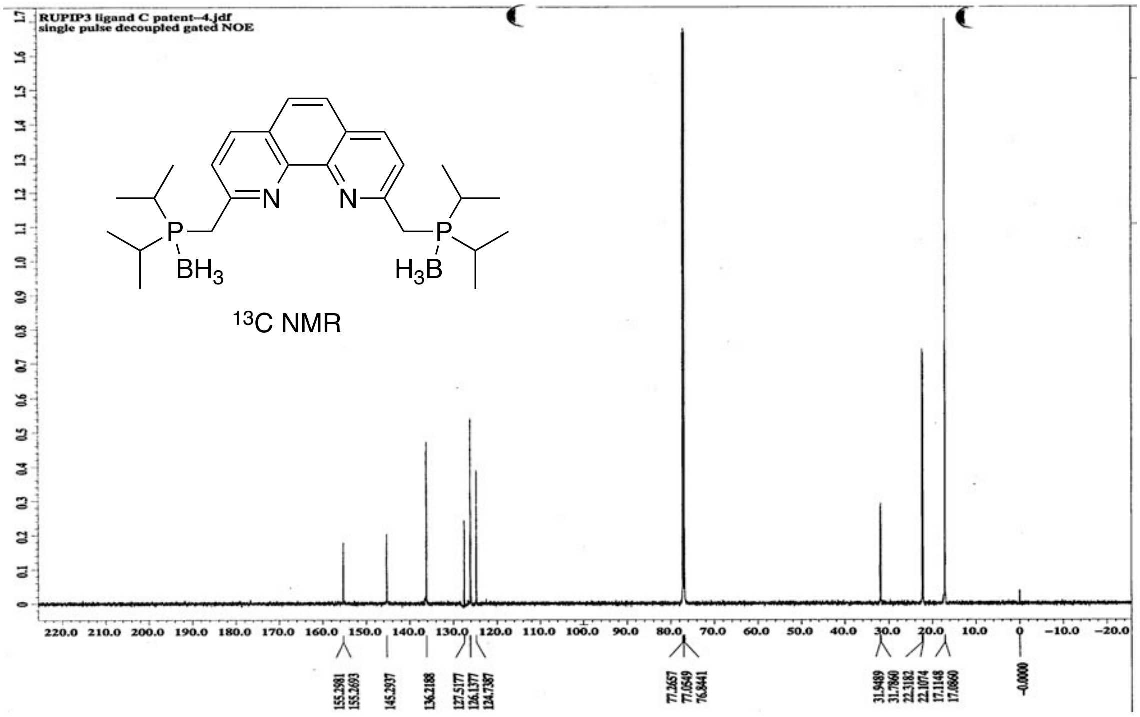

Supplementary Figure S23 | ^{13}C NMR spectrum of 2,9-bis((diisopropylphosphino)methyl)-1,10-phenanthroline−diborane complex

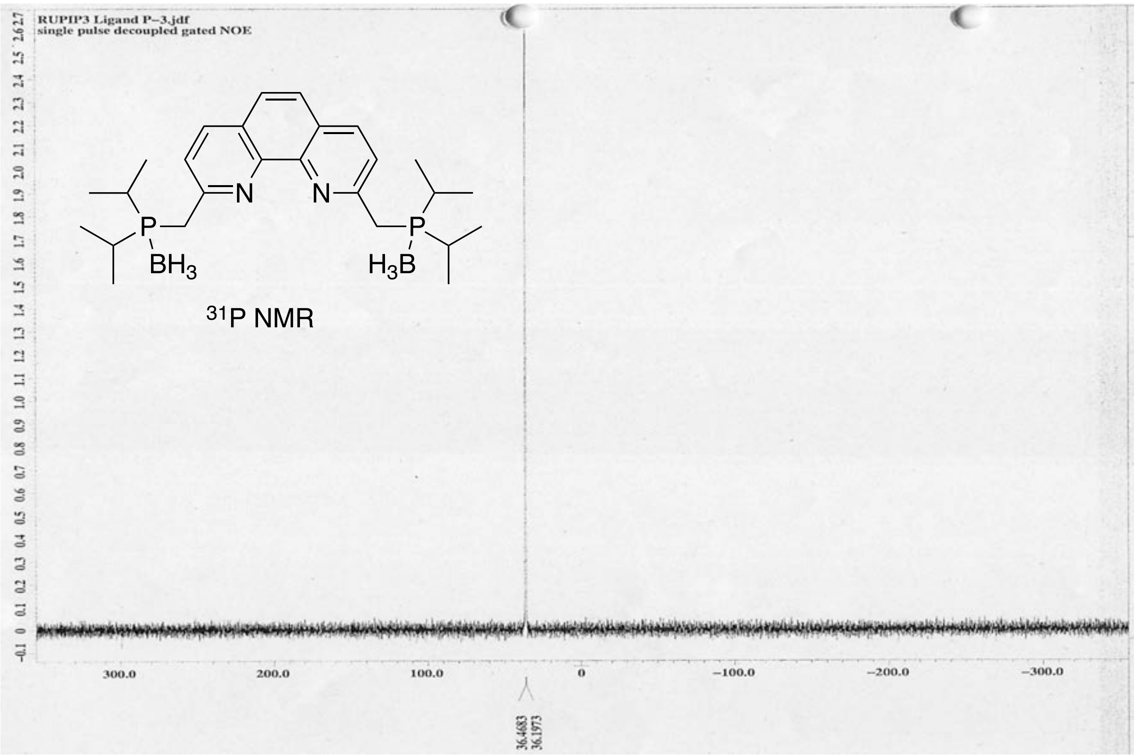

Supplementary Figure S24 | ^{31}P NMR spectrum of 2,9-bis((diisopropylphosphino)methyl)-1,10-phenanthroline−diborane complex

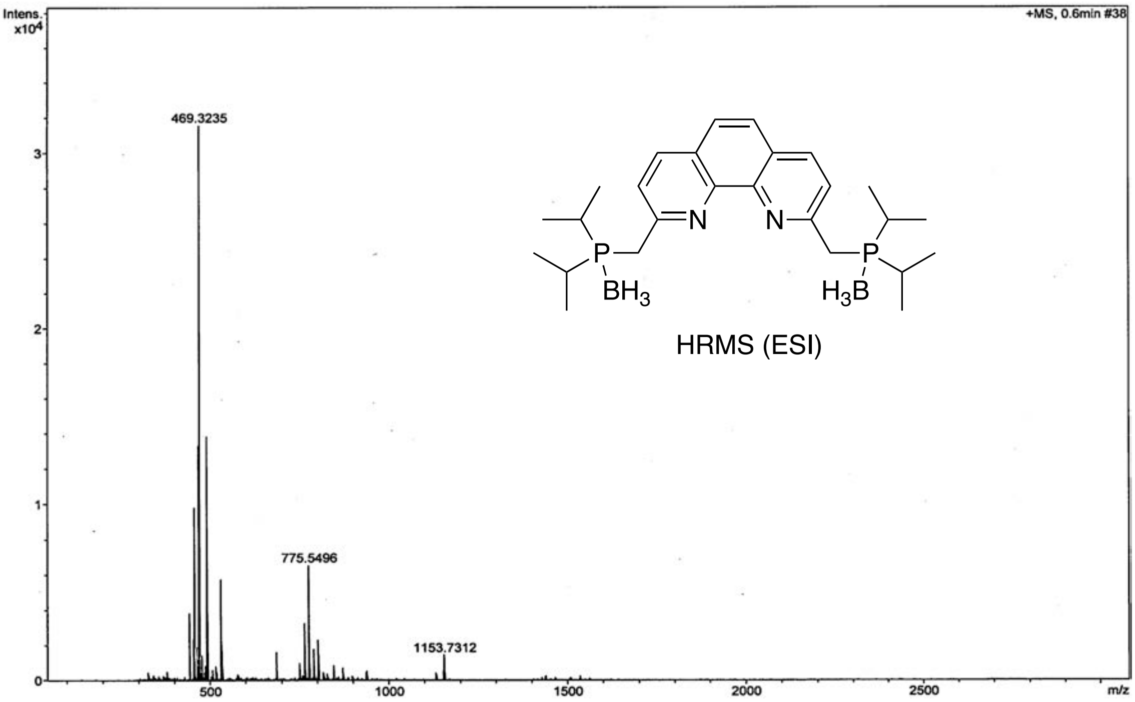

Supplementary Figure S25 | HRMS (ESI) of 2,9-bis((diisopropylphosphino)methyl)-1,10-phenanthroline−diborane complex

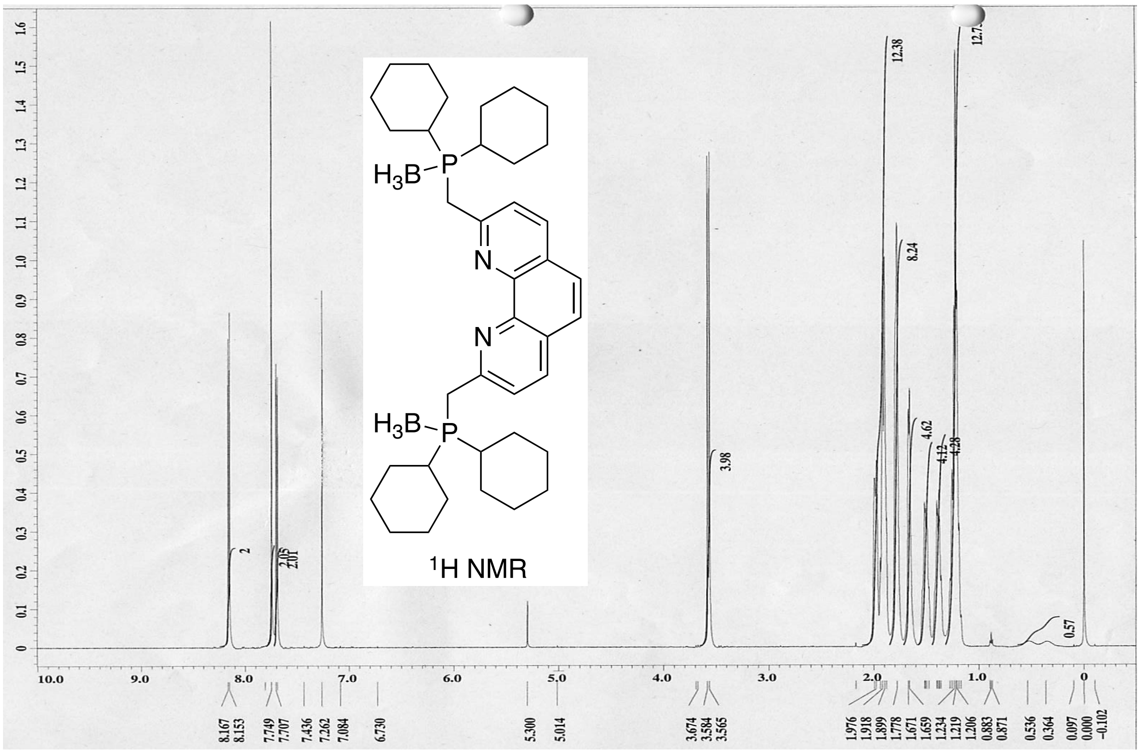

Supplementary Figure S26 | ^{1}H NMR spectrum of 2,9-bis((dicyclohexylphosphino)methyl)-1,10-phenanthroline−diborane complex

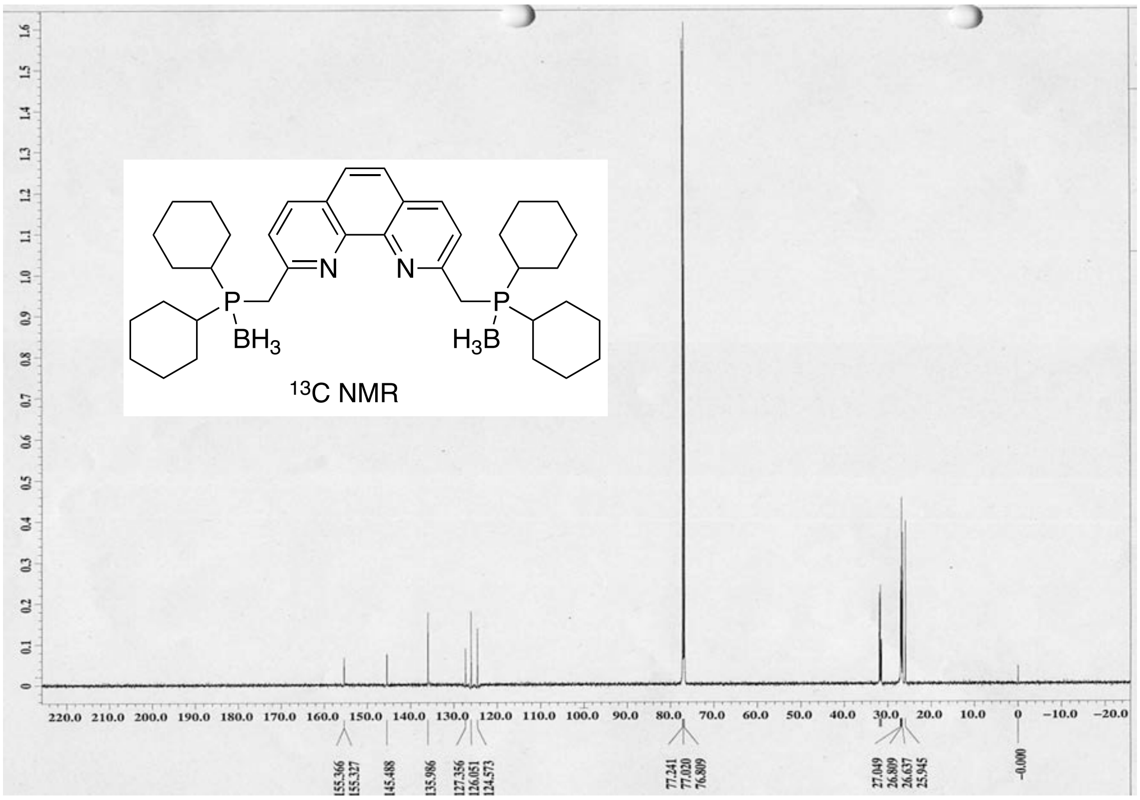

Supplementary Figure S27 | ^{13}C NMR spectrum of 2,9-bis((dicyclohexylphosphino)methyl)-1,10-phenanthroline−diborane complex

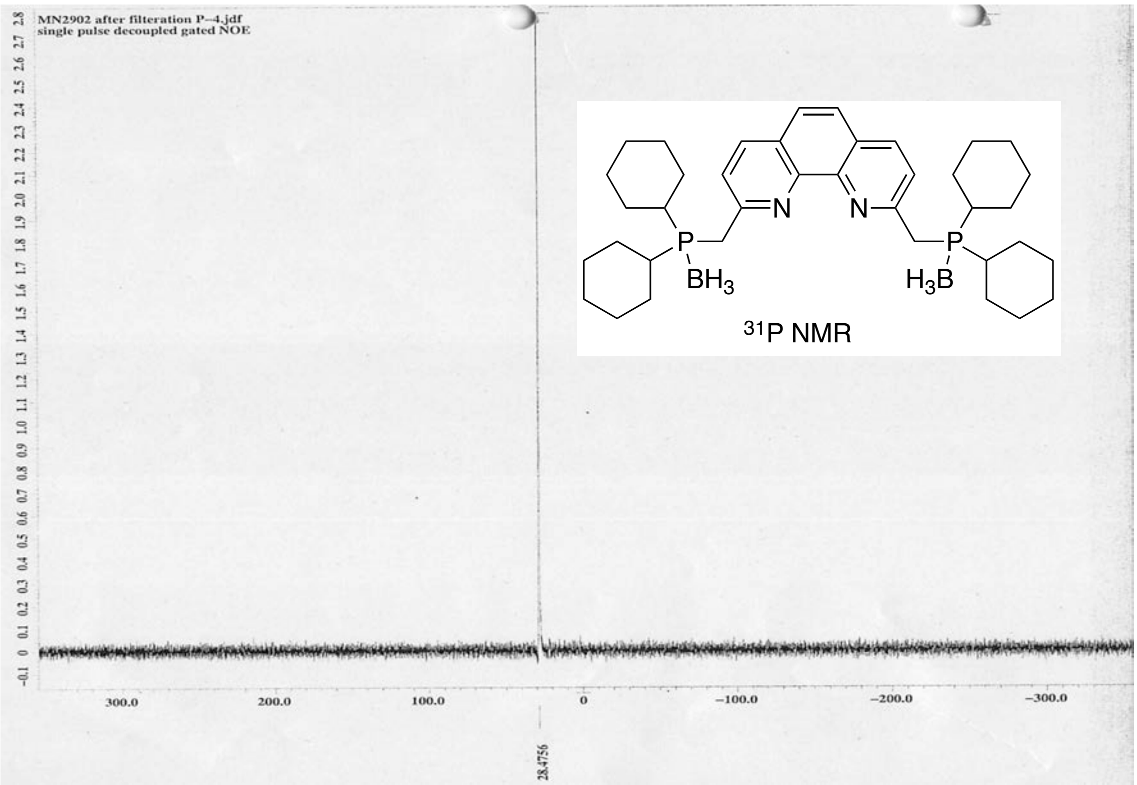

Supplementary Figure S28 | ^{31}P NMR spectrum of 2,9-bis((dicyclohexylphosphino)methyl)-1,10-phenanthroline−diborane complex

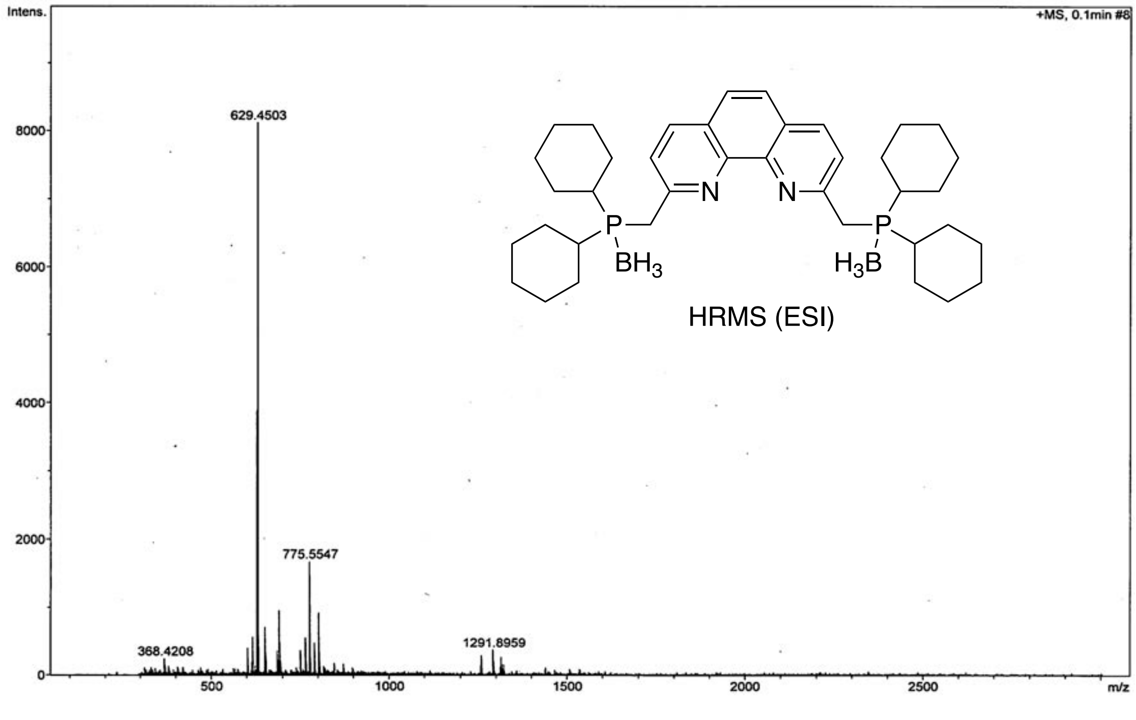

Supplementary Figure S29 | HRMS (ESI) of 2,9-bis((dicyclohexylphosphino)methyl)-1,10-phenanthroline−diborane complex

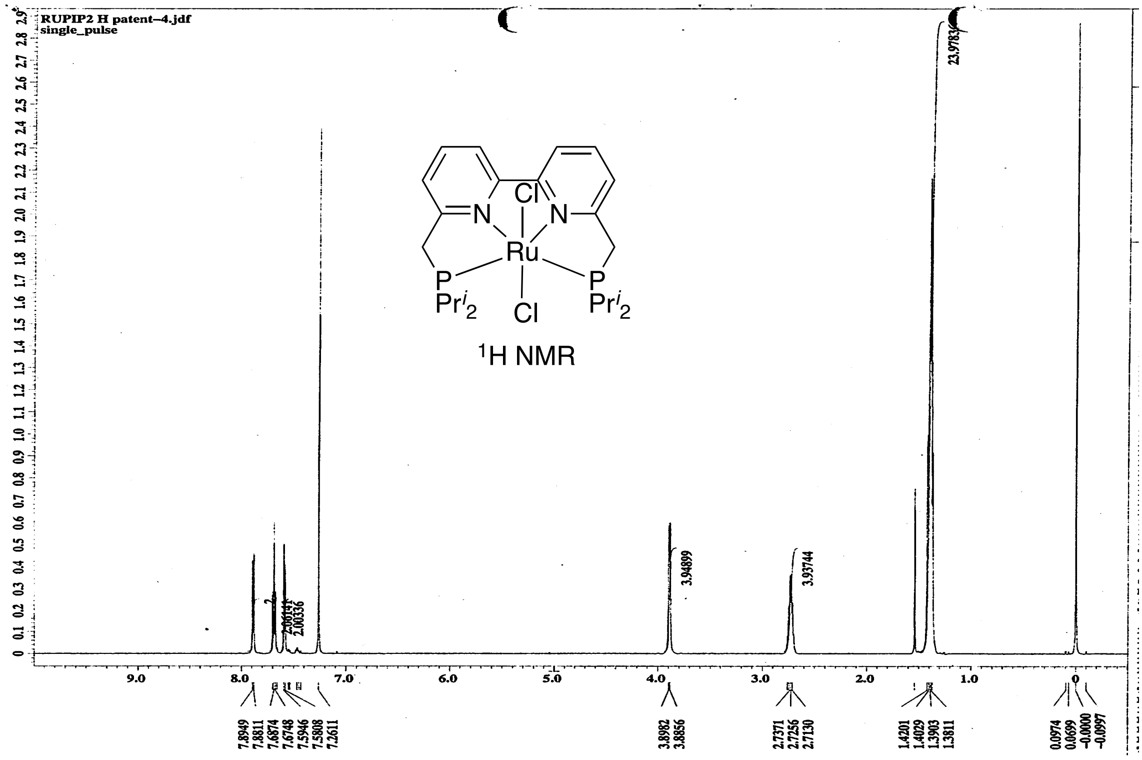

Supplementary Figure S30 | ^{1}H NMR spectrum of RUPIP2 (**1a**), dichloro(6,6'-bis((diisopropylphosphino)methyl)-2,2'-bipyridine)–ruthenium (II)

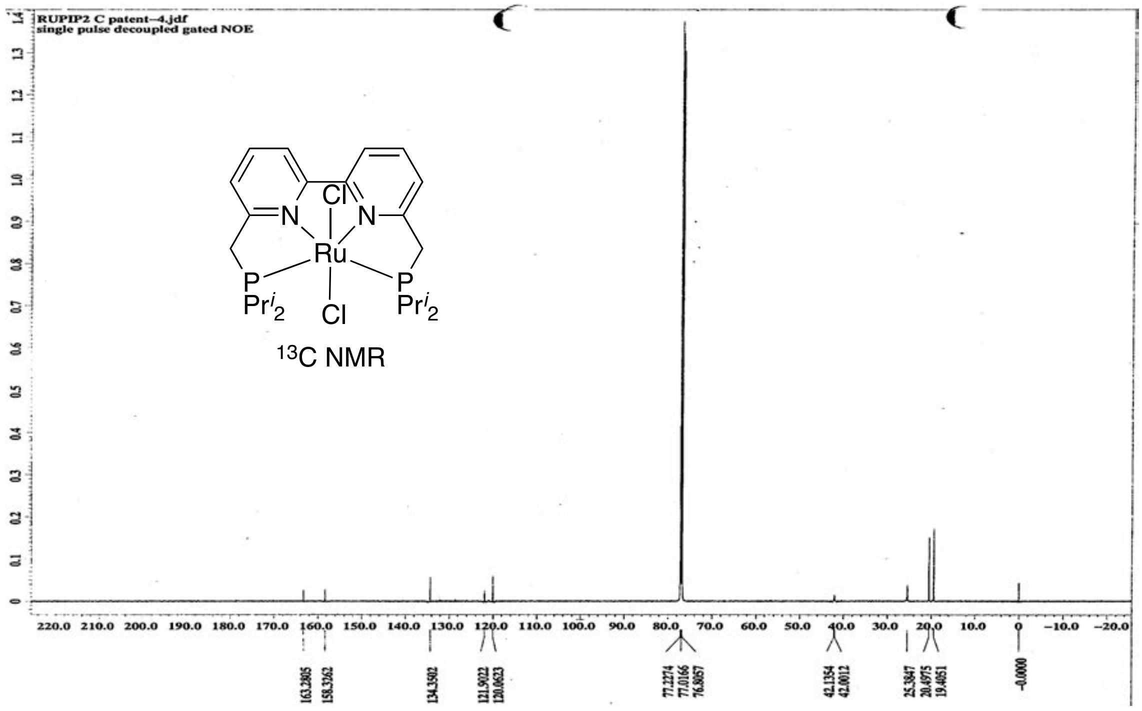

Supplementary Figure S31 | ^{13}C NMR spectrum of RUPIP2 (**1a**), dichloro(6,6'-bis((diisopropylphosphino)methyl)-2,2'-bipyridine)–ruthenium (II)

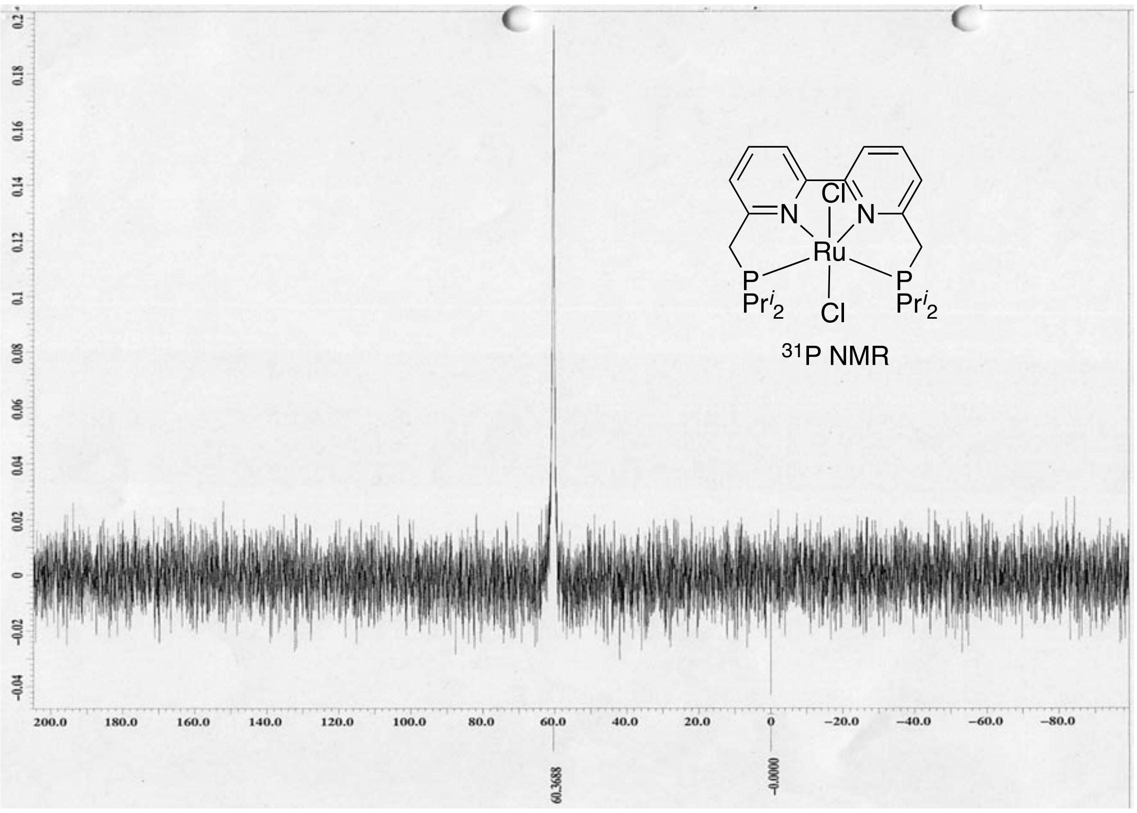

Supplementary Figure S32 | ^{31}P NMR spectrum of RUPIP2 (**1a**), dichloro(6,6'-bis((diisopropylphosphino)methyl)-2,2'-bipyridine)–ruthenium (II)

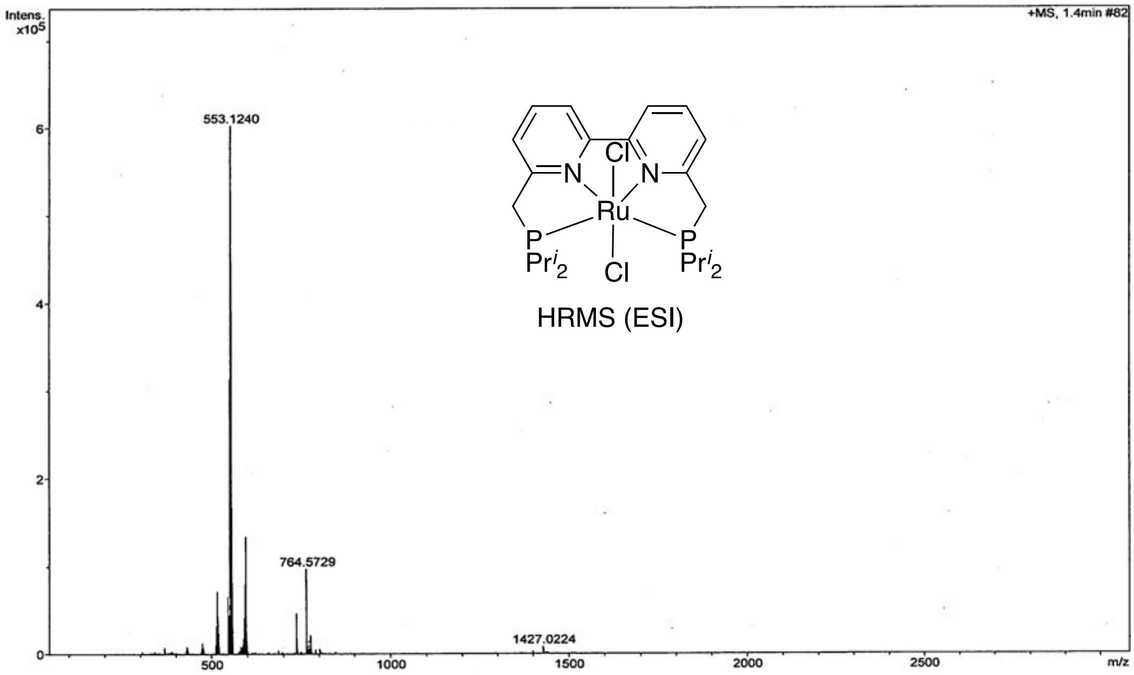

Supplementary Figure S33 | HRMS (ESI) of RUPIP2 (**1a**), dichloro(6,6'-bis((diisopropylphosphino)methyl)-2,2'-bipyridine)–ruthenium (II)

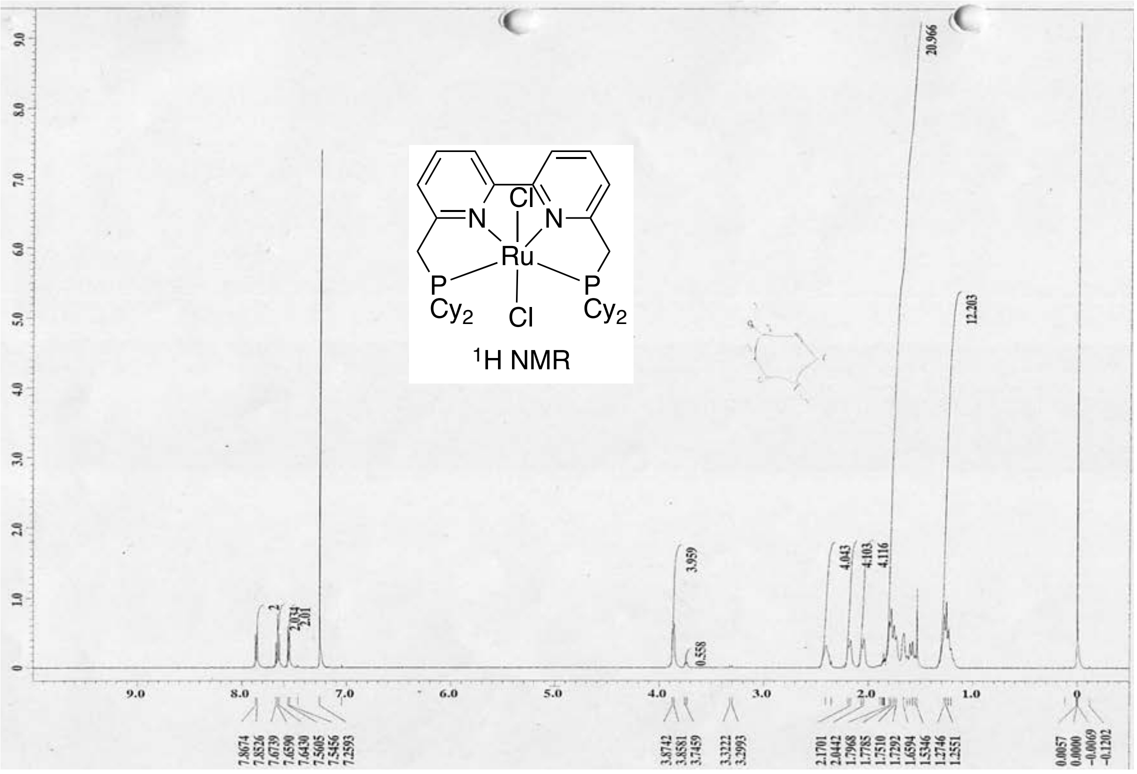

Supplementary Figure S34 | ^{1}H NMR spectrum of RUPCY2 (**1b**), dichloro(6,6'-bis((dicyclohexylphosphino)methyl)-2,2'-bipyridine)–ruthenium (II)

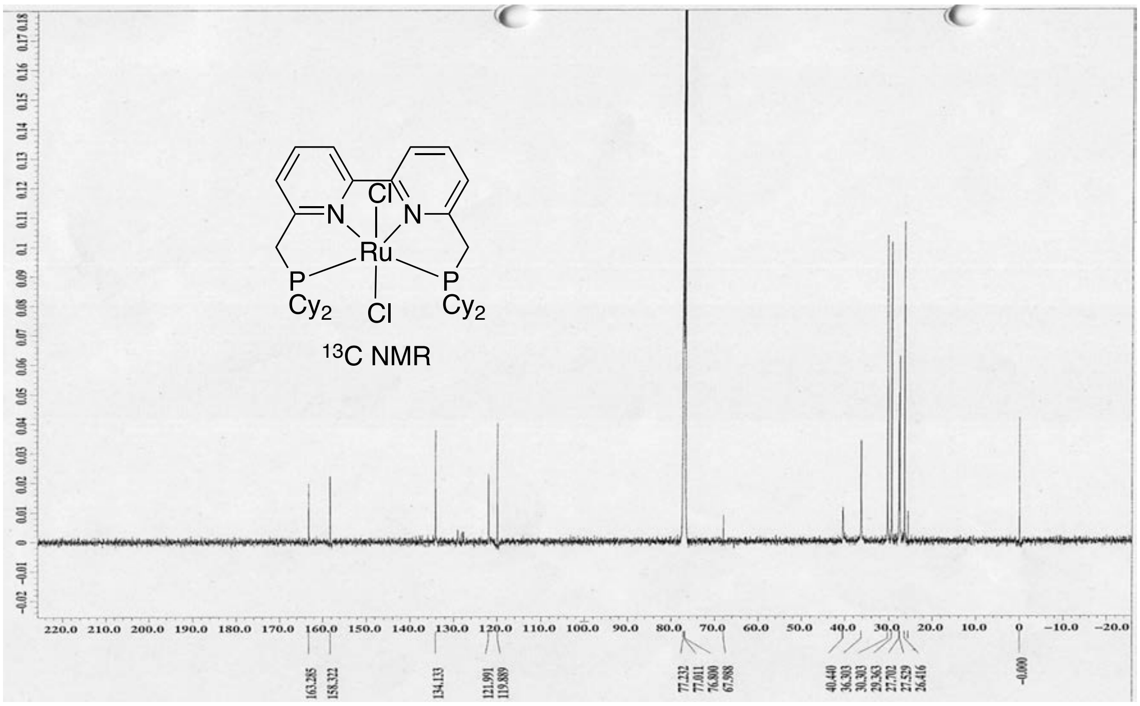

Supplementary Figure S35 | ^{13}C NMR spectrum of RUPCY2 (**1b**), dichloro(6,6'-bis((dicyclohexylphosphino)methyl)-2,2'-bipyridine)–ruthenium (II)

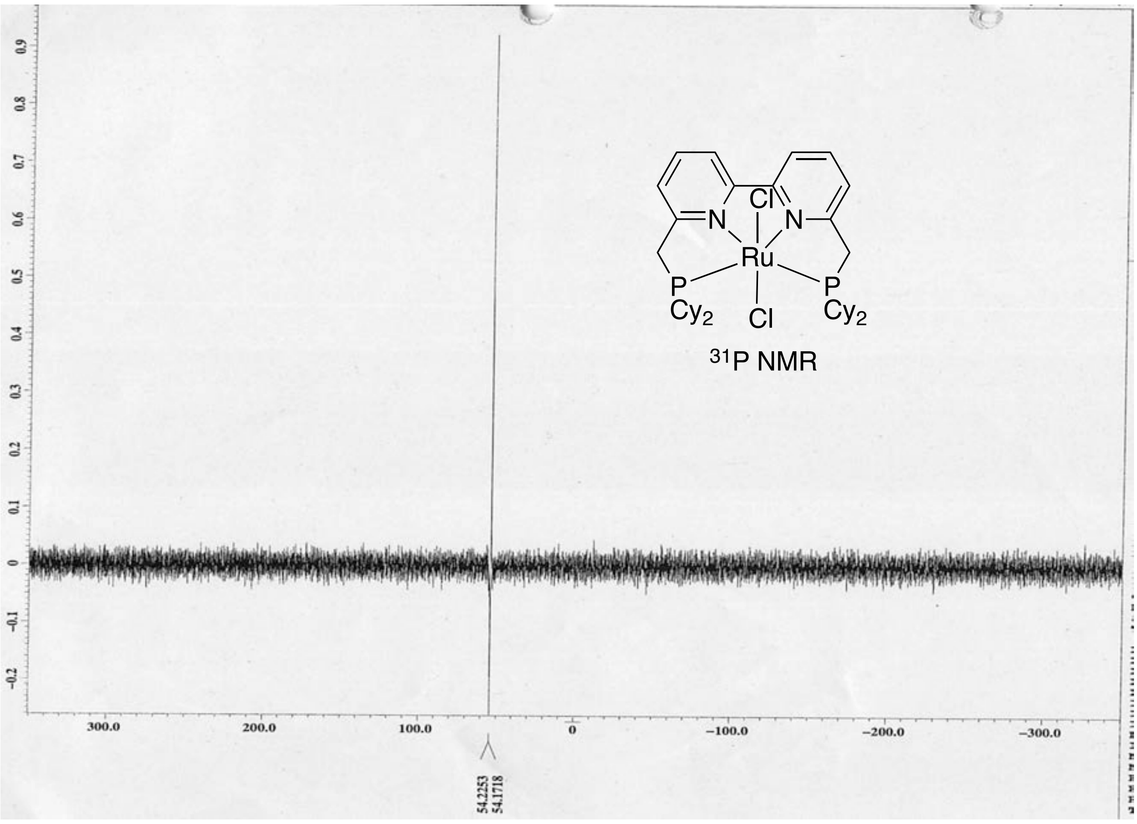

Supplementary Figure S36 | ^{31}P NMR spectrum of RUPCY2 (**1b**), dichloro(6,6'-bis((dicyclohexylphosphino)methyl)-2,2'-bipyridine)–ruthenium (II)

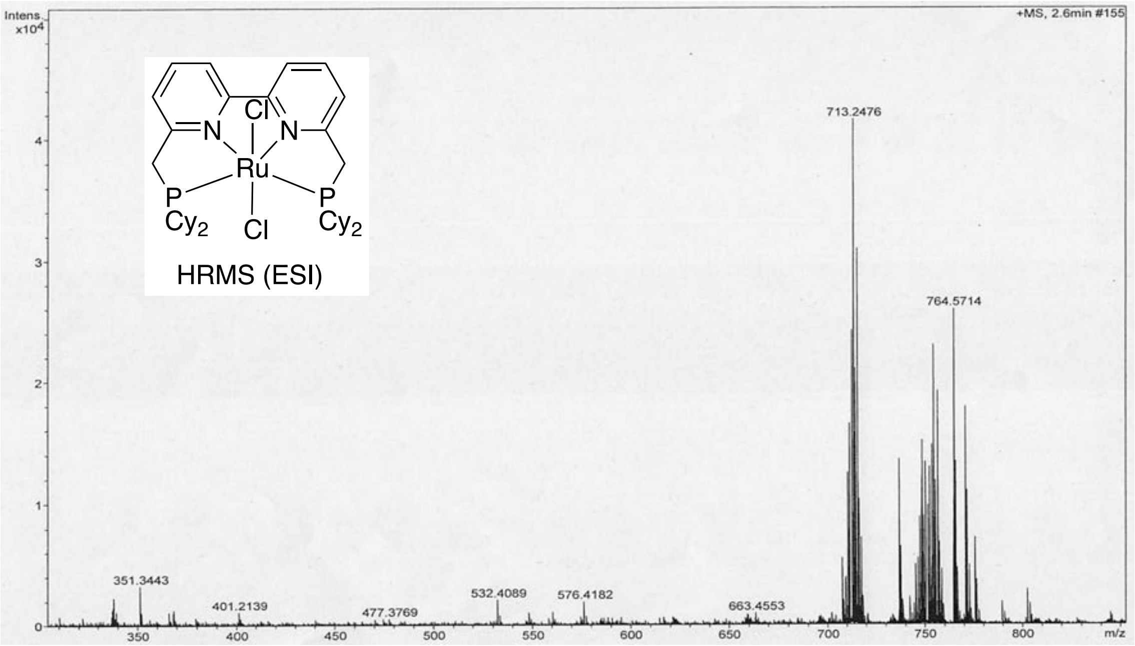

Supplementary Figure S37 | HRMS (ESI) of RUPCY2 (**1b**), dichloro(6,6'-bis((dicyclohexylphosphino)methyl)-2,2'-bipyridine)–ruthenium (II)

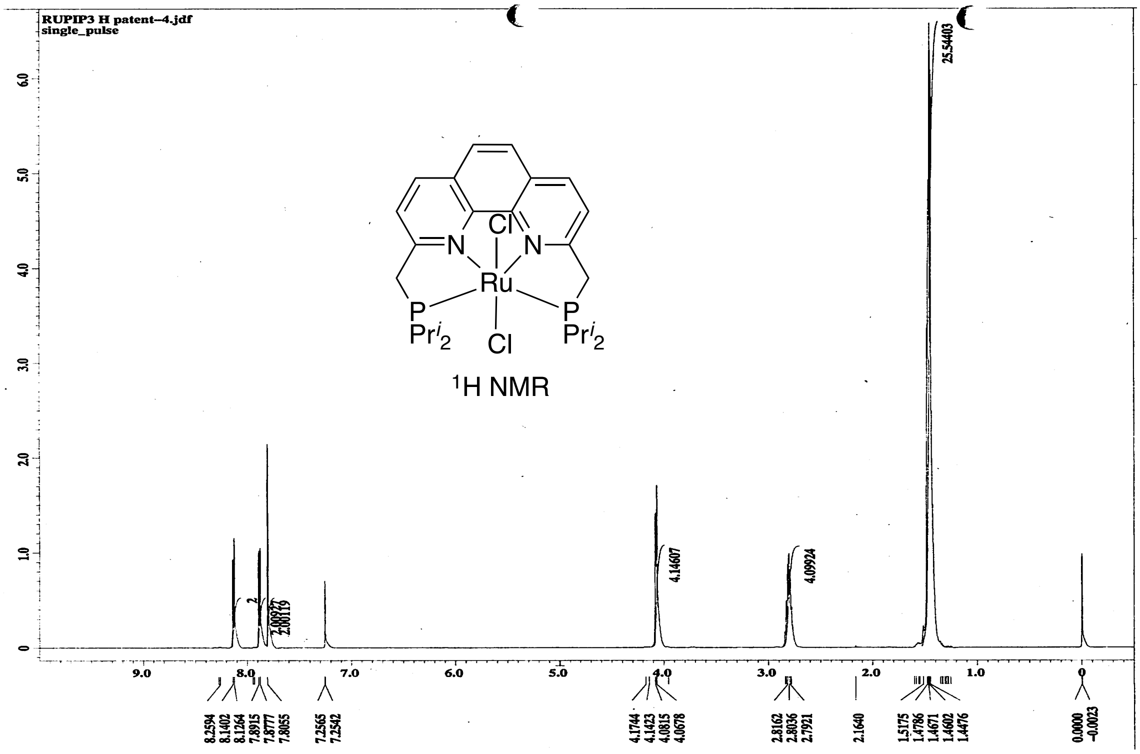

Supplementary Figure S38 | ^{1}H NMR spectrum of RUPIP3 (**1d**), dichloro(2,9-bis((diisopropylphosphino)methyl)-1,10-phenanthroline)–ruthenium (II)

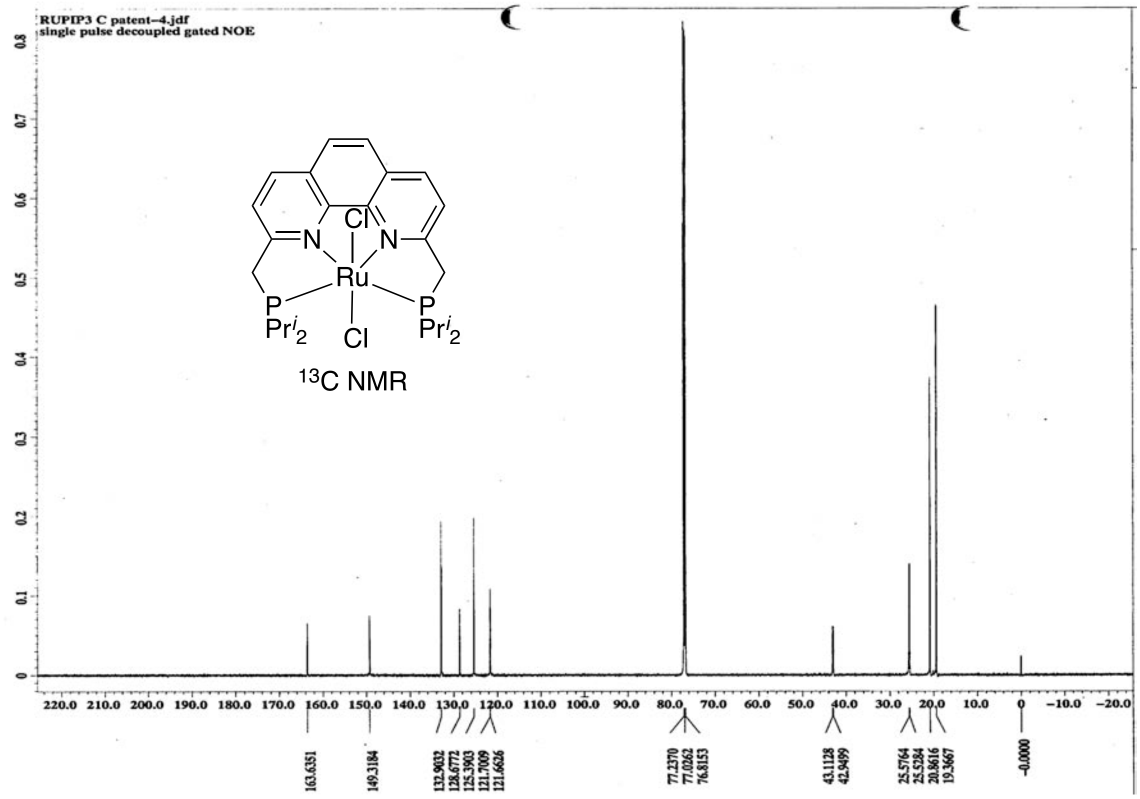

Supplementary Figure S39 | ^{13}C NMR spectrum of RUPIP3 (**1d**), dichloro(2,9-bis((diisopropylphosphino)methyl)-1,10-phenanthroline)–ruthenium (II)

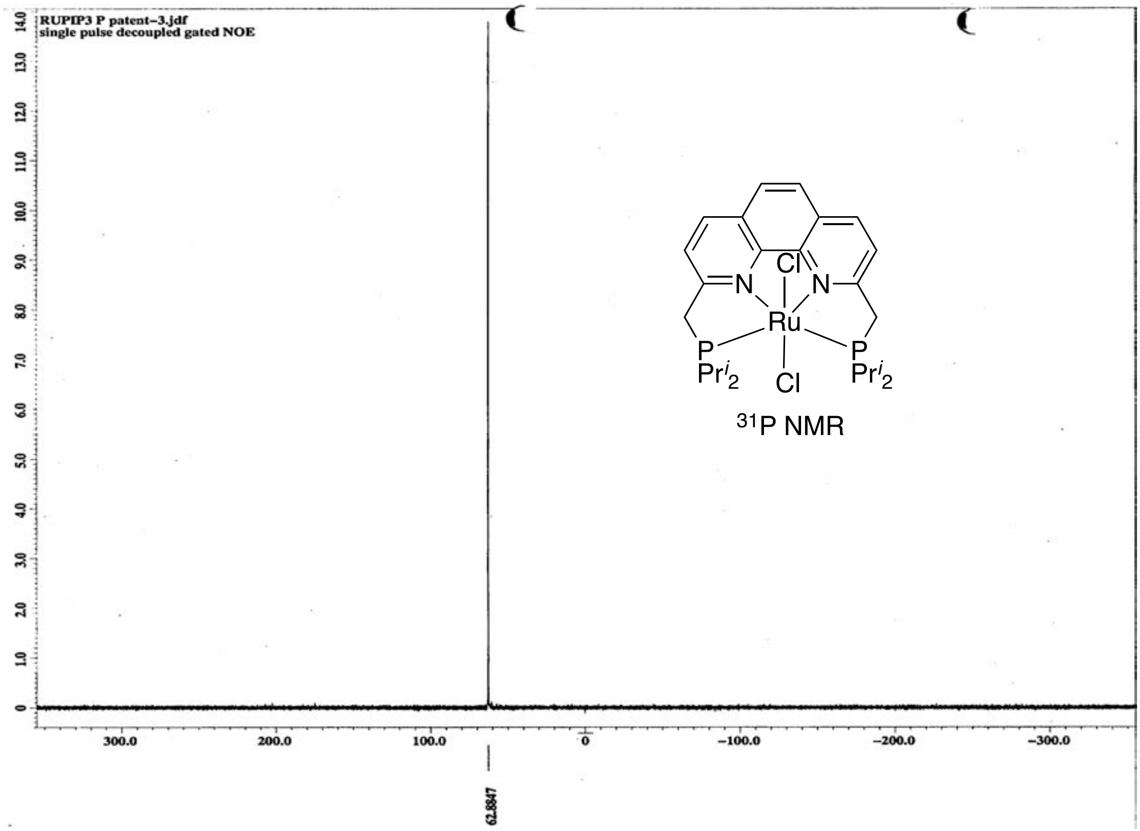

Supplementary Figure S40 | ^{31}P NMR spectrum of RUPIP3 (**1d**), dichloro(2,9-bis((diisopropylphosphino)methyl)-1,10-phenanthroline)–ruthenium (II)

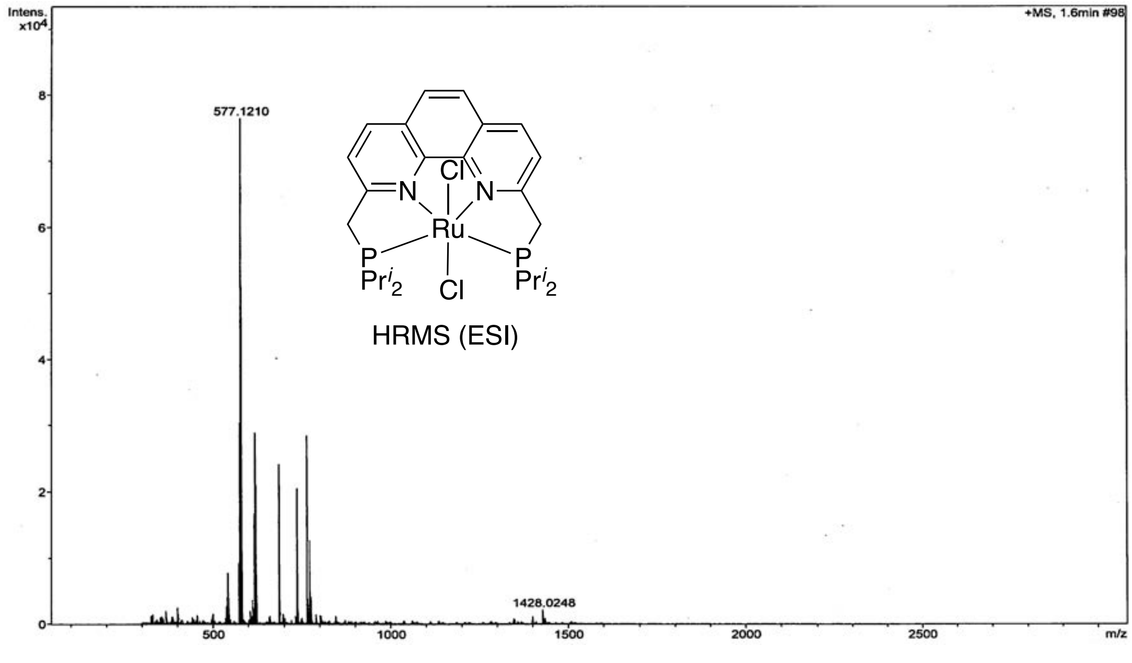

Supplementary Figure S41 | HRMS (ESI) of RUPIP3 (**1d**), dichloro(2,9-bis((diisopropylphosphino)methyl)-1,10-phenanthroline)–ruthenium (II)

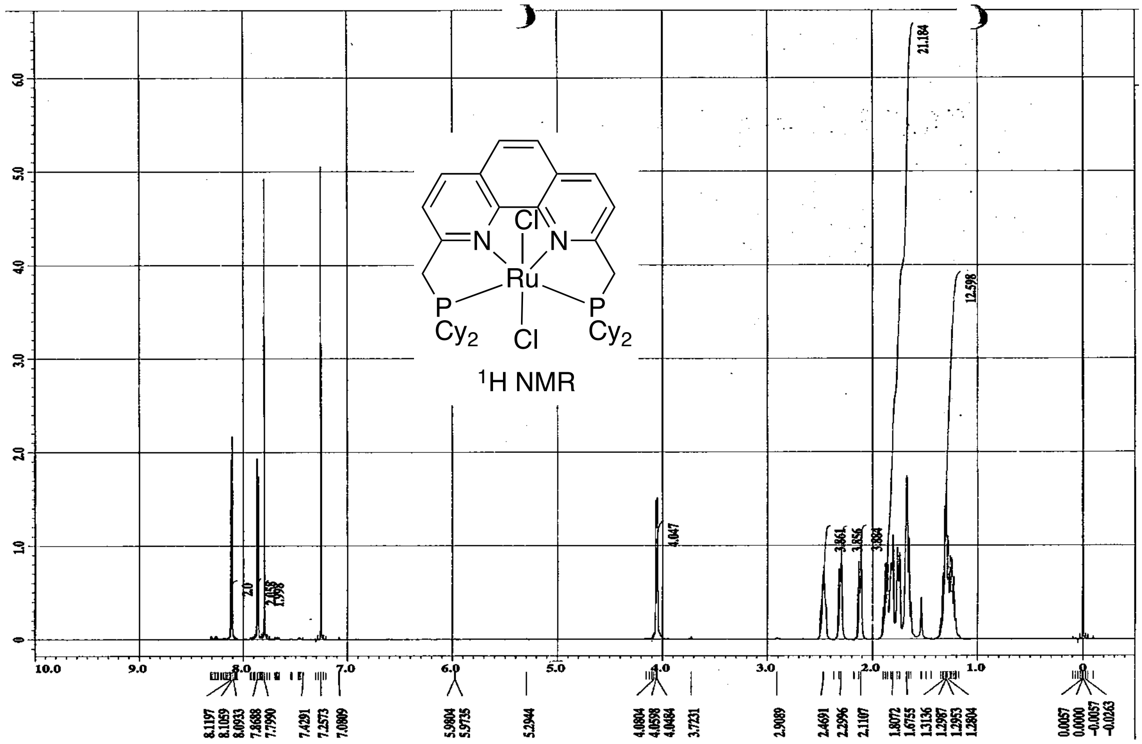

Supplementary Figure S42 | ^{1}H NMR spectrum of RUPCY3 (**1e**), dichloro(2,9-bis((dicyclohexylphosphino)methyl)-1,10-phenanthroline)–ruthenium (II)

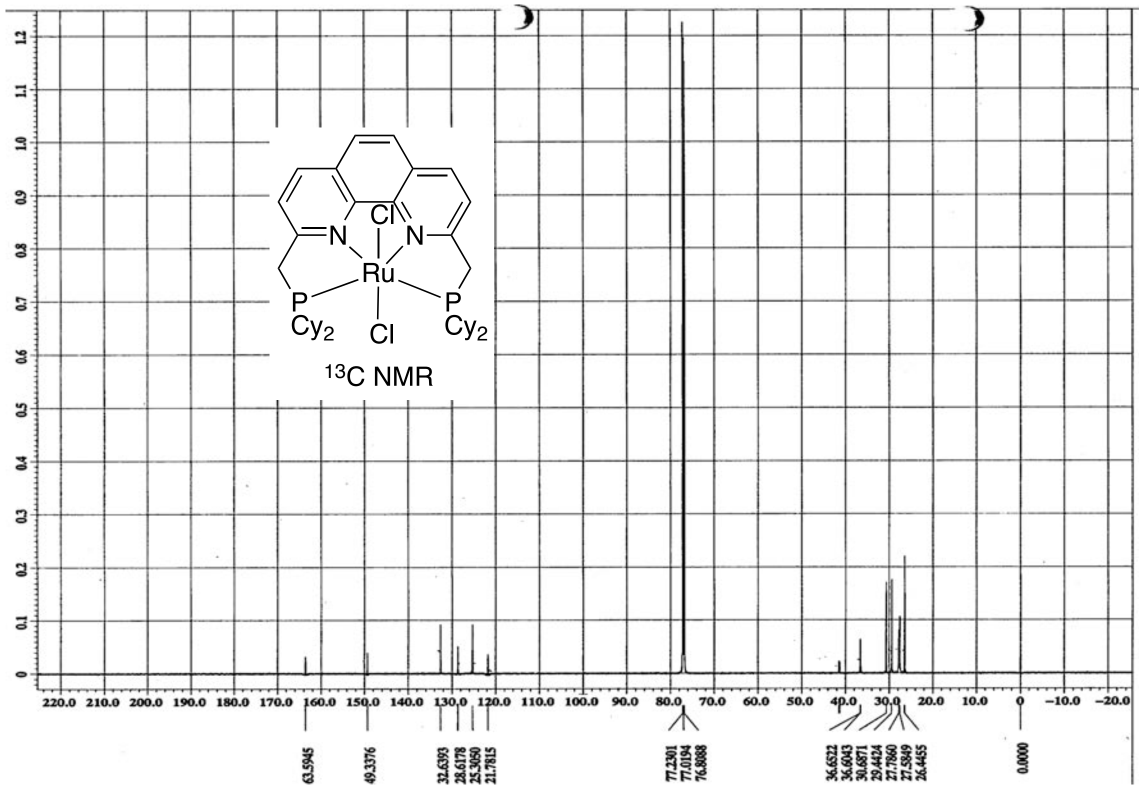

Supplementary Figure S43 | ^{13}C NMR spectrum of RUPCY3 (**1e**), dichloro(2,9-bis((dicyclohexylphosphino)methyl)-1,10-phenanthroline)–ruthenium (II)

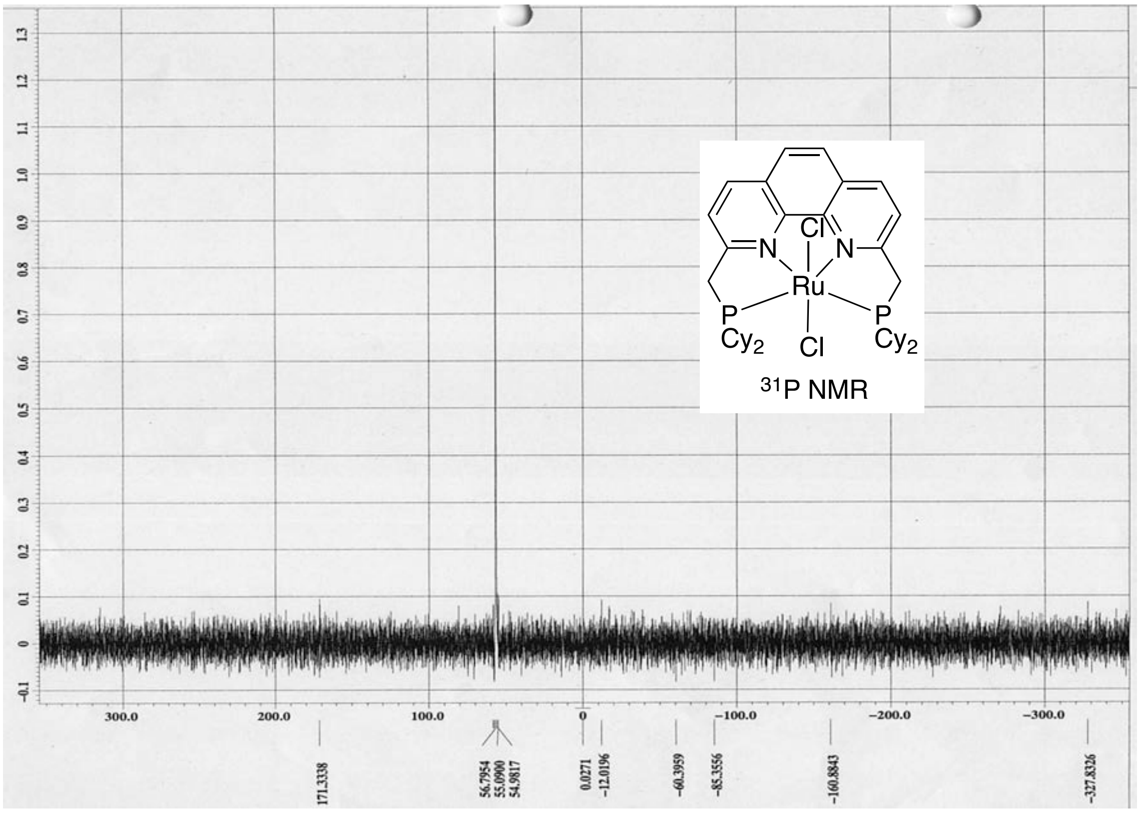

Supplementary Figure S44 | ^{31}P NMR spectrum of RUPCY3 (**1e**), dichloro(2,9-bis((dicyclohexylphosphino)methyl)-1,10-phenanthroline)–ruthenium (II)

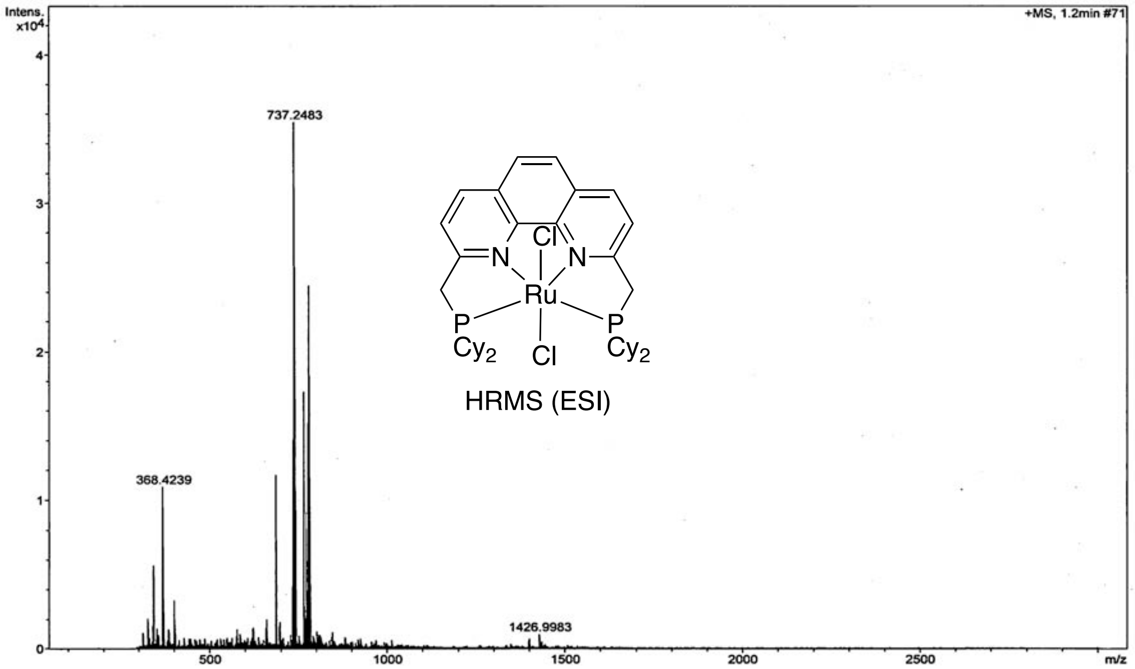

Supplementary Figure S45 | HRMS (ESI) of RUPCY3 (**1e**), dichloro(2,9-bis((dicyclohexylphosphino)methyl)-1,10-phenanthroline)–ruthenium (II)

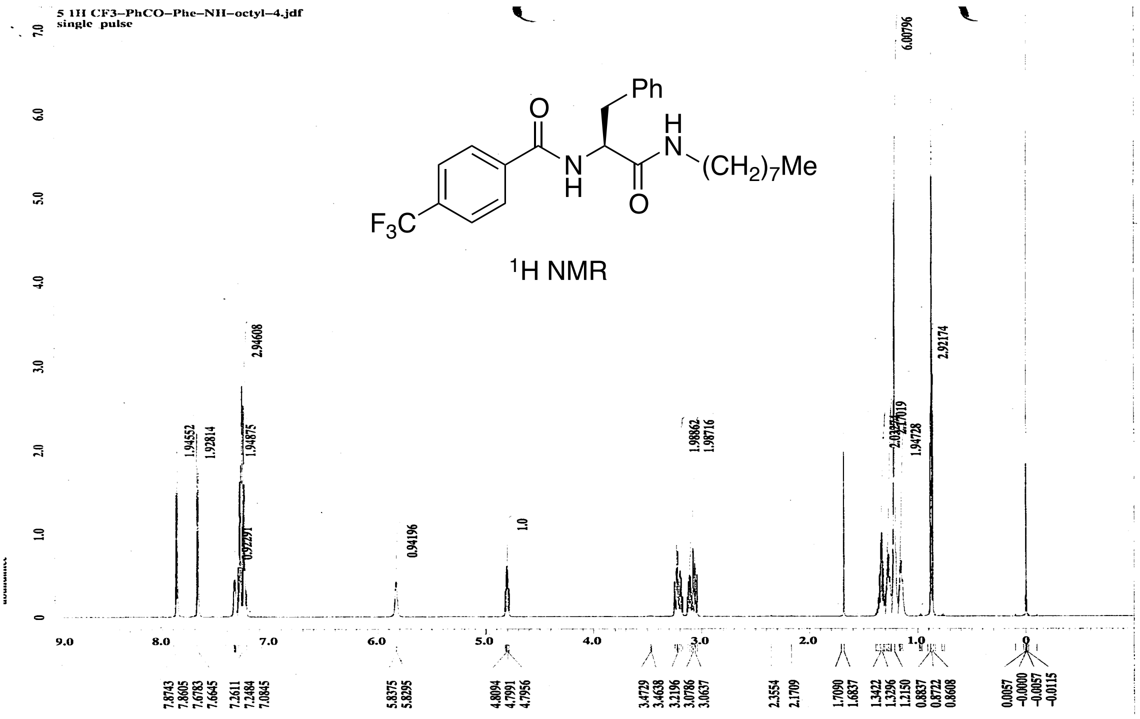

Supplementary Figure S46 | ^{1}H NMR spectrum of (*S*)-*N*-(1-(octylamino)-1-oxo-3-phenylpropan-2-yl)-4-(trifluoromethyl)benzamide (**3s**)

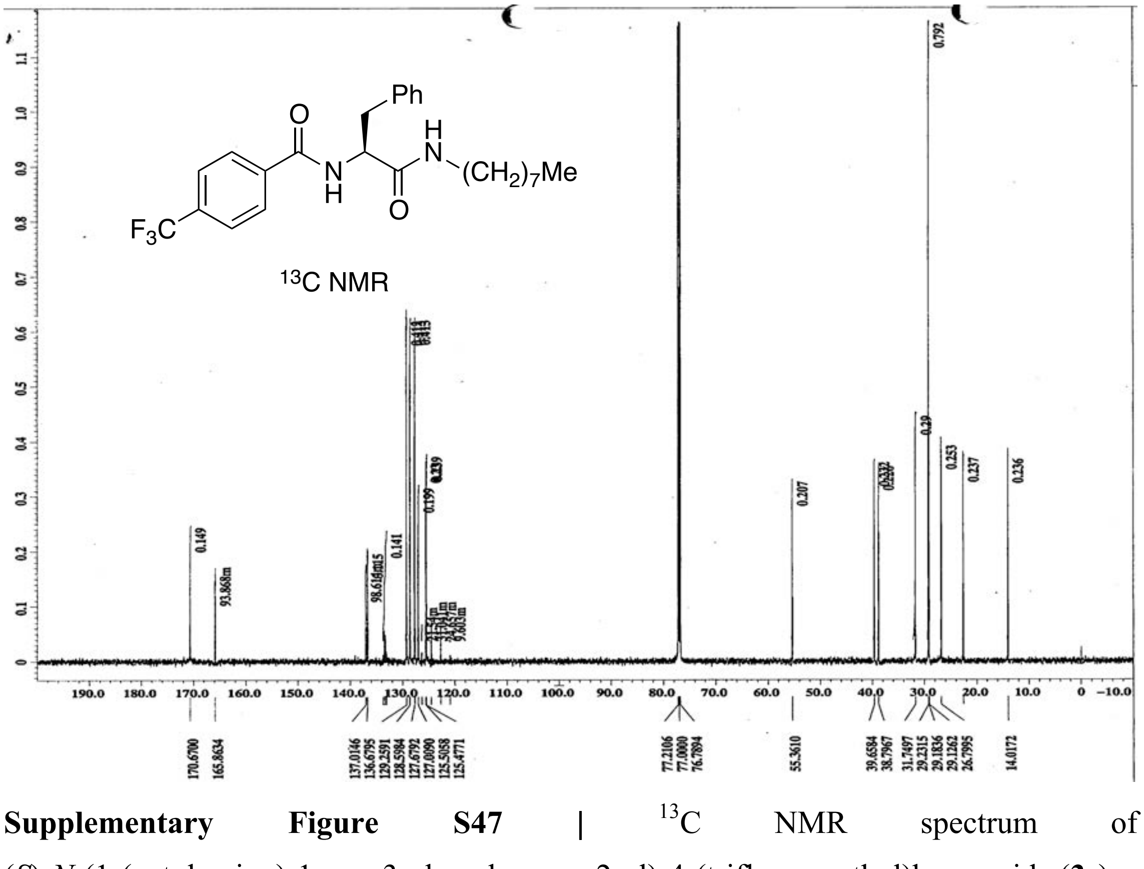

Supplementary Figure S47 | ^{13}C NMR spectrum of (*S*)-*N*-(1-(octylamino)-1-oxo-3-phenylpropan-2-yl)-4-(trifluoromethyl)benzamide (**3s**)

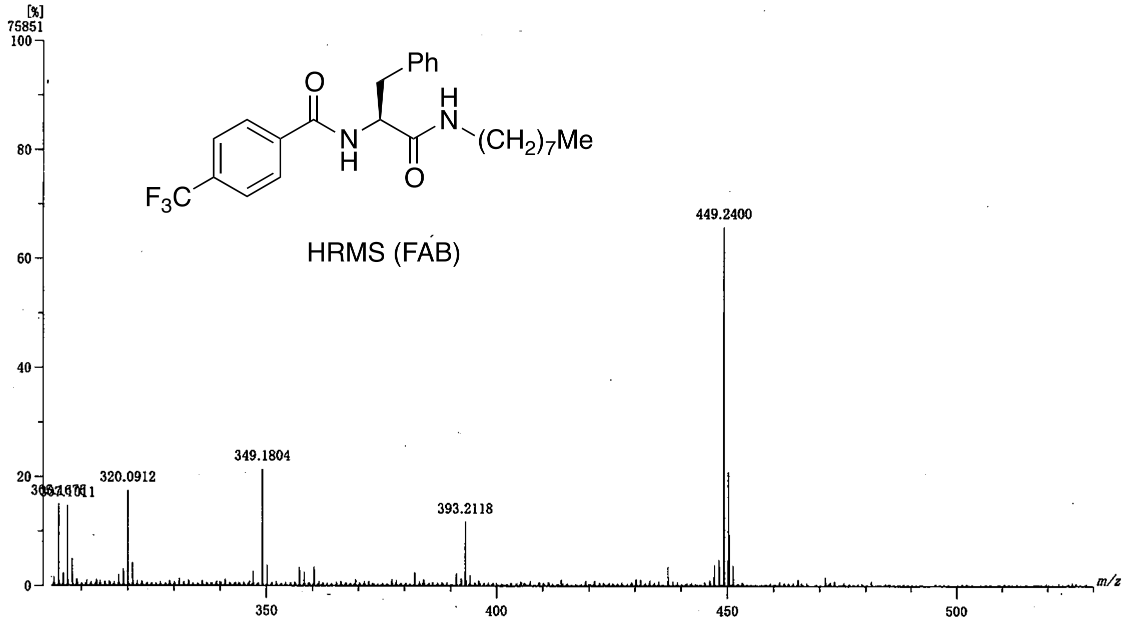

Supplementary Figure S48 | HRMS (FAB) of (*S*)-*N*-(1-(octylamino)-1-oxo-3-phenylpropan-2-yl)-4-(trifluoromethyl)benzamide (**3s**)

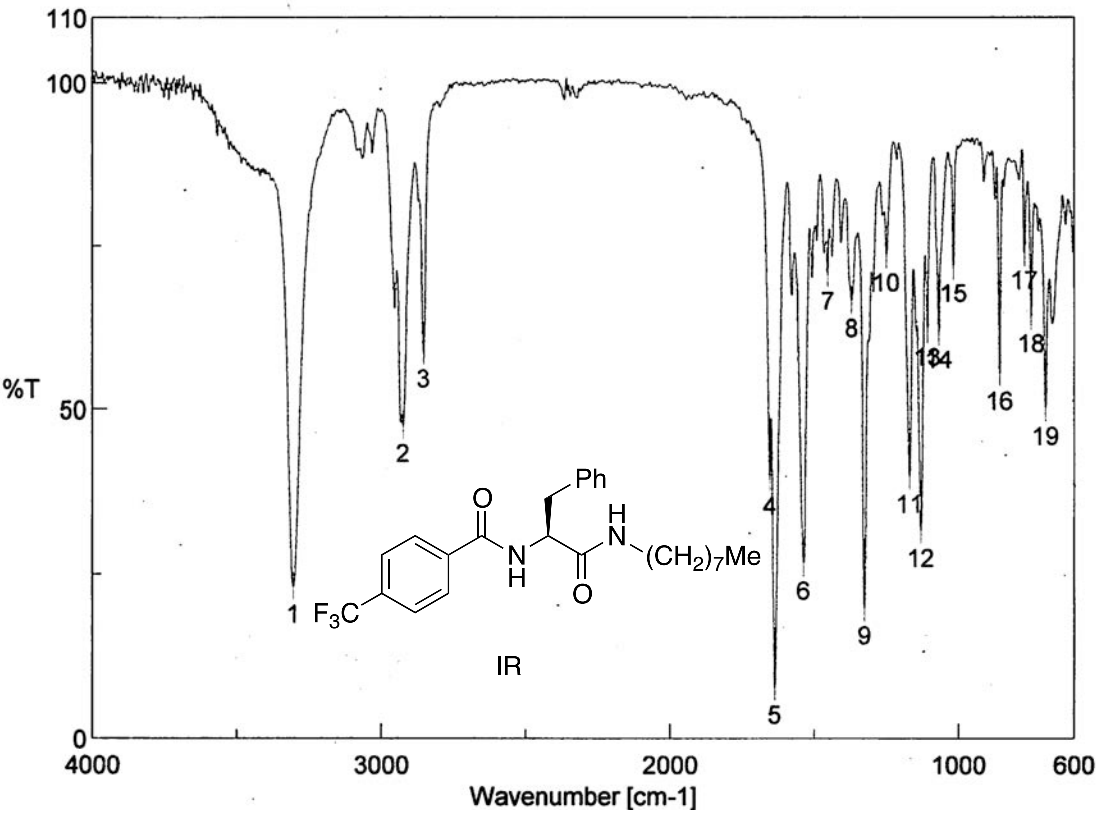

Supplementary Figure S49 | IR spectrum of (*S*)-*N*-(1-(octylamino)-1-oxo-3-phenylpropan-2-yl)-4-(trifluoromethyl)benzamide (**3s**)

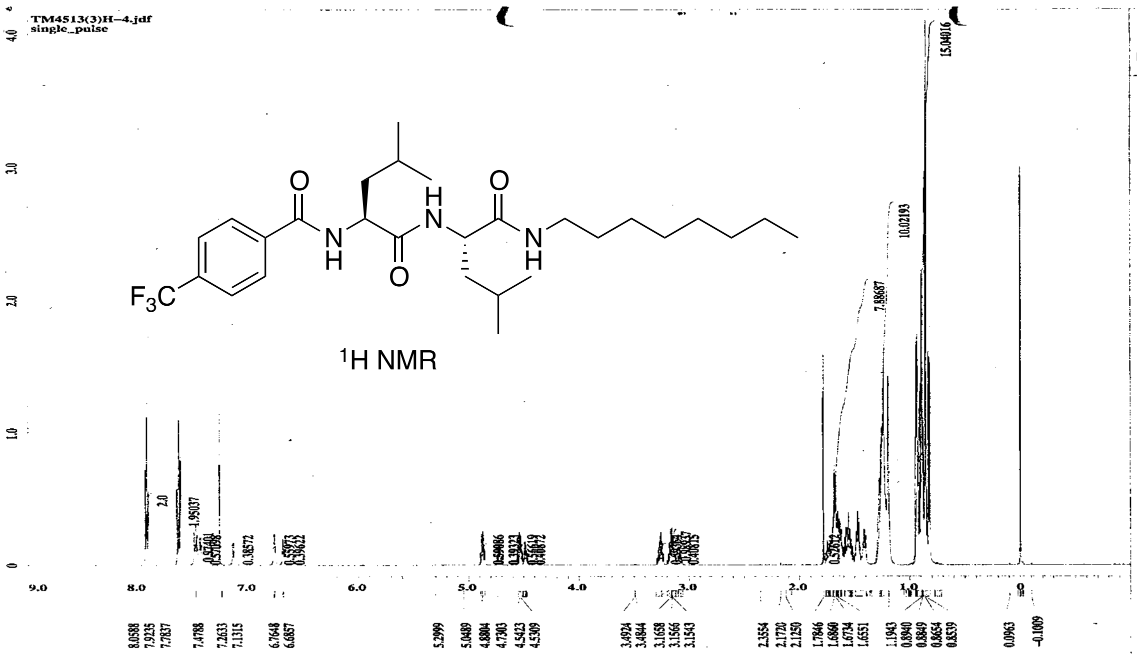

Supplementary Figure S50 | ^{1}H NMR spectrum of *N*-((*S*)-4-methyl-1-(((*S*)-4-methyl-1-(octylamino)-1-oxopentan-2-yl)amino)-1-oxopentan-2-yl)-4-(trifluoromethyl)benzamide (**3t**)

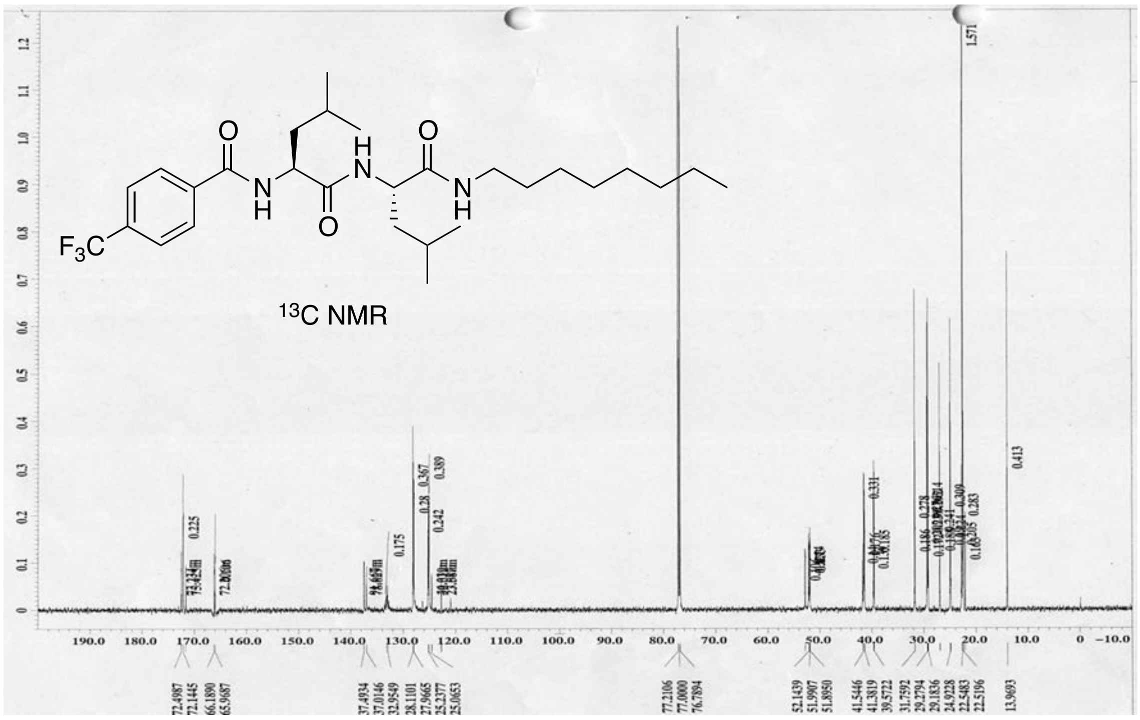

Supplementary Figure S51 | ^{13}C NMR spectrum of *N*-((*S*)-4-methyl-1-(((*S*)-4-methyl-1-(octylamino)-1-oxopentan-2-yl)amino)-1-oxopentan-2-yl)-4-(trifluoromethyl)benzamide (**3t**)

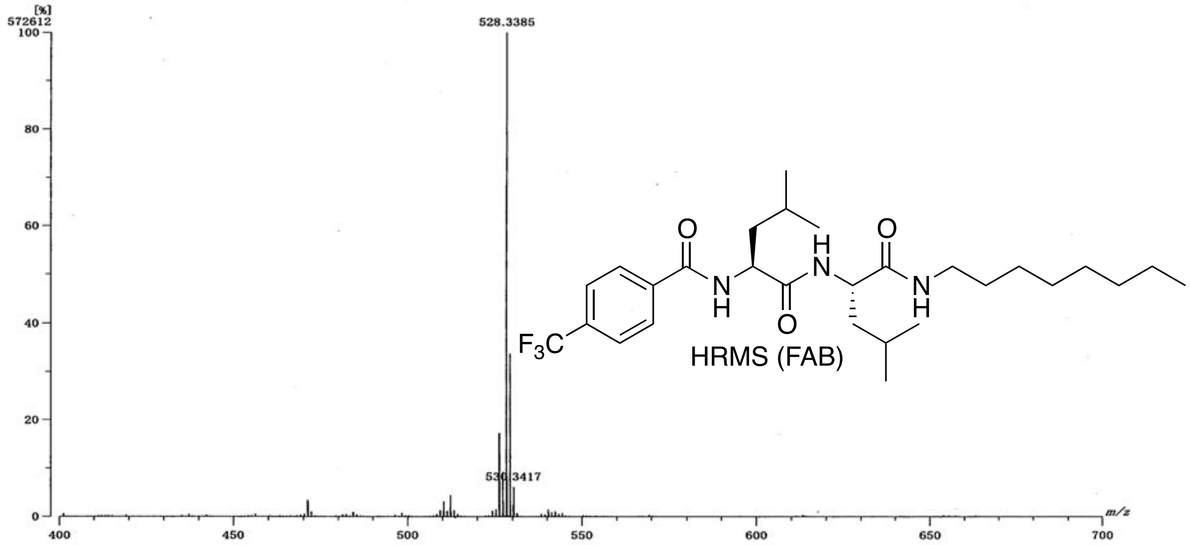

Supplementary Figure S52 | HRMS (FAB) of *N*-((*S*)-4-methyl-1-(((*S*)-4-methyl-1-(octylamino)-1-oxopentan-2-yl)amino)-1-oxopentan-2-yl)-4-(trifluoromethyl)benzamide (**3t**)

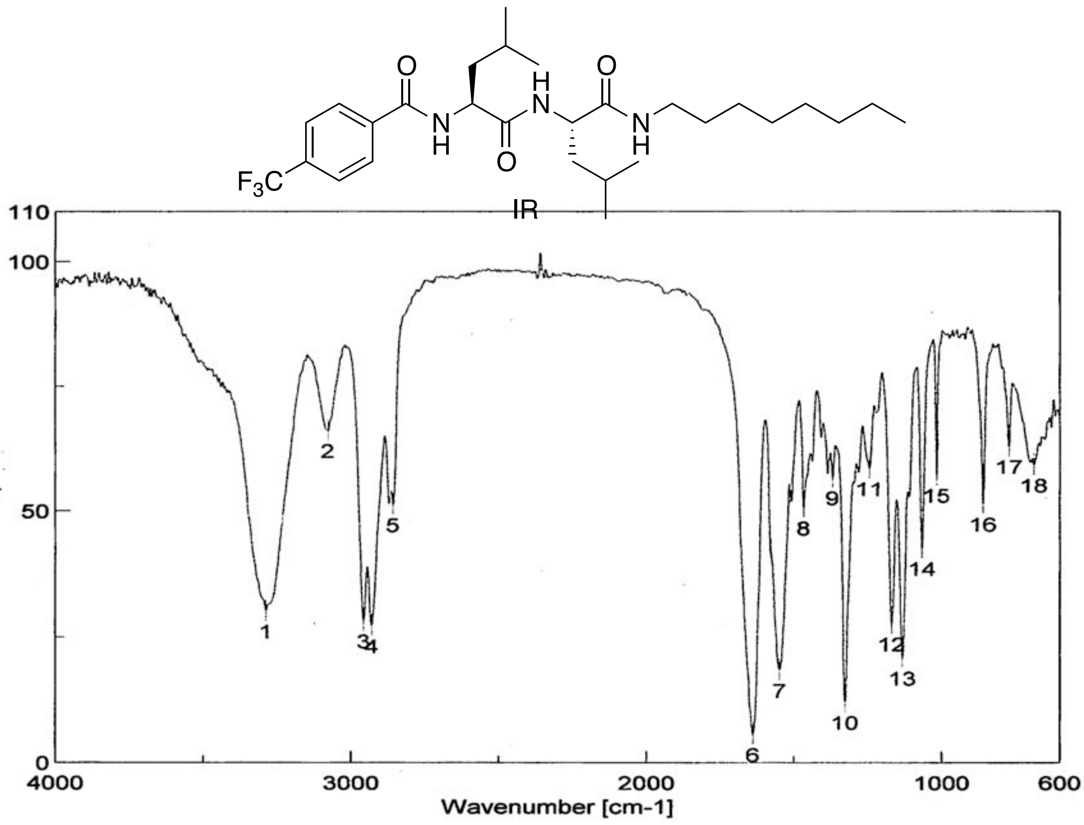

Supplementary Figure S53 | IR spectrum of *N*-((*S*)-4-methyl-1-(((*S*)-4-methyl-1-(octylamino)-1-oxopentan-2-yl)amino)-1-oxopentan-2-yl)-4-(trifluoromethyl)benzamide (**3t**)

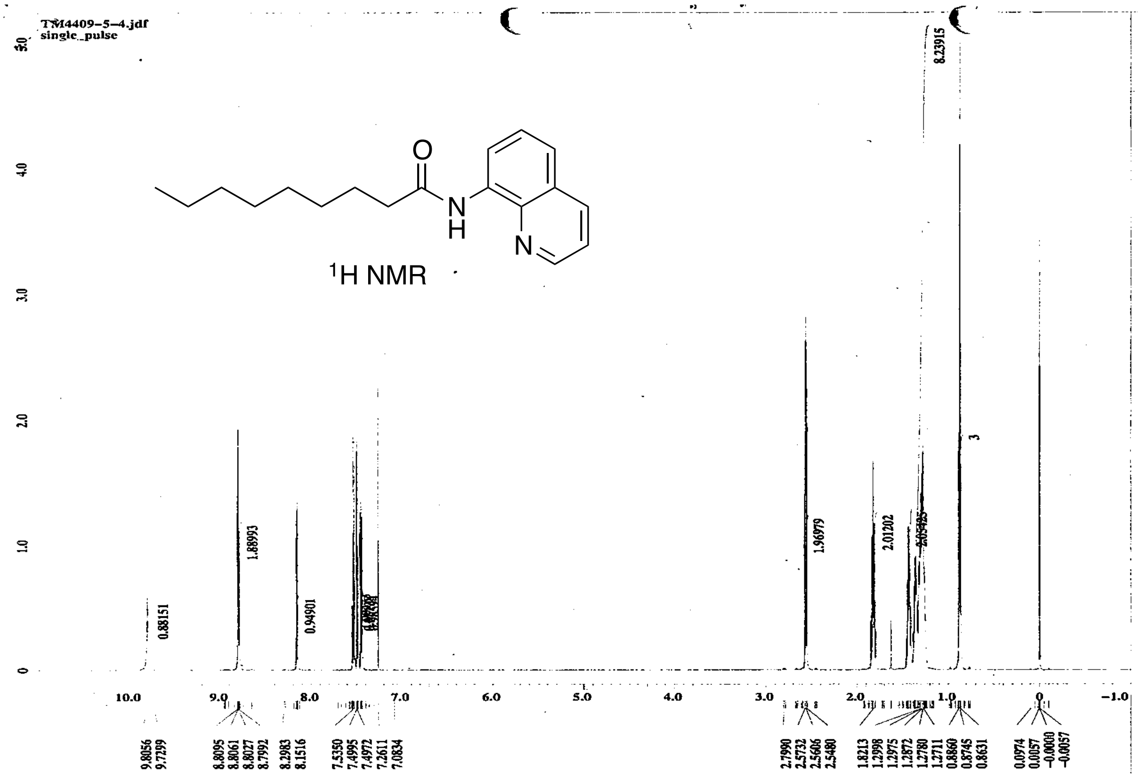

Supplementary Figure S54 | ^{1}H NMR spectrum of *N*-(quinolin-8-yl)nonanamide (**3u**)

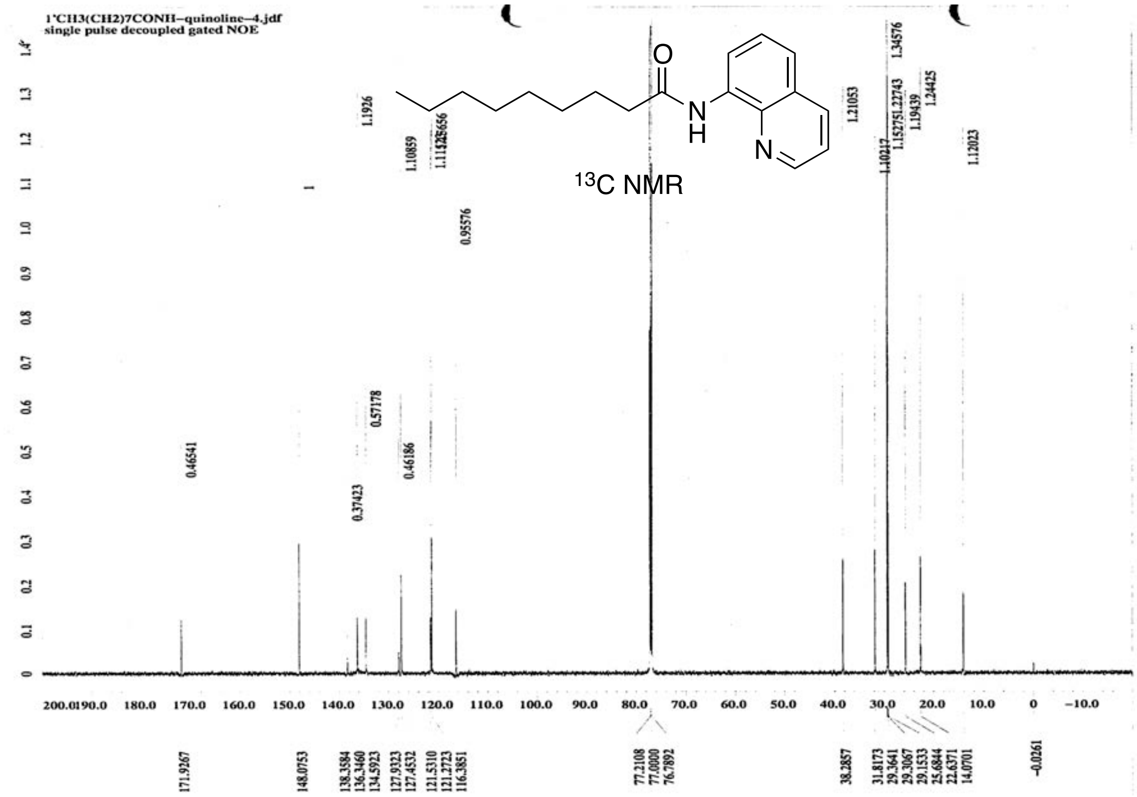

Supplementary Figure S55 | ^{13}C NMR spectrum of *N*-(quinolin-8-yl)nonanamide (**3u**)

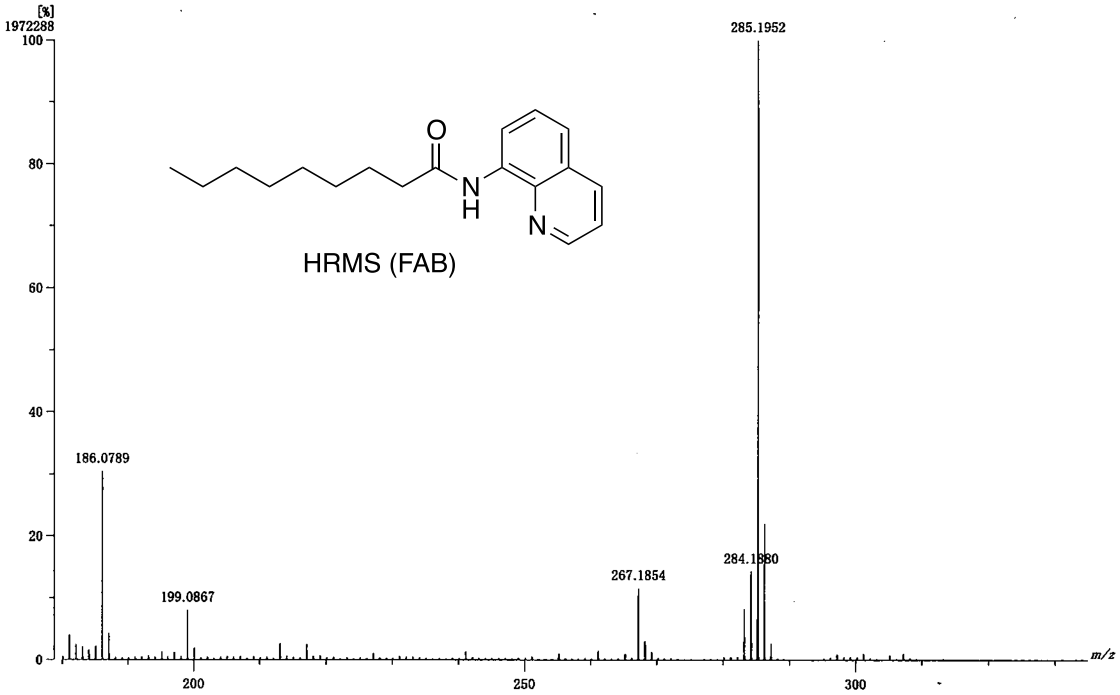

Supplementary Figure S56 | HRMS (FAB) of *N*-(quinolin-8-yl)nonanamide (**3u**)

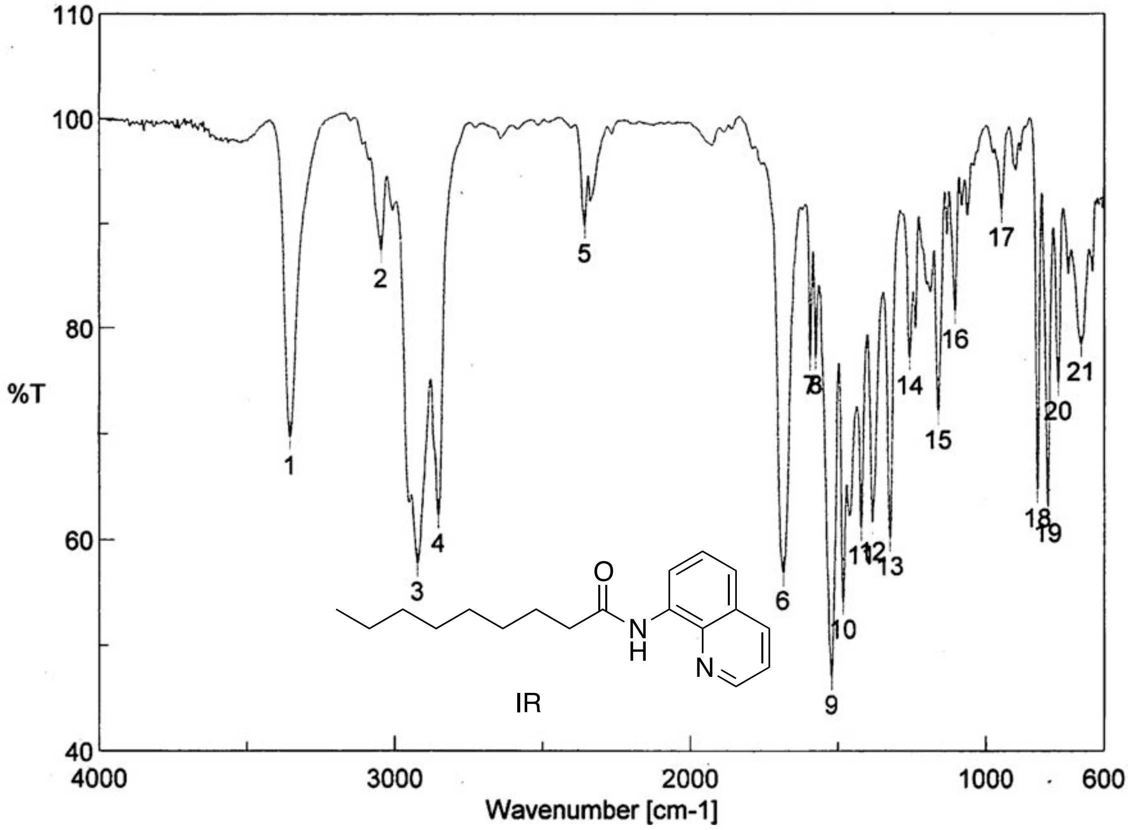

Supplementary Figure S57 | IR spectrum of *N*-(quinolin-8-yl)nonanamide (**3u**)

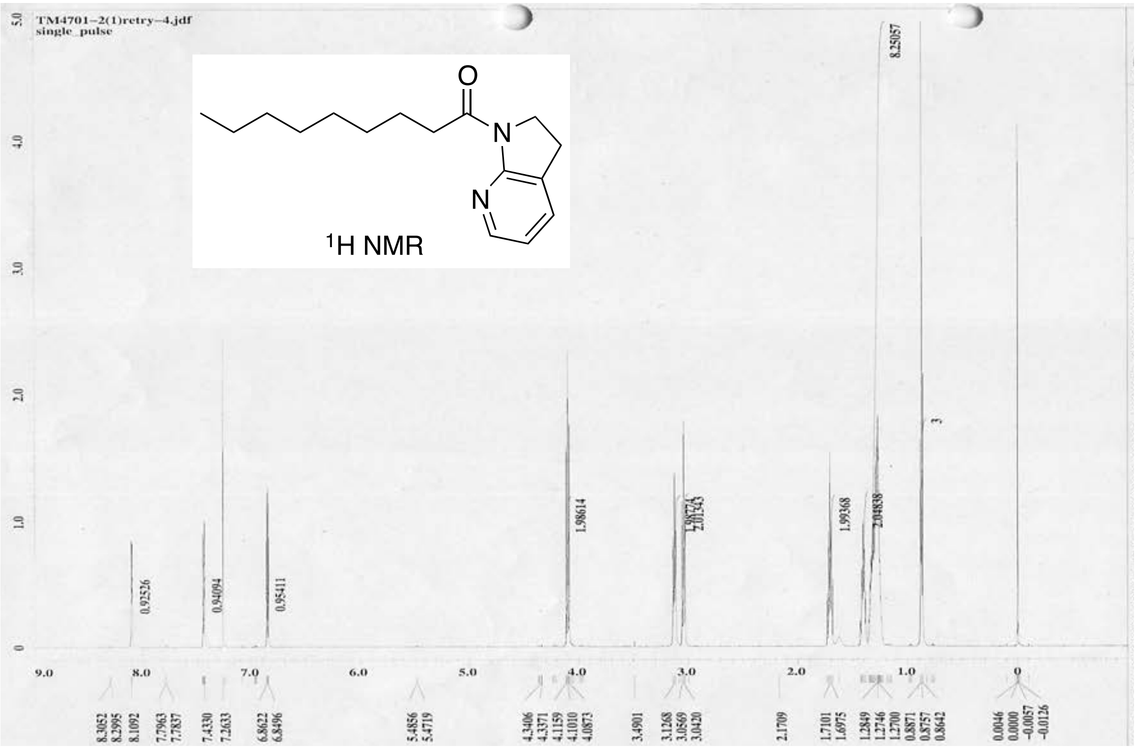

Supplementary Figure S58 | ^{1}H NMR spectrum of 1-(2,3-dihydro-1*H*-pyrrolo[2,3-*b*]pyridin-1-yl)nonan-1-one (**3v**)

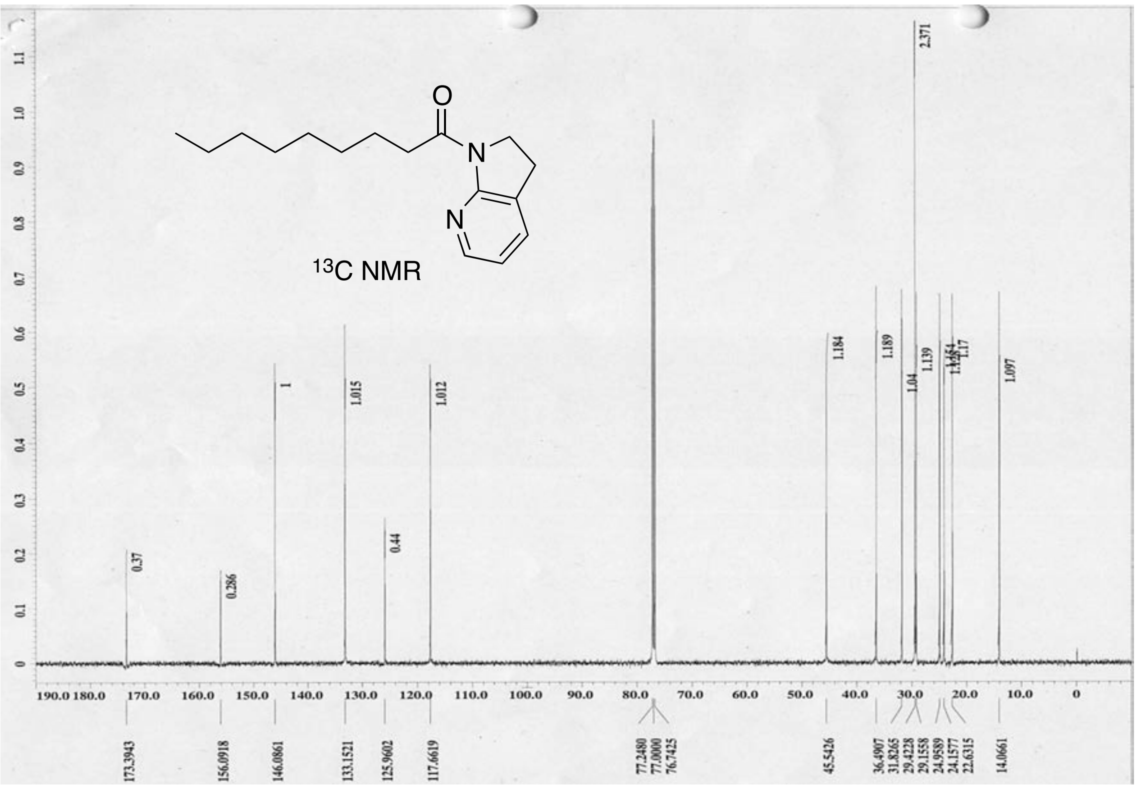

Supplementary Figure S59 | ^{13}C NMR spectrum of 1-(2,3-dihydro-1*H*-pyrrolo[2,3-*b*]pyridin-1-yl)nonan-1-one (**3v**)

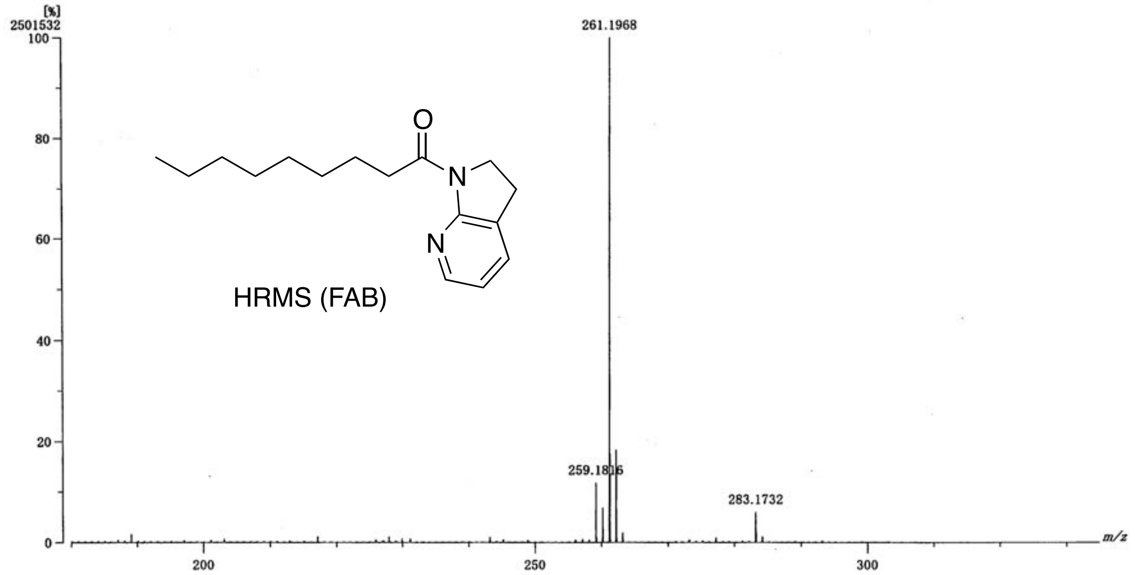

Supplementary Figure S60 | HRMS (FAB) of 1-(2,3-dihydro-1*H*-pyrrolo[2,3-*b*]pyridin-1-yl)nonan-1-one (**3v**)

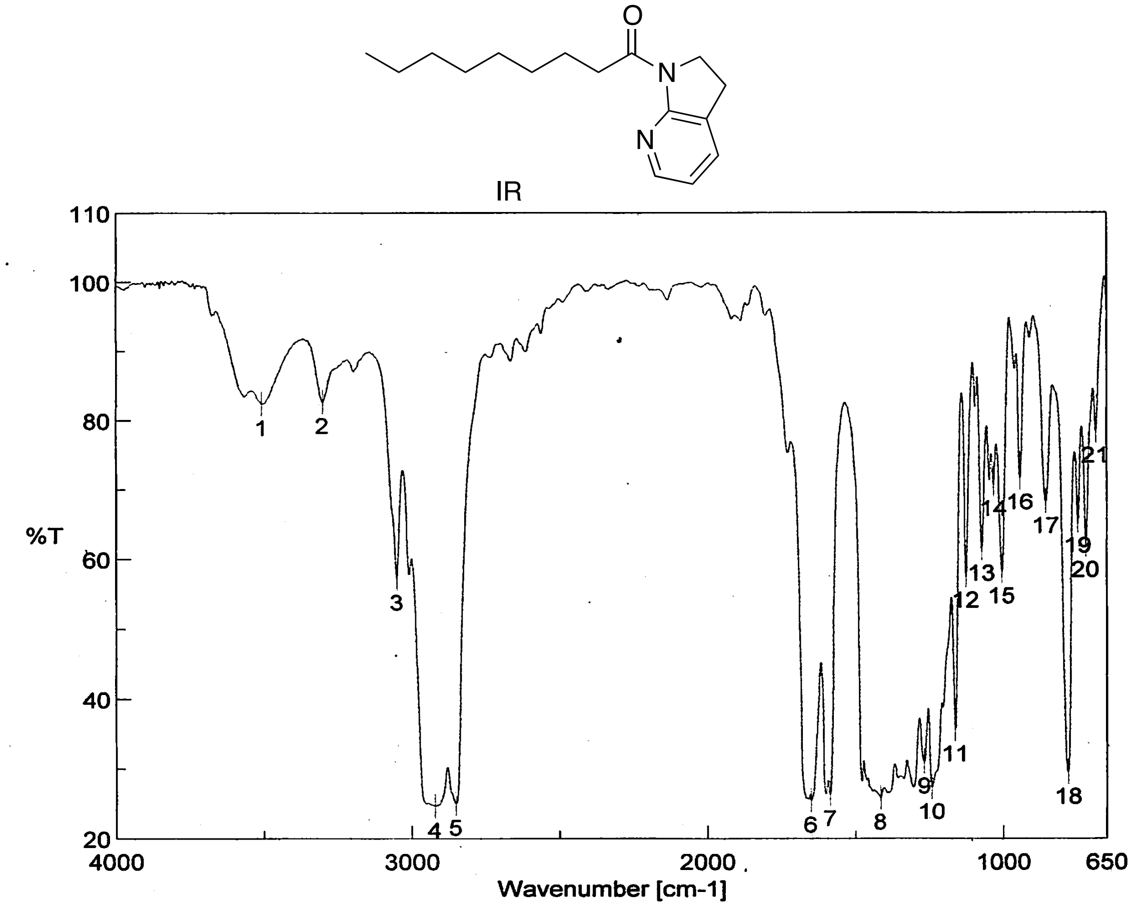

Supplementary Figure S61 | IR spectrum of 1-(2,3-dihydro-1*H*-pyrrolo[2,3-*b*]pyridin-1-yl)nonan-1-one (**3v**)

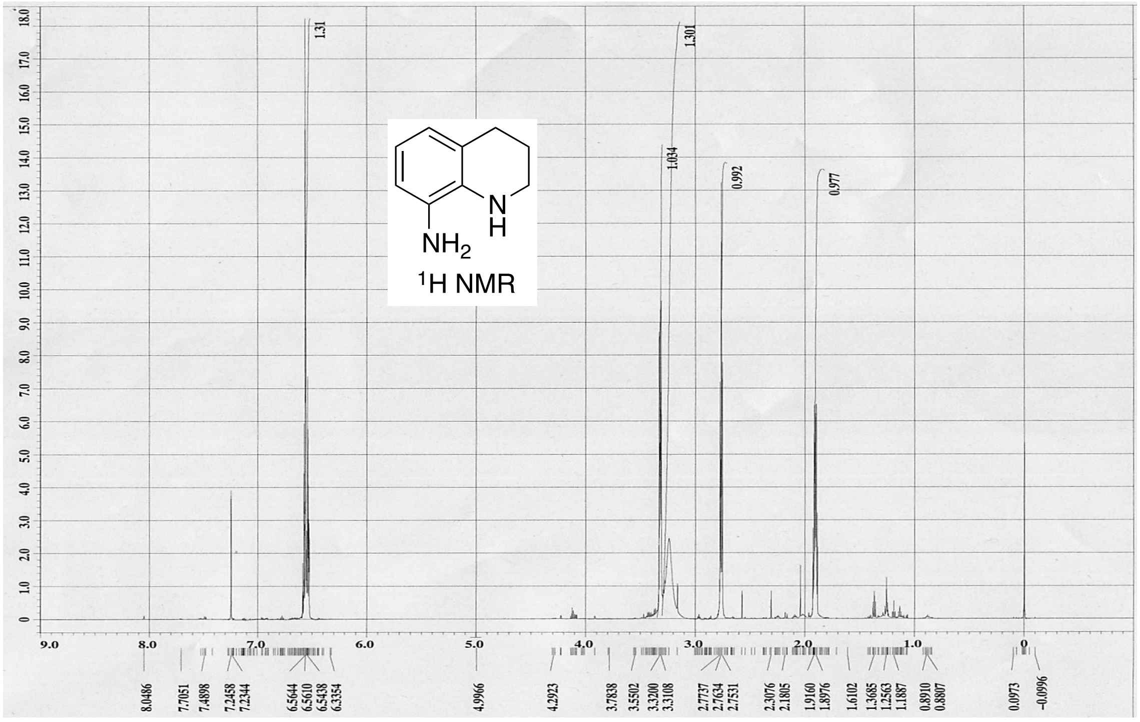

Supplementary Figure S62 | ^{1}H NMR spectrum of 1,2,3,4-tetrahydroquinolin-8-amine (**5u**)

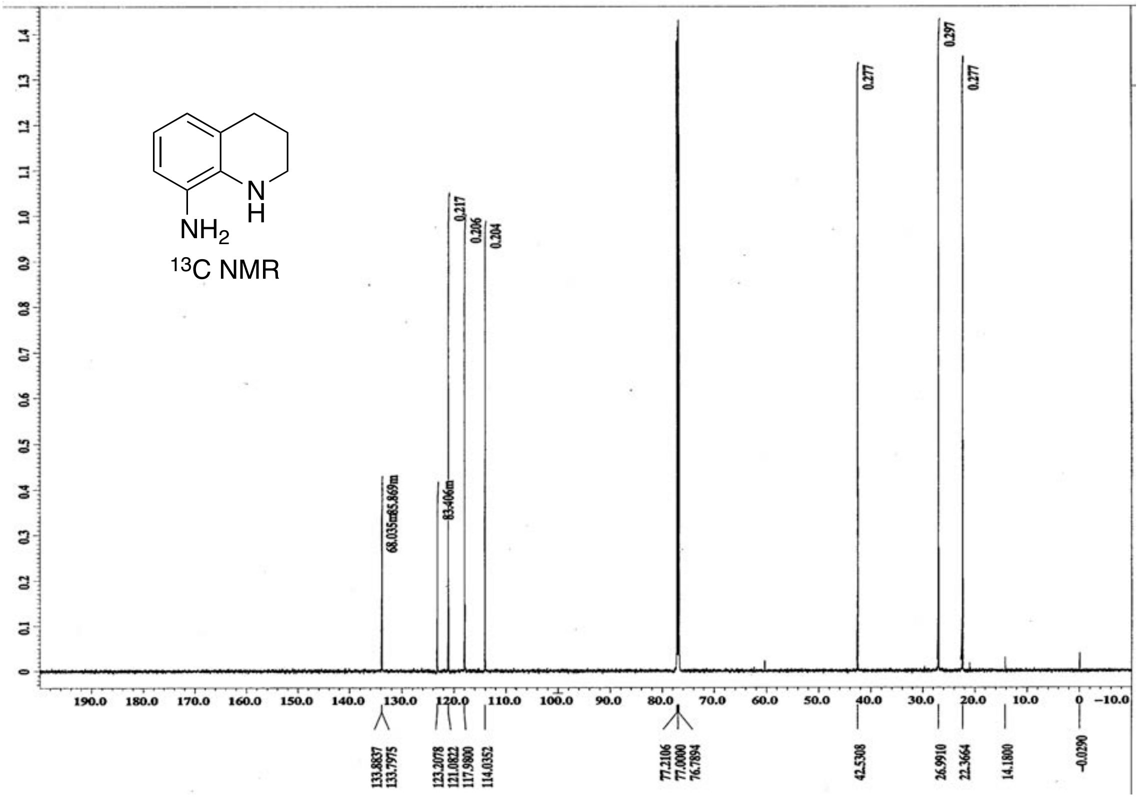

Supplementary Figure S63 | ^{13}C NMR spectrum of 1,2,3,4-tetrahydroquinolin-8-amine (**5u**)

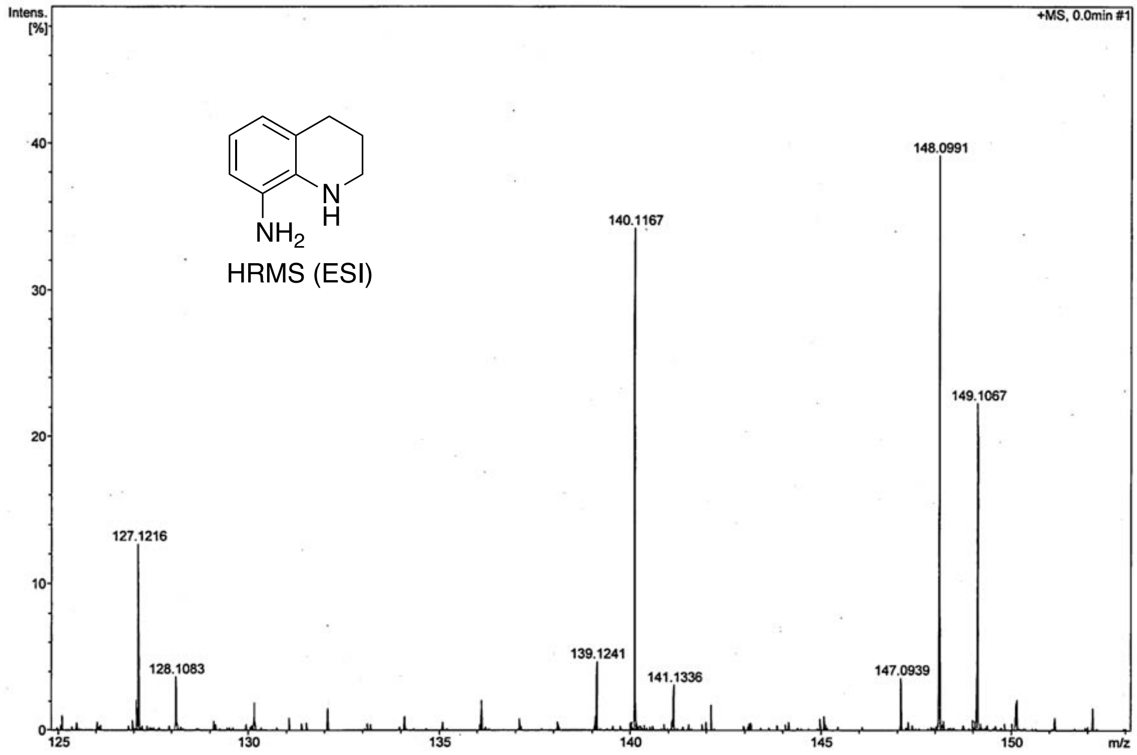

Supplementary Figure S64 | HRMS (ESI) of 1,2,3,4-tetrahydroquinolin-8-amine (**5u**)

Supplementary Table S1 | Catalytic hydrogenation of amides 3 with low concentration of precatalyst.

Our results

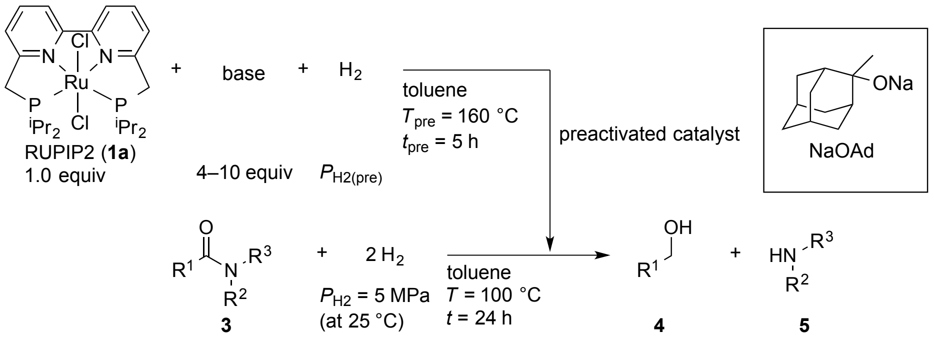

Bergens's results

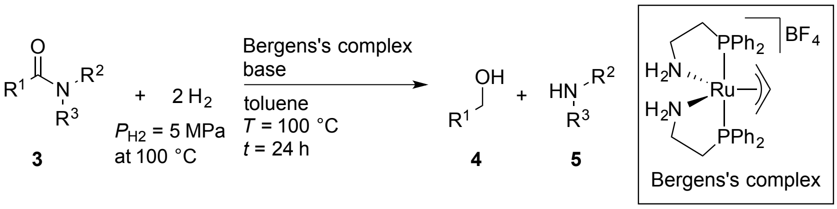

entry	3	precursor	base	conditions	result[a] (%) conv.	yield	TON
1		**1a** (0.1 mol %)	NaOAd (1 mol %)	$P_{H2(pre)}$ = 1 MPa<br>$[Ru]_0$ = 1.1 mM<br>$[\mathbf{3}]_0$ = 1.1 M	52	EtOH 53	530
2	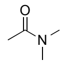	**1a** (0.1 mol %)	NaOAd (4 mol %)	$P_{H2(pre)}$ = 1 MPa<br>$[Ru]_0$ = 1.1 mM<br>$[\mathbf{3}]_0$ = 1.1 M	75	EtOH 74	740
3		Bergens' complex (0.1 mol %)	$[(CH_3)_3Si]_2NK$ (4 mol %)	—	—	50	500
4		**1a** (0.1 mol %)	NaOAd (1 mol %)	$P_{H2(pre)}$ = 8 MPa<br>$[Ru]_0$ = 1.1 mM<br>$[\mathbf{3}]_0$ = 1.1 M	74	EtOH 72<br>aniline 76	720
5	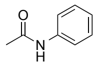	**1a** (0.1 mol %)	NaOAd (4 mol %)	$P_{H2(pre)}$ = 8 MPa<br>$[Ru]_0$ = 1.1 mM<br>$[\mathbf{3}]_0$ = 1.1 M	>99	EtOH 92<br>aniline >99	920
6		**1a** (0.1 mol %)	NaH (1 mol %)	$P_{H2(pre)}$ = 1 MPa<br>$[Ru]_0$ = 1.1 mM<br>$[\mathbf{3}]_0$ = 1.1 M	81	EtOH 77<br>aniline 79	770
7		Bergens' complex (0.1 mol %)	$[(CH_3)_3Si]_2NK$ (4 mol %)	—	—	70	700
8	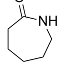	**1a** (0.1 mol %)	NaOAd (1 mol %)	$P_{H2(pre)}$ = 1 MPa<br>$[Ru]_0$ = 1.1 mM<br>$[\mathbf{3}]_0$ = 1.1 M	97	$HO(CH_2)_6NH_2$ 97	970
9		Bergens' complex (0.1 mol %)	$[(CH_3)_3Si]_2NK$ (5 mol %)	—	—	23	230
10	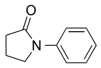	**1a** (0.01 mol %,)	NaOAd (0.1 mol %)	$P_{H2(pre)}$ = 8 MPa<br>$[Ru]_0$ = 0.30 mM<br>$[\mathbf{3}]_0$ = 3.0 M	77	$HO(CH_2)_4NHPh$ 77	7700
11		Bergens' complex (0.01 mol %)	NaOMe (5 mol %)	—	—	71.2	7120

[a] NMR analysis (mesitylene was used as internal standard)

Supplementary Table S2 | Catalytic hydrogenation of various amides **3** (**Fig. 3 and Figs. 4a–c**).

Conditions A

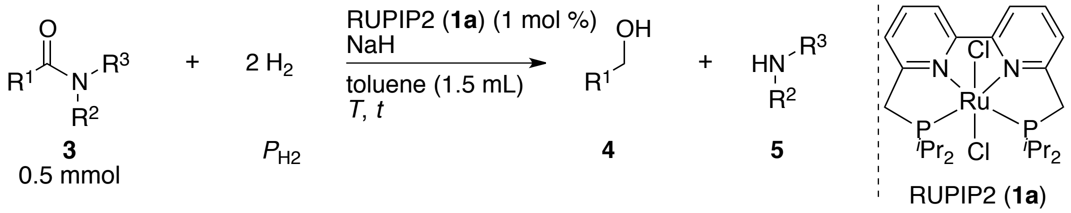

Conditions B

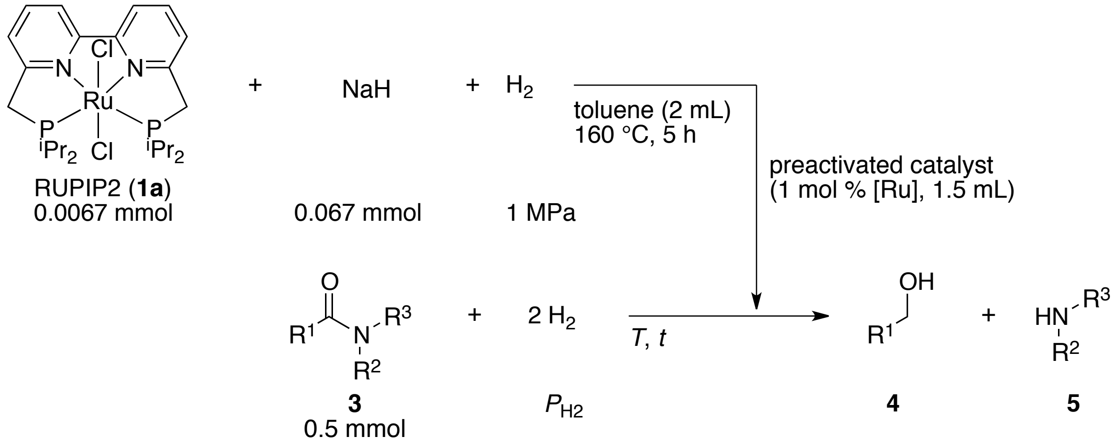

entry	3	conditions	NaH /mol %	P /MPa	T /°C	t /h	result[a] (%)		
							conv.	**4**	**5**
1	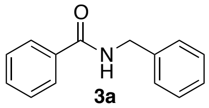 **3a**	A	10	1	110	24	87	86	86
2		A[b]	5	1	110	24	72	73	74
3		B[c]	10	1	110	15	18	18	14
4	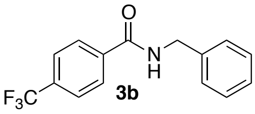 **3b**	A	10	0.5	80	24	95	94	92
5	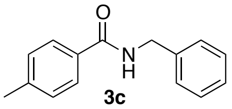 **3c**	A	10	2	110	39	90	93	89
6	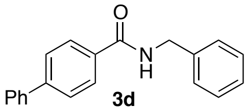 **3d**	A	6	2	120	24	94	93	82
7	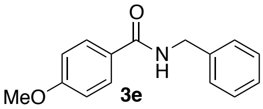 **3e**	A	10	2	120	24	84	84	82
8	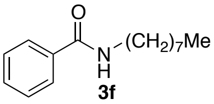 **3f**	A	6	2	120	24	84	84	86
9	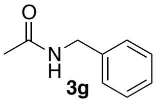 **3g**	B	10	2	120	24	85	—	84
10	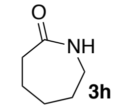 **3h**	A	6	3	130	39	88	86[d]	1[e]
11	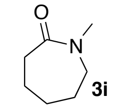 **3i**	A	6	3	130	48	65	66[d]	—
12	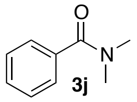 **3j**	B	10	1	110	24	98	95	—
13	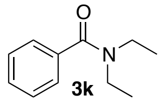 **3k**	B	10	3	130	24	99	94	—
14	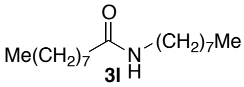 **3l**	A	6	3	130	39	92	89	86
15	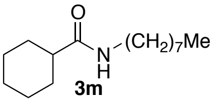 **3m**	A	10	4	140	96	96	96	94

[a] NMR analysis (mesitylene was used as internal standard)

[b] RUPIP2 (0.25 mol %) was used

[c] RUPCY3 (**1e**) was used.
Catalyst was preactivated: $P_{H2(pre)}$ = 8 MPa, T_{pre} = 160 °C, t_{pre} = 5 h.

[d] Yield of aminoalcohol

[e] Yield of azepane ($C_6H_{13}N$)

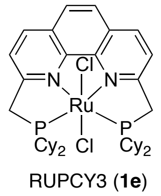

RUPCY3 (**1e**)

entry	3	conditions	NaH /mol %	*P* /MPa	*T* /°C	*t* /h	result[a] (%) conv.	4	5
16	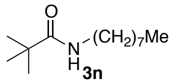 **3n**	A	10	8	160	96	97	70	87
17		A[f]	10	8	160	96	93	71	92
18	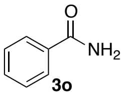 **3o**	A	6	8	160	39	99	92	—
19	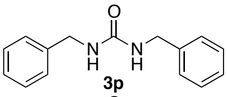 **3p**	B[g,h]	6	6	160	24	99	57	91
20	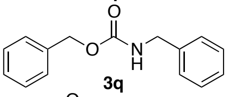 **3q**	B[g,i]	6	6	160	24	99	**4a** 99<br>MeOH 64	99
21	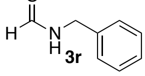 **3r**	A	6	8	60	24	99	71	95
22	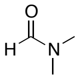	B	10	8	60	15	99	52	—
23		B	10	2	120	27	99	56	—
24	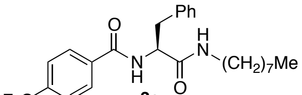 **3s**	A[j]	12	8	160	24	99	**4b** 93<br>**6s** 96	93
25	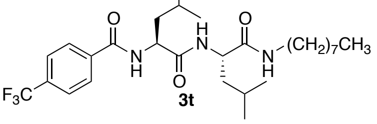 **3t**	A[j]	20	8	160	72	99	**4b** 99<br>**6t** 83	89
26	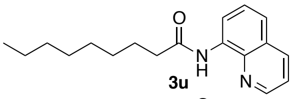 **3u**	B	10	3	130	24	99	93	**5u** 99
27	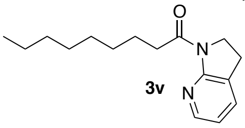 **3v**	B	10	3	130	24	99	99	99
28	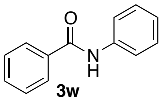 **3w**	A[b]	6	0.5	80	48	99	93	88

[a] NMR analysis (mesitylene or DMF was used as internal standard)

[b] RUPIP2 (0.25 mol %) was used

[f] Hg (150 mol %) was used

[g] RUPCY2 (**1b**) was used.
Catalyst was preactivated: $P_{H2(pre)}$ = 8 MPa, T_{pre} = 160 °C, t_{pre} = 2 h.

[h] $[Ru]_0$ =10 mM [i] $[Ru]_0$ =5 mM

[j] RUPIP2 (2 mol %) was used

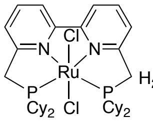

RUPCY2 (**1b**)

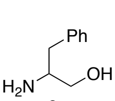

6s

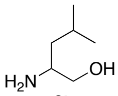

6t

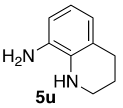

5u

Supplementary Methods

General

All experiments were performed under an Ar atmosphere unless otherwise noted. ^{1}H NMR spectra were measured on JEOL ECA–600 (600 MHz), JEOL ECA–500 (500 MHz) at ambient temperature. Data were recorded as follows: chemical shift in ppm from internal tetramethylsilane on the δ scale, multiplicity (br = broad, s = singlet, d = doublet, t = triplet, q = quartet, quin = quintet m = multiplet), coupling constant (Hz), integration, and assignment. ^{13}C NMR spectra were measured on JEOL ECA–600 (150 MHz), JEOL ECA–500 (126 MHz) at ambient temperature. Chemical shifts were recorded in ppm from the solvent resonance employed as the internal standard (chloroform-*d* at 77.00 ppm or tetramethylsilane at 0 ppm). ^{31}P NMR spectra were measured on JEOL ECA–600 (243 MHz), JEOL ECA–500 (202 MHz) at ambient temperature. Chemical shifts were recorded in ppm from the solvent resonance employed as the external standard (phosphoric acid (85 wt% in H_2O) at 0.0 ppm). High–resolution mass spectra (HRMS) were obtained from JEOL JMS700 (FAB), PE Biosystems QSTAR (ESI). IR spectra were obtained from JASCO FT/IR6100. For thin–layer chromatography (TLC) analysis through this work, Merck precoated TLC plates (silica gel 60 GF254 0.25 mm) were used. The products were purified by preparative column chlomatography on silica gel 60 N (spherical, neutral) (40–100 μm; Kanto).

Materials.

Benzylamine, 4-methoxybenzoyl chloride, benzoyl chloride, 2,3-dihydro-7-azaindole, urea, β-citronellol (**4x**), *trans*-5-decen-1-ol (**4y**), diisopropylamine, (*L*)-phenylalanine, octylamine, *N,N*-dimethylbenzamide (**3j**), HCl (2.0 M Et_2O solution) and ε-caprolactam (**3h**) were purchased from Aldrich. Biphenyl-4-carboxylic acid, $NaHCO_3$, $RuCl_2(PPh_3)_3$, chlorodicyclohexylphosphine, chlorodiisopropylphosphine, chlorodi-*tert*-butylphosphine, BH_3–THF complex (1.0 M in THF), (*L*)-leucine, *N*-benzylacetamide (**3g**), 1-(3-Dimethylaminopropyl)-3-ethylcarbodiimide hydrochloride (EDCI), Hg(0), EtOAc and hexane were purchased from Wako Pure Chemical industries, Ltd. *n*-Nonanoyl chloride, I_2, $Na_2S_2O_3$•$5H_2O$, pyridine, *N,N*-dimethyl formamide, benzanilide (**3w**), Et_2O, trifluoroacetic acid, NaH (55% oil dispersion), mesitylene, MeCN (anhydrous), THF (anhydrous), dichloromethane

(anhydrous), hexane (anhydrous), toluene (anhydrous), dichloromethane, Na_2SO_4, Et_3N, Et_2NH, *N*-methylmorpholine, Bu^nLi (1.5 M in hexane), morpholine, pivaloyl chloride, NaOH, K_2CO_3 and aq. HCl were purchased from Kanto Chemicals, Ltd. 4-(Trifluoromethyl)benzoyl chloride, 4-methylbenzoyl chloride, thionyl chloride, neocuproine hemihydrate, *N*-methylcaprolactam (**3i**), benzyl chloroformate, *N*-benzylformamide (**3r**), 6,6'-bi-2-picoline, 1,2,3-benzotriazol-1-ol monohydrate (HOBt・H_2O), 1,1,2,2-tetrachloroethane, 2-methyl-2-adamantanol, 8-aminoquinoine, benzamide (**3o**) and cyclohexanecarbonyl chloride were purchased from TCI, Ltd. Boc–(*L*)-leucine–OH ・ H_2O was purchased from Watanabe Chemical Industries, Ltd. *N*-benzylbenzamide (**3a**) was purchased from Across Organics, Ltd. $CDCl_3$ was purchased from Cambridge Isotope Laboratories, Inc. Hydrogen gas was purchased from Alpha System. These chemicals were used without further purification.

N-benzyl-4-(trifluoromethyl)benzamide (**3b**),[1] *N*-benzyl-4-methylbenzamide (**3c**),[2,3] *N*-benzylbiphenyl-4-carboxamide (**3d**),[4] *N*-benzyl-4-methoxybenzamide (**3e**),[5] *N*-octylbenzamide (**3f**),[6] *N,N*-diethylbenzamide (**3k**),[7] *N*-octylnonanamide (**3l**),[4,8] *N*-octylcyclohexanecarboxamide (**3m**),[4] *N*-octylpivalamide (**3n**),[4] *N,N'*-dibenzylurea (**3p**),[9] *N,O*-dibenzyl carbamate (**3q**),[10] dichloro(6,6'-bis((di-*tert*-butylphosphino)methyl)-2,2'-bipyridine)–ruthenium (II) (**1c**),[11] dichloro(2,9-bis((di-*tert*-butylphosphino)methyl)-1,10-phenanthroline)–ruthenium (II) (**1f**)[12] and 8-amino-1,2,3,4-tetra-hydroquinoline (**5u**)[13] are all known compounds.

Experimental procedures

Preparation of ligands and base

6,6'-bis((diisopropylphosphino)methyl)-2,2'-bipyridine –diborane complex

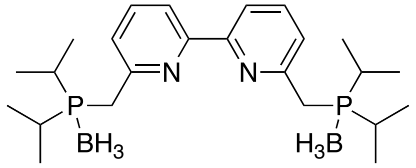

To an anhydrous THF (10 mL) solution of 6,6'-bi-2-picoline (184.2 mg, 1.0 mmol) was added a THF solution of lithium diisopropylamide {prepared by mixing

diisopropylamine (0.85 mL, 6.0 mmol) and 1.5 M hexane solution of Bu^nLi (4.0 mL, 6.0 mmol) in THF (10 mL), followed by being stirred for 10 min at 0 °C under Ar} by cannula at 0 °C (H_2O-ice) under Ar, and the mixture was stirred at room temperature for 1 h. To the bluish-purple suspension was added chlorodiisopropylphosphine (314.6 μL, 2.0 mmol) dropwise at the same temperature, and it was stirred for 4 h. To the reaction mixture was added BH_3–THF complex (1 M in THF, 10 mL, 10 mmol) at room temperature, and the mixture was stirred for 12 h. The mixture was quenched by adding a small portion of water (*ca.* <5 mL) at 0 °C, and the organic phase was removed *in vacuo* (*ca.* 50 mmHg, 40 °C). The residue was dissolved into CH_2Cl_2 (20 mL), washed with H_2O (40 mL), and extracted with CH_2Cl_2 (20 mL×5) and then washed with brine. The organic layer was dried over Na_2SO_4 and filtrated. The evaporation of the filtrate gave a yellow crude oil, which was purified by column chromatography on silica gel (CH_2Cl_2/hexane = 8/1) to afford the target compound (244.3 mg, 55%) as white solid. IR (KBr): 3423, 3068, 2969, 2934, 2876, 2366, 2253, 1572, 1436 cm^{-1}. ^1H NMR (600 MHz, $CDCl_3$): δ 8.24 (d, 2H, J = 7.6 Hz, $C_{10}H_6N_2$), 7.76 (t, 2H, J = 7.6 Hz, $C_{10}H_6N_2$), 7.34 (d, 2H, J = 7.6 Hz, $C_{10}H_6N_2$), 3.33 (d, 4H, J = 11.0 Hz, PCH_2), 2.12–2.23 (m, 4H, $CH(CH_3)_2$), 1.17–1.27 (m, 24H, $CH(CH_3)_2$). 0.10–0.70 (br, 6H, BH_3). ^{13}C NMR (151 MHz, $CDCl_3$): δ 155.3, 154.2 (d, $^2J_{PC}$ = 7.2 Hz), 137.3, 124.9, 118.8, 30.6 (d, $^1J_{PC}$ = 26.0 Hz), 21.8 (d, $^1J_{PC}$ = 31.8 Hz), 17.0 (d, $^2J_{PC}$ = 5.8 Hz). $^{31}P\{^1H\}$ NMR (243 MHz, $CDCl_3$): δ 36.1 (d, $^1J_{PB}$ = 72.4 Hz), HRMS (ESI, $(M+H)^+$) Calcd for $C_{24}H_{44}B_2N_2P_2^+$: 445.3247; Found: m/z = 445.3247. (Supplementary Figs. S12–S16).

6,6'-bis((dicyclohexylphosphino)methyl)-2,2'-bipyridine–diborane complex

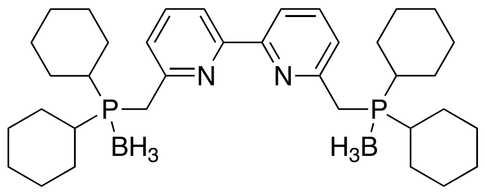

To an anhydrous THF (60 mL) solution of 6,6'-bi-2-picoline (1850.0 mg, 10 mmol) was added a THF solution of lithium diisopropylamide {prepared by mixing diisopropylamine (8.41 mL, 60 mmol) and 1.5 M hexane solution of Bu^nLi (40 mL, 60 mmol) in THF (30 mL), followed by being stirred for 10 min at 0 °C under Ar} by

cannula at 0 °C (H_2O-ice) under Ar, and the mixture was stirred at room temperature for 1 h. To the bluish-purple suspension was added chlorodicyclohexylphosphine (4.4 mL, 20 mmol) dropwise at the same temperature, and it was stirred for 2 h. To the reaction mixture was added BH_3–THF complex (1 M in THF, 100 mL, 100 mmol) at room temperature, and the mixture was stirred for 12 h. The mixture was quenched by adding a small portion of water (*ca.* <5 mL) at 0 °C, and the organic phase was removed *in vacuo* (*ca.* 50 mmHg, 40 °C). The residue was dissolved into CH_2Cl_2 (50 mL), washed with H_2O (100 mL), and extracted with CH_2Cl_2 (50 mL×4) and then washed with brine. The organic layer was dried over Na_2SO_4 and filtrated. The evaporation of the filtrate gave a yellow residue, which was suspended by a small portion of THF (*ca.* <10 mL), and slurry was filtrated to afford the target compound (2417.6 mg, 40%) as white solid. IR (KBr): 3435, 2931, 2849, 2378, 2332, 1572, 1437 cm^{-1}. ^1H NMR (600 MHz, $CDCl_3$): δ 8.29 (d, 2H, J = 8.3 Hz, $C_{10}H_6N_2$), 7.75 (t, 2H, J = 8.3 Hz, $C_{10}H_6N_2$), 7.31 (d, 2H, J = 7.6 Hz, $C_{10}H_6N_2$), 3.31 (d, 4H, J = 11.0 Hz, PCH_2), 1.60–2.00 (m, 24H, C_6H_{11}), 1.11–1.50 (m, 20H, C_6H_{11}). ^{13}C NMR (151 MHz, $CDCl_3$): δ 155.2, 154.5 (d, $^2J_{PC}$ = 7.2 Hz), 137.2, 125.0, 118.7, 31.5 (d, $^1J_{PC}$ = 30.3 Hz), 30.5 (d, $^1J_{PC}$ = 27.5 Hz), 27.0 (d, $^2J_{PC}$ = 11.6 Hz), 26.9 (d, $^2J_{PC}$ = 11.6 Hz), 26.7, 26.6, 26.0. $^{31}P\{^1H\}$ NMR (243 MHz, $CDCl_3$): δ 28.8 (d, $^1J_{PB}$ = 39.2 Hz), HRMS (ESI, $(M+H)^+$) Calcd for $C_{36}H_{60}B_2N_2P_2^+$: 605.4502; Found: *m/z* = 605.4502. (Supplementary Figs. S17–S21)

2,9-bis((diisopropylphosphino)methyl)-1,10-phenanthroline−diborane complex

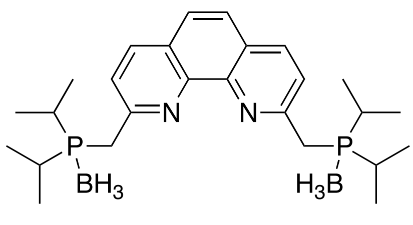

To an anhydrous THF (100 mL) solution of neocuproine hemihydrate (1041.5 mg, 5.0 mmol) was added a THF solution of lithium diisopropylamide {prepared by mixing diisopropylamine (4.2 mL, 30 mmol) and 1.5 M hexane solution of Bu^nLi (20.0 mL, 30.0 mmol) in THF (50 mL), followed by being stirred for 10 min at 0 °C under Ar} by cannula at 0 °C (H_2O-ice) under Ar, and the mixture was stirred at room temperature for 1 h. To the bluish-purple suspension was added chlorodiisopropylphosphine (1.59 mL,

10 mmol) dropwise at the same temperature, and it was stirred for 12 h. To the reaction mixture was added BH_3–THF complex (1 M in THF, 50 mL, 50 mmol) at room temperature, and the mixture was stirred for 12 h. The mixture was quenched by adding a small portion of water (*ca.* <5 mL) at 0 °C, and the organic phase was removed *in vacuo* (*ca.* 50 mmHg, 40 °C). The residue was dissolved into CH_2Cl_2 (50 mL), washed with H_2O (100 mL), and extracted with CH_2Cl_2 (50 mL×5) and then washed with brine. The organic layer was dried over Na_2SO_4 and filtrated. The evaporation of the filtrate gave a yellow crude oil, which was purified by column chromatography on silica gel ($CHCl_3$/EtOAc/hexane = 1/1/3) to afford the target compound (1248 mg, 53%) as pale yellow solid. ^{1}H NMR (600 MHz, $CDCl_3$): δ 8.19 (d, 2H, J = 8.3 Hz, $C_{12}H_6N_2$), 7.76 (d, 2H, J = 7.6 Hz, $C_{12}H_6N_2$), 7.76 (s, 2H, $C_{12}H_6N_2$), 3.63 (d, 4H, J = 11.7 Hz, PCH_2), 2.18-2.26 (m, 4H, $CH(CH_3)_2$), 1.16-1.24 (m, 24H, $CH(CH_3)_2$). ^{13}C NMR (151 MHz, $CDCl_3$): δ 155.3 (d, $^2J_{PC}$ = 4.3 Hz), 145.3, 136.2, 127.5, 126.1, 124.7, 31.9 (d, $^1J_{PC}$ = 24.6Hz), 22.2 (d, $^1J_{PC}$ = 31.8 Hz), 17.1 (d, $^2J_{PC}$ = 4.3 Hz). ^{31}P{^{1}H} NMR (243 MHz, $CDCl_3$): δ 36.2 (d, $^1J_{PB}$ = 65.9 Hz). (ESI, $(M+H)^+$) Calcd for $C_{26}H_{44}B_2N_2P_2^+$: 469.3247; Found: m/z = 469.3235. (Supplementary Figs. S22–S25)

2,9-bis((dicyclohexylphosphino)methyl)-1,10-phenanthroline−diborane complex

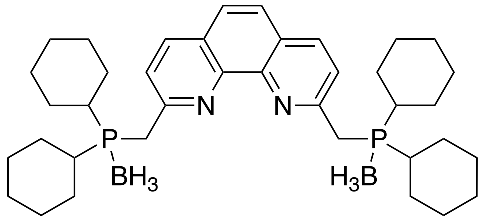

To an anhydrous THF (100 mL) solution of neocuproine hemihydrate (1041.5 mg, 5.0 mmol) was added a THF solution of lithium diisopropylamide {prepared by mixing diisopropylamine (4.2 mL, 30 mmol) and 1.5 M hexane solution of Bu^{n}Li (20.0 mL, 30.0 mmol) in THF (50 mL), followed by being stirred for 10 min at 0 °C under Ar} by cannula at 0 °C (H_2O-ice) under Ar, and the mixture was stirred at room temperature for 1 h. To the bluish-purple suspension was added chlorodicyclohexylphosphine (2.2 mL, 10 mmol) dropwise at the same temperature, and it was stirred for 12 h. To the reaction mixture was added BH_3–THF complex (1 M in THF, 50 mL, 50 mmol) at room

temperature, and the mixture was stirred for 12 h. The mixture was quenched by adding a small portion of water (*ca.* <5 mL) at 0 °C, and the organic phase was removed *in vacuo* (*ca.* 50 mmHg, 40 °C). The residue was dissolved into CH_2Cl_2 (50 mL), washed with H_2O (100 mL), and extracted with CH_2Cl_2 (50 mL×5) and then washed with brine. The organic layer was dried over Na_2SO_4 and filtrated. The evaporation of the filtrate gave a pale red residue, which was suspended by a small portion of EtOAc (*ca.* <10 mL), and slurry was filtrated with acetone to afford the target compound (1580 mg, 50%) as pale red solid. ^{1}H NMR (600 MHz, $CDCl_3$): δ 8.16 (d, 2H, J = 8.3 Hz, $C_{12}H_6N_2$), 7.75 (s, 2H, $C_{12}H_6N_2$), 7.7 (d, 2H, J = 8.3 Hz, $C_{12}H_6N_2$), 3.57 (d, 4H, J = 11.3 Hz, PCH_2), 1.60-2.03 (m, 24H, C_6H_{11}), 1.13-1.57 (m, 20H, C_6H_{11}). ^{13}C NMR (149 MHz, $CDCl_3$): δ 155.3 (d, $^2J_{PC}$ = 5.8 Hz), 145.5, 136.0, 127.4, 126.1, 124.6, 31.9 (d, $^1J_{PC}$ = 31.5 Hz), 31.5 (d, $^1J_{PC}$ = 25.8 Hz), 27.1 (d, $^2J_{PC}$ = 11.6 Hz), 27.0 (d, $^2J_{PC}$ = 11.6 Hz), 26.8, 26.6, 25.9. $^{31}P\{^1H\}$ NMR (243 MHz, $CDCl_3$): δ 28.5. (ESI, $(M+H)^+$) Calcd for $C_{38}H_{60}B_2N_2P_2^+$: 629.4503; Found: m/z = 629.4503. (Supplementary Figs. S26–S29)

Preparation of ruthenium complexes

Dichloro(6,6'-bis((diisopropylphosphino)methyl)-2,2'-bipyridine)–ruthenium (II), RUPIP2 (1a)

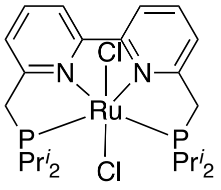

A degassed morpholine (10 mL) solution of 6,6'-bis((diisopropylphosphino)methyl)-2,2'-bipyridine–diborane complex (300.0 mg, 0.68 mmol) was heated at 130 °C for 2 h under Ar. The solution was cooled to room temperature and morpholine was removed *in vacuo* (*ca.* 1 mmHg, room temperature). To the residue was added sequentially dichlorotris(triphenylphosphino)ruthenium (II) (648.6 mg, 0.68 mmol) and an anhydrous toluene (15 mL). The resulting mixture was heated at 110 °C for 2 h under Ar, and was cooled to room temperature. Then to the mixture was added an anhydrous hexane (40 mL) to afford the purple suspension. The mixture of the suspension was stirred at room temperature for 1 h and filtered through a filtration paper. The obtained purple solid was dried *in vacuo* (*ca.* 0.1 mmHg, room

temperature). This solid was purified by column chromatography on silica gel ($CHCl_3$/EtOAc = 5/1) to afford RUPIP2 (**1a**) as purple solid (204.3 mg, 51%). ^{1}H NMR (600 MHz, $CDCl_3$): δ 7.89 (d, 2H, J = 8.3 Hz, $C_{10}H_6N_2$), 7.69 (t, 2H, J = 7.6 Hz, $C_{10}H_6N_2$), 7.59 (d, 2H, J = 8.3 Hz, $C_{10}H_6N_2$), 3.89 (d, 4H, J = 7.6 Hz, PCH_2), 2.67–2.78 (m, 4H, $CH(CH_3)_2$), 1.35–1.43 (m, 24H, $CH(CH_3)_2$). ^{13}C NMR (151 MHz, $CDCl_3$): δ 163.3, 158.3, 134.4, 121.9, 120.0, 42.1 (d, $^1J_{PC}$ = 20.2 Hz), 25.4, 20.5, 19.4. ^{31}P{^{1}H} NMR (243MHz, $CDCl_3$): δ 60.4. HRMS (ESI, $(M–Cl)^+$) Calcd for $C_{24}H_{38}ClN_2P_2Ru^+$: 553.1242; Found: m/z = 553.1240. (Supplementary Figs. S30–S33)

Dichloro(6,6'-bis((dicyclohexylphosphino)methyl)-2,2'-bipyridine)–ruthenium (II), RUPCY2 (1b)

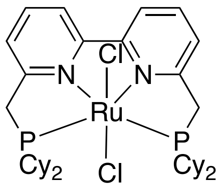

A degassed morpholine (20 mL) solution of 6,6'-bis((dicyclohexylphosphino)methyl)-2,2'-bipyridine−diborane complex (604.4 mg, 1.0 mmol) was heated at 130 °C for 2 h under Ar. The solution was cooled to room temperature and morpholine was removed *in vacuo* (*ca.* 1 mmHg, room temperature). To the residue was added sequentially dichlorotris(triphenylphosphino)ruthenium (II) (958.8 mg, 1.0 mmol) and an anhydrous toluene (20 mL). The resulting mixture was heated at 110 °C for 3 h under Ar, and was cooled to room temperature. Then to the mixture was added an anhydrous hexane (40 mL) to afford the purple suspension. The mixture of the suspension was stirred at room temperature for 1 h and filtered through a filtration paper. The obtained purple solid was dried *in vacuo* (*ca.* 0.1 mmHg, room temperature). This solid was purified by column chromatography on silica gel ($CHCl_3$/ EtOAc = 5/1) to afford RUPCY2 (**1b**) as purple solid (435.1 mg, 58%). ^{1}H NMR (500 MHz, $CDCl_3$): δ 7.86 (d, 2H, J = 7.4 Hz, $C_{10}H_6N_2$), 7.66 (t, 2H, J = 7.5 Hz, $C_{10}H_6N_2$), 7.56 (d, 2H, J = 7.5 Hz, $C_{10}H_6N_2$), 3.87 (d, 4H, J = 8.1 Hz, PCH_2), 2.41 (br, 4H, C_6H_{11}), 2.18 (d, 4H, J = 12.1 Hz Hz, C_6H_{11}), 2.05 (d, 4H, J = 10.9 Hz, C_6H_{11}), 1.54–1.81 (m, 20H, C_6H_{11}), 1.20–1.34 (m, 20H, C_6H_{11}). ^{13}C NMR (126 MHz, $CDCl_3$): δ 163.3, 158.3,

134.1, 122.0, 119.9, 40.5 (d, $^1J_{PC}$ = 12.8 Hz), 36.3, 30.3, 29.4, 27.7, 27.5, 26.4. $^{31}P\{^1H\}$ NMR (159 MHz, $CDCl_3$): δ 54.2. HRMS (ESI, $(M–Cl)^+$) Calcd for $C_{36}H_{54}ClN_2P_2Ru^+$: 713.2494; Found: m/z = 713.2476. (Supplementary Figs. S34–S37)

Dichloro(2,9-bis((diisopropylphosphino)methyl)-1,10-phenanthroline)–ruthenium (II), RUPIP3 (1d)

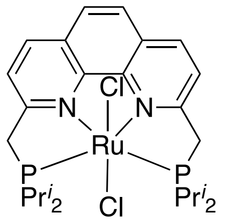

A degassed morpholine (15 mL) solution of 2,9-bis((diisopropylphosphino)methyl)-1,10-phenanthroline−diborane complex (362.2 mg, 0.77 mmol) was heated at 120 °C for 2 h under Ar. The solution was cooled to room temperature and morpholine was removed *in vacuo* (*ca.* 1 mmHg, room temperature). To the residue was added sequentially dichlorotris(triphenylphosphino)ruthenium (II) (738.2 mg, 0.77 mmol) and an anhydrous toluene (15 mL). The resulting mixture was heated at 110 °C for 12 h under Ar, and was cooled to room temperature. Then to the mixture was added an anhydrous hexane (40 mL) to afford the purple suspension. The mixture of the suspension was stirred at room temperature for 1 h and filtered with Et_2O through a filtration paper. The obtained purple solid was dried *in vacuo* (*ca.* 0.1 mmHg, room temperature). This solid was purified by column chromatography on silica gel ($CHCl_3$/acetone = 3/1) to afford RUPIP3 (**1d**) as purple solid (180.4 mg, 38%). ^1H NMR (600 MHz, $CDCl_3$): δ 8.13 (d, 2H, J = 8.3 Hz, $C_{12}H_6N_2$), 7.88 (d, 2H, J = 8.3 Hz, $C_{12}H_6N_2$), 7.81 (s, 2H, $C_{12}H_6N_2$), 4.07 (d, 4H, J = 8.2 Hz, PCH_2), 2.75-2.85 (m, 4H, $CH(CH_3)_2$), 1.38-1.52 (m, 24H, $CH(CH_3)_2$). ^{13}C NMR (151 MHz, $CDCl_3$): δ 163.6, 149.3, 132.9, 128.7, 125.4, 121.7 (d, $^2J_{PC}$ = 5.8 Hz), 43.0 (dd, $^1J_{PC}$ = 17.3 Hz, $^2J_{CC}$ = 7.2 Hz), 25.6 (dd, $^1J_{PC}$ = 8.6 Hz, $^2J_{CC}$ = 7.2 Hz), 20.9, 19.4. $^{31}P\{^1H\}$ NMR (243 MHz, $CDCl_3$): δ 62.9. HRMS (ESI, $(M–Cl)^+$) Calcd for $C_{26}H_{38}ClN_2P_2Ru^+$: 577.1242; Found: m/z = 577.1210. (Supplementary Figs. S38–S41)

Dichloro(2,9-bis((dicyclohexylphosphino)methyl)-1,10-phenanthroline)–ruthenium (II), RUPCY3 (1e)

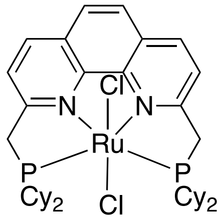

A degassed morpholine (15 mL) solution of 2,9-bis((dicyclohexylphosphino)methyl)-1,10-phenanthroline−diborane complex (628.5 mg, 1.0 mmol) was heated at 120 °C for 2 h under Ar. The solution was cooled to room temperature and morpholine was removed *in vacuo* (*ca.* 1 mmHg, room temperature). To the residue was added sequentially dichlorotris(triphenylphosphino)ruthenium (II) (958.8 mg, 1.0 mmol) and an anhydrous toluene (20 mL). The resulting mixture was heated at 110 °C for 12 h under Ar, and was cooled to room temperature. Then to the mixture was added an anhydrous hexane (40 mL) to afford the purple suspension. The mixture of the suspension was stirred at room temperature for 1 h and filtered with Et_2O through a filtration paper. The obtained purple solid was dried *in vacuo* (*ca.* 0.1 mmHg, room temperature). This solid was purified by column chromatography on silica gel ($CHCl_3$/THF = 10/1) to afford RUPCY3 (**1e**) as purple solid (563.7 mg, 73%). ^{1}H NMR (600 MHz, $CDCl_3$): δ 8.12 (d, 2H, J = 8.3 Hz, $C_{12}H_6N_2$), 7.86 (d, 2H, J = 8.2 Hz, $C_{12}H_6N_2$), 7.80 (s, 2H, $C_{12}H_6N_2$), 4.05 (d, 4H, J = 6.8 Hz, PCH_2), 2.42-2.51 (br, 4H, C_6H_{11}), 2.31 (d, 4H, J = 11.0 Hz C_6H_{11}), 2.12 (d, 4H, J = 12.4 Hz, C_6H_{11}), 1.61-1.94 (m, 20H, C_6H_{11}), 1.18-1.37 (m, 12H, C_6H_{11}). ^{13}C NMR (151 MHz, $CDCl_3$): δ 163.6, 149.3, 132.6, 128.6, 125.3, 121.8, 41.4 (dd, $^1J_{PC}$ = 15.8 Hz, $^2J_{CC}$ = 7.2 Hz), 36.6 (dd, $^1J_{PC}$ = 7.2 Hz, $^2J_{CC}$ = 7.2 Hz), 30.7, 29.4, 27.8, 27.6, 26.4. ^{31}P{^{1}H} NMR (243 MHz, $CDCl_3$): δ 56.8. HRMS (ESI, $(M–Cl)^+$) Calcd for $C_{38}H_{54}ClN_2P_2Ru^+$: 737.2494; Found: m/z = 737.2483. (Supplementary Figs. S42–S45)

Substrate preparation for hydrogenation

(*S*)-3-phenyl-2-(4-(trifluoromethyl)benzamido)propanoic acid

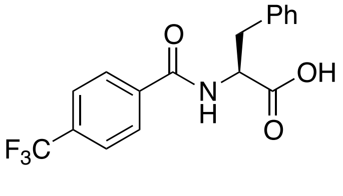

To a pure H_2O (250 mL) solution of NaOH (1800 mg, 45 mmol) was added (*L*)-phenylalanine (2477.9 mg, 15 mmol), and the mixture was stirred at 0 °C for 15 min under N_2. To the reaction mixture was added 4-(trifluoromethyl)benzoyl chloride (2.23 mL, 15 mmol) dropwise at same temperature, and it was stirred at room temperature for 2.5 h. The mixture was quenched by adding 1.0 N aqueous solution of HCl (100 mL, 100 mmol) at 0 °C, and white precipitate was appeared. The precipitate was washed with H_2O to afford the wet white solid. The slurry was dissolved into CH_2Cl_2 (100 mL), washed with H_2O (200 mL), and extracted with CH_2Cl_2 (100 mL×3). The organic layer was dried over Na_2SO_4 and filtrated. Evaporation of the filtrate gave white solid (3.93 g). However 4-(trifluoromethyl)benzoic acid was included as impurity (*ca.* 22% impurity: based on the molar ratio). So desired product was obtained in ca. 67% yield (*ca.* 10.1 mmol, 78% purity). This compound was used for following reactions without further purification.

(*S*)-*N*-(1-(octylamino)-1-oxo-3-phenylpropan-2-yl)-4-(trifluoromethyl)benzamide (3s)

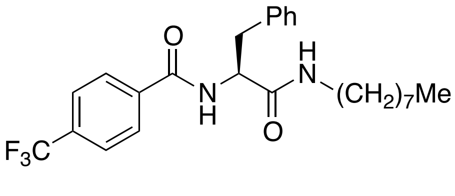

To an anhydrous THF (75 mL) solution of (*S*)-3-phenyl-2-(4-(trifluoromethyl)benzamido)propanoic acid (78% purity) (5.06 g, 13.0 mmol) were added 1-(3-Dimethylaminopropyl)-3-ethylcarbodiimide hydrochloride

(EDCI) (6.90 mg, 36 mmol), 1,2,3-benzotriazol-1-ol monohydrate (HOBt·H_2O) (2.71 mg, 17.7 mmol) and *N*-methylmorpholine (7.92 mL, 72 mmol). The mixture was stirred at 0 °C for 30 min under N_2. To the reaction mixture was added octylamine (12.43 mL, 75 mmol) dropwise at same temperature, and was stirred at room temperature overnight. The reaction mixture was quenched by adding a small portion of water (*ca.* <2 mL) at 0 °C, and the organic phase was removed *in vacuo* (*ca.* 1 mmHg, room temperature). The residue was dissolved into CH_2Cl_2 (300 mL), washed with HCl (aq) (1N, 200 mL×2), $NaHCO_3$ (aq) (saturated, 200 mL×2), brine (aq) (saturated, 100 mL×1), and extracted with CH_2Cl_2 (300 mL×2). The organic layer was dried over Na_2SO_4 and filtrated. Evaporation of the filtrate gave colorless solid, which was recrystallized from CH_2Cl_2 giving **3s** (4.31 g, 9.61 mmol, 64%) as colorless solid. IR (neat): 3306, 2925, 2854, 1637, 1538, 1327, 1171, 1131 cm^{-1}. ^1H NMR ($CDCl_3$, 600 MHz): δ 0.87 (t, J = 6.90 Hz, 3H), 1.12–1.40 (m, 12H), 3.03–3.14 (m, 2H), 3.17–3.26 (m, 2H), 4.80 (q, J = 6.18 Hz, 1H), 5.83 (d, J = 4.80 Hz, 1H), 7.21–7.30 (m, 5H), 7.33 (dd, J = 6.90 Hz, J = 2.10 Hz, 1H), 7.67 (d, J = 8.28 Hz, 2H), 7.87 (d, J = 8.28 Hz, 2H). ^{13}C NMR ($CDCl_3$, 150 MHz): δ 14.0, 22.6, 26.8, 29.12, 29.18, 29.23, 31.7, 38.8, 39.7, 55.4, 123.6 (q, $^1J_{CF}$ = 270 Hz), 125.48, 125.51, 127.0, 127.7 (2C), 128.6 (2C), 129.3 (2C), 133.4 (q, $^2J_{CF}$ = 31.6 Hz), 136.7, 137.0, 165.9, 170.7. HRMS (FAB, MH^+) calcd for $C_{25}H_{32}F_3N_2O_2^+$: 449.2410; Found: m/z = 449.2400. (Supplementary Figs. S46–S49)

(*S*)-*tert*-butyl (4-methyl-1-(octylamino)-1-oxopentan-2-yl)carbamate

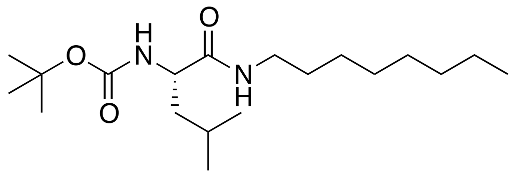

To an anhydrous THF (100 mL) solution of Boc–(*L*)-leucine–OH · H_2O (3739.7 mg, 15 mmol) were added 1-(3-Dimethylaminopropyl)-3-ethylcarbodiimide hydrochloride (EDCI) (9968.4 mg, 52 mmol), 1,2,3-benzotriazol-1-ol monohydrate (HOBt·H_2O) (2710.6 mg, 17.7 mmol) and *N*-methylmorpholine (7.92 mL, 72 mmol). The mixture was stirred at 0 °C for 30 min under N_2. To the reaction mixture was added octylamine (12.43 mL, 75 mmol) dropwise at same temperature, and was stirred at room

temperature overnight. The reaction mixture was quenched by adding a small portion of water (*ca.* <2 mL) at 0 °C, and the organic phase was removed *in vacuo* (*ca.* 1 mmHg, room temperature). The residue was dissolved into Et_2O (200 mL), washed with citric acid (aq) (5%, 50 mL×2), $NaHCO_3$ (aq) (saturated, 50 mL×1), brine (aq) (saturated, 50 mL×1), and extracted with CH_2Cl_2 (100 mL×3). The organic layer was dried over Na_2SO_4 and filtrated. Evaporation of the filtrate gave as a colorless solid (3.33 g, 10.15 mmol, 67%). This compound was used for following reactions without further purification.

(*S*)-4-methyl-2-(4-(trifluoromethyl)benzamido)pentanoic acid

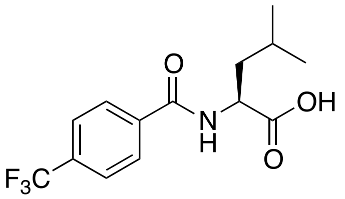

To a pure H_2O (500 mL) solution of NaOH (3600 mg, 90 mmol) were added (*L*)-leucine (3935.1 mg, 30 mmol), and the mixture was stirred at 0 °C for 15 min under N_2. To the reaction mixture was added 4-(trifluoromethyl)benzoyl chloride (4456.4 μL, 30 mmol) dropwise at same temperature, and it was stirred at room temperature for 2.5 h. The mixture was quenched by adding 1.0 N aqueous solution of HCl (200 mL, 200 mmol) at 0 °C, and white precipitate was appeared. The precipitate was washed with H_2O to afford the wet white solid. The slurry was dissolved into CH_2Cl_2 (200 mL), washed with H_2O (400 mL), and extracted with CH_2Cl_2 (100 mL×3). The organic layer was dried over Na_2SO_4 and filtrated. Evaporation of the filtrate gave white solid (9.07 g). However 4-(trifluoromethyl)benzoic acid was included as impurity (*ca.* 29% impurity: based on the molar ratio). So desired product was obtained in ca. 79% yield (*ca.* 23.8 mmol, 71% purity). This compound was used for following reactions without further purification.

N-((S)-4-methyl-1-(((S)-4-methyl-1-(octylamino)-1-oxopentan-2-yl)amino)-1-oxopentan-2-yl)-4-(trifluoromethyl)benzamide (3t)

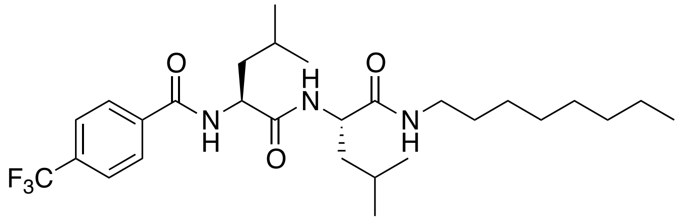

To an anhydrous CH_2Cl_2 (7.0 mL) solution of (*S*)-*tert*-butyl (4-methyl-1-(octylamino)-1-oxopentan-2-yl)carbamate (2397.6 mg, 7.0 mmol) was added trifluoroacetic acid (TFA) (7.0 mL, 91.4 mmol). The mixture was stirred at room temperature for 1 h under N_2, and the organic phase was removed *in vacuo* (*ca.* 1 mmHg, room temperature). To the residue were added THF (50 mL), 1-(3-Dimethylaminopropyl)-3-ethylcarbodiimide hydrochloride (EDCI) (3450.6 mg, 18 mmol), 1,2,3-benzotriazol-1-ol monohydrate (HOBt･H_2O) (1531.4 mg, 10 mmol), *N*-methylmorpholine (4.40 mL, 40 mmol) and (*S*)-4-methyl-2-(4-(trifluoromethyl)benzamido)pentanoic acid (71% purity) (2122.9 mg, 5.6 mmol) at 0 °C, and stirred at room temperature overnight. The reaction mixture was quenched by adding a small portion of water (*ca.* <2 mL) at 0 °C, and the organic phase was removed *in vacuo* (*ca.* 1 mmHg, room temperature). The residue was dissolved into CH_2Cl_2 (100 mL), washed with HCl (aq) (1 N, 100 mL×2), $NaHCO_3$ (aq) (saturated, 50 mL×1), brine (aq) (saturated, 50 mL×1), and extracted with CH_2Cl_2 (100 mL×3). The organic layer was dried over Na_2SO_4 and filtrated. The evaporation of the filtrate gave a yellow crude oil, which was purified by column chromatography on silica gel (hexane/EtOAc = 4/1 and only EtOAc) to afford the target compound (**3t**) (2099.8 mg, 3.98 mmol, 57%) as white solid. IR (neat): 3289, 3078, 2957, 2930, 2858, 1639, 1550, 1328, 1170, 1134, 1067 cm^{-1}. ^1H NMR (600 MHz, $CDCl_3$): δ 0.81–0.96 (m, 15H), 1.17–1.32 (m, 10H), 1.37–1.80 (m, 8H), 3.10 (m, 0.4H, J = 7.6 Hz), 3.16 (m, 1H, J = 5.5 Hz), 3.26 (m, 0.6H, J = 7.6 Hz), 4.49 (q, 0.4H, J = 5.5 Hz), 4.54 (q, 0.6H, J = 8.3 Hz), 4.72 (q, 0.4H, J = 6.9 Hz), 4.87 (q, 0.6H, J = 8.3 Hz), 6.69 (t, 0.4H, J = 5.5 Hz), 6.76 (t, 0.6H, J = 11 Hz), 7.13 (d, 0.4H, J = 8.3 Hz), 7.43 (d, 0.6H, J = 8.28 Hz), 7.48 (t, 1H, J = 8.9 Hz), 7.63 (t, 2H, J = 8.9 Hz), 7.92 (t, 2H, J = 8.9 Hz). ^{13}C NMR ($CDCl_3$,

150 MHz): δ 14.0, 22.1 (0.4C), 22.4, 22.52 (2C), 22.55 (2C), 22.8 (0.6C), 24.87 (0.6C), 24.92, 24.96 (0.4C), 26.9 (0.4C), 27.0 (0.6C), 29.15 (0.4C), 29.18 (0.6C), 29.24 (0.4C), 29.28 (0.6C), 29.34 (0.4C), 29.38 (0.6C), 31.7 (0.4C), 31.8 (0.6C), 39.6 (0.6C), 39.7 (0.4C), 41.4 (0.6C), 41.5, 41.8 (0.4C), 51.9 (0.6C), 52.0 (0.6C), 52.1 (0.4C), 52.8 (0.4C), 123.7 (q, $^{1}J_{CF}$ = 264 Hz), 125.1 (1.2C), 125.2 (0.8C), 128.0 (0.8C), 128.1 (1.2C), 133.1 (q, $^{2}J_{CF}$ = 34.5 Hz), 137.0 (0.4C), 137.5 (0.6C), 166.0 (0.6C), 166.2 (0.4C), 171.6 (0.6C), 172.1, 172.5 (0.4C). HRMS (FAB, MH^+) calcd for $C_{28}H_{45}F_3N_3O_3^+$: 528.3408; Found: *m*/*z* = 528.3385. (Supplementary Figs. S50–S53)

***N*-(quinolin-8-yl)nonanamide (3u)**

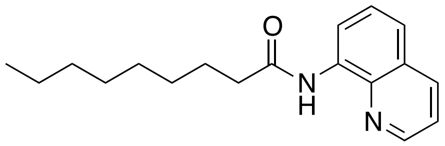

To an anhydrous CH_2Cl_2 (20 mL) solution of nonanoyl chloride (2.70 mL, 15 mmol) were added Et_3N (2.80 mL, 20 mmol) and 8-aminoquinoine (2.88 mL, 20 mmol). The mixture was stirred at room temperature overnight under Ar, and was quenched by adding a small portion of water (*ca.* <2 mL). The residue was dissolved into CH_2Cl_2 (100 mL), washed with HCl (aq) (1M, 100 mL×1), $NaHCO_3$ (aq) (saturated, 100 mL×1), brine (aq) (saturated, 100 mL×1), and extracted with CH_2Cl_2 (100 mL×2). The organic layer was dried over Na_2SO_4 and filtrated. Evaporation of the filtrate gave dark brown oil, which was distilled by Kugel-Rohr (230 °C/0.006 mmHg) to give **3u** as pale yellow oil (3.43 g, 12.04 mmol, 80%). IR (neat): 3357, 3048, 2925, 2854, 1686, 1523, 1485, 1325, 1163, 1106 cm^{-1}. ^{1}H NMR ($CDCl_3$, 600 MHz): δ 0.87 (t, J = 6.84 Hz, 3H), 1.21–1.40 (m, 8H), 1.43 (quin, J = 7.56 Hz, 2H), 1.82 (quin, J = 7.56 Hz, 2H), 2.56 (t, J = 7.56 Hz, 2H), 7.45 (dd, J = 8.25 Hz, J = 4.14 Hz, 1H), 7.49 (dd, J = 8.28 Hz, J = 1.38 Hz, 1H), 7.54 (t, J = 8.22 Hz, 1H), 8.16 (dd, J = 8.28 Hz, J = 2.04 Hz, 1H), 8.77–8.82 (m, 2H), 9.81 (s, 1H). ^{13}C NMR ($CDCl_3$, 150 MHz): δ 14.1, 22.6, 25.7, 29.2, 29.3, 29.4, 31.8, 38.3, 116.4, 121.3, 121.5, 127.5, 127.9, 134.6, 136.3, 138.4, 148.1, 171.9. HRMS (FAB, MH^+) calcd for $C_{18}H_{25}N_2O^+$: 285.1961; Found: *m*/*z* = 285.1952. (Supplementary Figs. S54–S57)

1-(2,3-dihydro-1*H*-pyrrolo[2,3-*b*]pyridin-1-yl)nonan-1-one (3v)

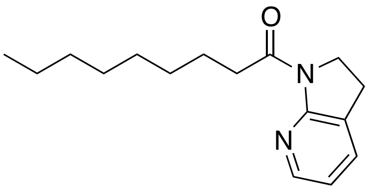

To an anhydrous THF (20 mL) solution of nonanoyl chloride (1.08 mL, 6 mmol) were added K_2CO_3 (1382 mg, 10 mmol) and 2,3-dihydro-7-azaindole (600 mg, 5 mmol). The mixture was stirred at room temperature overnight under Ar, and was quenched by adding a small portion of water (*ca.* <5 mL). The residue was dissolved into CH_2Cl_2 (50 mL), washed with $NaHCO_3$ (aq) (saturated, 100 mL), brine (aq) (saturated, 100 mL), and extracted with CH_2Cl_2 (50 mL×3). The organic layer was dried over Na_2SO_4 and filtrated. Evaporation of the filtrate gave yellow oil, which was distilled by Kugel-Rohr (180 °C/0.006 mmHg) to give **3v** as colorless oil (697 mg, 2.68 mmol, 54%). IR (neat): 3509, 3303, 3053, 2925, 2854, 1652, 1587, 1417, 1243 cm^{-1}. ^1H NMR ($CDCl_3$, 600 MHz): δ 0.88 (t, J = 6.84 Hz, 3H), 1.23–1.37 (m, 8H), 1.41 (quin, J = 7.56 Hz, 2H), 1.71 (quin, J = 7.56 Hz, 2H), 3.04 (t, J = 8.28 Hz, 2H), 3.13 (t, J = 7.56 Hz, 2H), 4.10 (t, J = 8.22 Hz, 2H), 6.85 (dd, J = 7.56 Hz, J = 4.80 Hz, 1H), 7.44 (dd, J = 7.56 Hz, J = 1.38 Hz, 1H), 8.11 (d, J = 3.42 Hz, 1H). ^{13}C NMR ($CDCl_3$, 150 MHz): δ 14.1, 22.6, 24.2, 25.0, 29.2, 29.4 (2C), 31.8, 36.5, 45.5, 117.7, 126.0, 133.2, 146.1, 156.1, 173.4. HRMS (FAB, MH^+) calcd for $C_{16}H_{25}N_2O^+$: 261.1961; Found: m/z = 261.1968. (Supplementary Figs. S58–S61)

1,2,3,4-tetrahydroquinolin-8-amine (5u)[13]

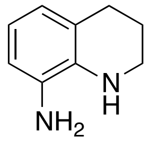

Compound **5u** is rather easily oxidized by air.[13] ^1H NMR ($CDCl_3$, 600 MHz): δ 1.91 (quin, J = 5.52 Hz, 2H), 2.76 (t, J = 6.18 Hz, 2H), 3.1–3.3 (br, 3H), 3.32 (t, J = 4.86 Hz, 2H), 6.52–6.60 (m, 3H). ^{13}C NMR ($CDCl_3$, 150 MHz): δ 22.4, 27.0, 42.5, 114.0, 118.0,

121.1, 123.2, 133.8, 133.9. HRMS (ESI, MH^+) calcd for $C_9H_{13}N_2^+$: 149.1073; Found: *m*/*z* = 149.1067. (Supplementary Figs. S62–S64)

Representative procedure for hydrogenation of *N*-benzylbenzamide (3a) (Fig. 2a): The reaction with RUPIP2 (1a) (Fig. 2a, entry 2)

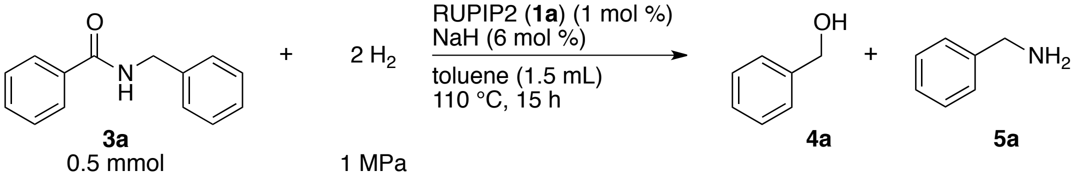

Under a continuous Ar flow, RUPIP2 (**1a**) (2.94 mg, 0.005 mmol), sodium hydride (55% oil dispersion, 1.31 mg, 0.03 mmol), anhydrous toluene (1.5 mL), *N*-benzylbenzamide (**3a**) (105.6 mg, 0.5 mmol) and a magnetic stirring bar were placed in a dried Teflon tube (21 mL capacity). The Teflon tube was quickly inserted into an autoclave, and the inside of the autoclave was purged 10 times with hydrogen gas (1 MPa). The autoclave was pressurized with a 1 MPa of hydrogen gas at 25 °C, and heated at 110 °C for 24 h under stirring (800 rpm). The autoclave was cooled to room temperature in an ice–water (0 °C) bath, and the reaction mixture was quenched with a 2.0 M Et_2O solution of HCl (15 μL, 0.03 mmol). The organic phase was removed *in vacuo* (*ca.* 100 mmHg, 40 °C). The residue was diluted with $CDCl_3$, and analyzed by ^1H NMR. The yields of benzyl alcohol (**4a**) (81%) and benzylamine (**5a**) (81%) were calculated based on the integral ratio among the signals of these compounds with respected to an internal standard (1,1,2,2-tetrachloroethane).

ESI-MS analysis of preactivated catalyst (Fig. 2b)

With *N*-benzylbenzamide (3a)

Under a continuous Ar flow, RUPIP2 (**1a**) (3.98 mg, 0.0067 mmol), sodium hydride (55% oil dispersion, 2.91 mg, 0.067 mmol), *N*-benzylbenzamide (**3a**) (2.11 mg, 0.01 mmol), anhydrous toluene (2.0 mL) and a magnetic stirring bar were placed in a dried Teflon tube (21 mL capacity). The Teflon tube was quickly inserted into an autoclave, and the inside of the autoclave was purged 10 times with hydrogen gas (1 MPa). The autoclave was pressurized with a 1 MPa of hydrogen gas at 25 °C, and heated at 110 °C

for 5 h under stirring (800 rpm). Then the autoclave was cooled to room temperature in an ice–water (0 °C) bath, and hydrogen was blown away under an Ar stream. 10 μL of reaction mixture was diluted with 3.35 mL of MeCN. Then this solution was used for ESI analysis (Supplementary Fig. S1).

Without *N*-benzylbenzamide (3a)

Under a continuous Ar flow, RUPIP2 (**1a**) (3.98 mg, 0.0067 mmol), sodium hydride (55% oil dispersion, 2.91 mg, 0.067 mmol), anhydrous toluene (2.0 mL) and a magnetic stirring bar were placed in a dried Teflon tube (21 mL capacity). The Teflon tube was quickly inserted into an autoclave, and the inside of the autoclave was purged 10 times with hydrogen gas (1 MPa). The autoclave was pressurized with a 1 MPa of hydrogen gas at 25 °C, and heated at 110 °C for 5 h under stirring (800 rpm). Then the autoclave was cooled to room temperature in an ice–water (0 °C) bath, and hydrogen was blown away under an Ar stream. 10 μL of reaction mixture was diluted with 3.35 mL of MeCN. Then this solution was used for ESI analysis (Supplementary Fig. S2).

Representative procedure for hydrogenation of amides (3) with non-preactivated catalyst (conditions A) (Fig. 2a, Fig. 3, Fig 4., and Fig. 5b): The reaction of *N*-benzylbenzamide (3a).

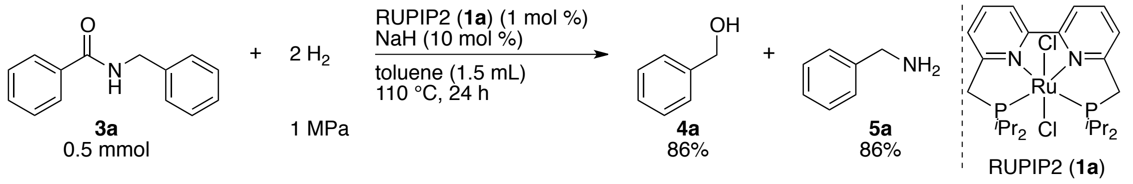

Under a continuous Ar flow, RUPIP2 (**1a**) (2.94 mg, 0.005 mmol), sodium hydride (55% oil dispersion, 2.18 mg, 0.05 mmol), anhydrous toluene (1.5 mL), *N*-benzylbenzamide (**3a**) (105.6 mg, 0.5 mmol) and a magnetic stirring bar were placed in a dried Teflon tube (21 mL capacity). The Teflon tube was quickly inserted into an autoclave, and the inside of the autoclave was purged 10 times with hydrogen gas (1 MPa). The autoclave was pressurized with a 1 MPa of hydrogen gas at 25 °C, and heated at 110 °C for 24 h under stirring (800 rpm). The autoclave was cooled to room temperature in an ice–water (0 °C) bath, and the reaction mixture was quenched with

2.0 M Et_2O solution of HCl (25 μL, 0.05 mmol). The organic phase was removed *in vacuo* (*ca.* 100 mmHg, 40 °C). The residue was diluted with $CDCl_3$, and analyzed by ^1H NMR. The yields of benzyl alcohol (**4a**) (86%) and benzylamine (**5a**) (86%) were calculated based on the integral ratio among the signals of these compounds with respected to an internal standard (mesitylene).

Afterward, the reaction mixture was diluted with CH_2Cl_2, and HCl (2 M in Et_2O, 250 μL, 0.5 mmol) was added dropwise. White precipitate was generated and purified by filtration to give benzylamine hydrochloride (52.1 mg, 0.364 mmol, 73%). Then the filtrate was purified by column chromatography on silica gel (eluent; Et_2O/hexane = 2/3) to give *N*-benzylbenzamide (9.5 mg, 0.045 mmol, 9%) and benzyl alcohol (44.7 mg, 0.413 mmol, 83%) (Supplementary Table S1).

Representative procedure for hydrogenation of amides 3 with preactivated catalyst (conditions B) (Fig. 3a, Fig. 4a, and Figs. 5a and 5c): The reaction of *N,N'*-dimethylbenzamide (3j).

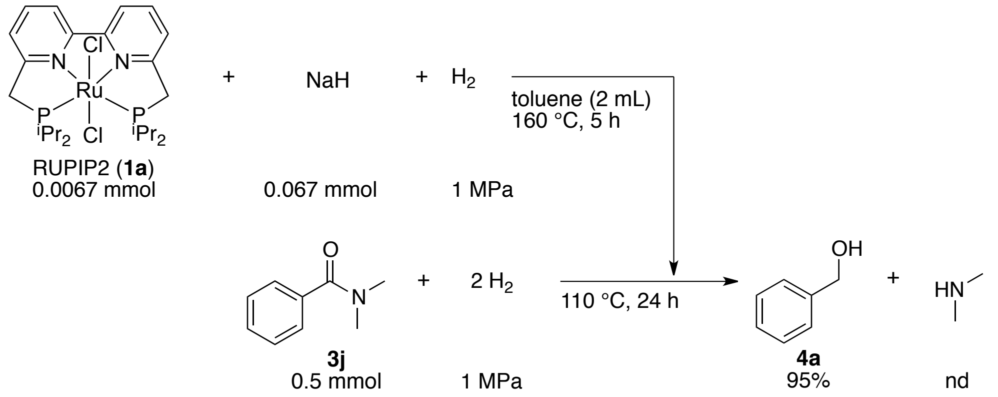

Under a continuous Ar flow, RUPIP2 (**1a**) (3.98 mg, 0.0067 mmol), sodium hydride (55% oil dispersion, 2.91 mg, 0.067 mmol), anhydrous toluene (2.0 mL) and a magnetic stirring bar were placed in a dried Teflon tube (21 mL capacity). The Teflon tube was quickly inserted into an autoclave, and the inside of the autoclave was purged 10 times with hydrogen gas (1 MPa). The autoclave was pressurized with a 1 MPa of hydrogen gas at 25 °C, and heated at 160 °C for 5 h under stirring (800 rpm). Then the autoclave was cooled to room temperature in an ice–water (0 °C) bath, and hydrogen was blown away under an Ar stream. Under the continuous Ar flow, to another autoclave, in which

a dried Teflon tube charged with *N,N'*-dimethylbenzamide (**3j**) (74.6 mg, 0.5 mmol) had been in advance inserted, was transferred the reaction mixture (1.5 mL, 1 mol % Ru) using a gas-tight syringe (2.5 mL). The autoclave was purged (×10 with 1 MPa H_2 gas) and finally charged with 1 MPa of hydrogen gas, and the reaction mixture was heated at 110 °C for 24 h under stirring (800 rpm). The autoclave was cooled to room temperature in an ice–water (0 °C) bath, and the reaction mixture was quenched with 2.0 M Et_2O solution of HCl (25 μL, 0.05 mmol). The organic phase was removed *in vacuo* (*ca.* 100 mmHg, 40 °C). The residue was diluted with $CDCl_3$, and analyzed by ^1H NMR. The yield of benzyl alcohol (**4a**) (95%) was calculated based on the integral ratio among the signals of these compounds with respect to an internal standard (mesitylene) (Supplementary Table S1).

Procedure for hydrogenation of benzanilide (3w) with *β*-citronellol and *trans*-5-Decen-1-ol (Fig. 5d).

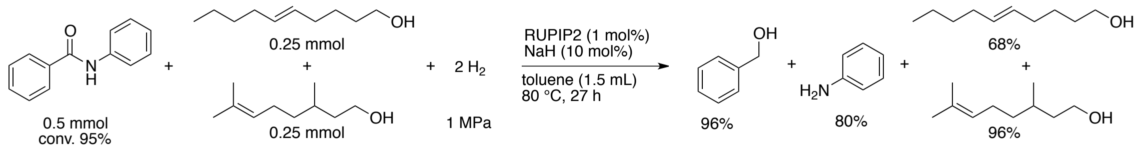

Under a continuous Ar flow, RUPIP2 (**1a**) (2.94 mg, 0.005 mmol), sodium hydride (55% oil dispersion, 2.18 mg, 0.05 mmol), anhydrous toluene (1.5 mL), benzanilide (3w) (98.6 mg, 0.5 mmol), *β*-citronellol (45.6 μL, 0.25 mmol), *trans*-5-Decen-1-ol (39.07 mg, 0.25 mmol) and a magnetic stirring bar were placed in a dried Teflon tube (21 mL capacity). The Teflon tube was quickly inserted into an autoclave, and the inside of the autoclave was purged 10 times with hydrogen gas (1 MPa). The autoclave was pressurized with a 1 MPa of hydrogen gas at 25 °C, and heated at 80 °C for 27 h under stirring (800 rpm). The autoclave was cooled to room temperature in an ice–water (0 °C) bath, and the reaction mixture was quenched with 2.0 M Et_2O solution of HCl (25 μL, 0.05 mmol). The organic phase was removed *in vacuo* (*ca.* 100 mmHg, 40 °C). The residue was diluted with $CDCl_3$, and analyzed by ^1H NMR. The yields of benzyl alcohol (**4a**) (96%), aniline (80%), *β*-citronellol (96%) and *trans*-5-Decen-1-ol (68%) were calculated based on the integral ratio among the signals of these compounds with respected to an internal standard (mesitylene).

Representative procedure for hydrogenation of cyclic amides with RuPIP2 (1a), sodium tetraphenylborate and L-Selectride: The reaction of ε-caprolactam (3h) (Fig. 3b)

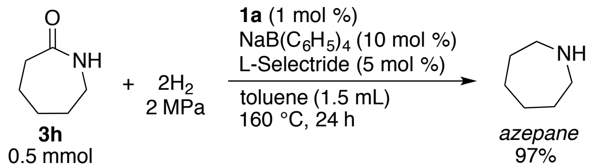

RuPIP2 (**1a**) (2.9 mg, 0.005 mmol), sodium tetraphenylborate (17.1 mg, 0.05 mmol), ε-caprolactam (**3h**) (56.6 mg, 0.5 mmol) and a magnetic stirring bar were placed in a glass tube (21 mL capacity). The glass tube was inserted into an autoclave, and anhydrous toluene (1.5 mL) and L-Selectride® solution (1 M in THF, 25 μL, 0.025 mmol) were added to the mixture under argon atmosphere. The autoclave was purged several times with hydrogen gas (1 MPa). The autoclave was pressurized with 2 MPa of hydrogen gas at 25 °C, and heated for 24 hours at 160 °C under stirring (1000 rpm). The autoclave was cooled to room temperature in an ice–water (0 °C) bath. The reaction mixture was diluted with $CDCl_3$ and analyzed by ^1H NMR. The yield of azepane (97%) was calculated based on the integral ratio among the signals of these compounds with respected to an internal standard (mesitylene).

X-ray single crystal structure analysis of RUPCY2 (1b).

Single crystals of RUPCY2 suitable for X-ray crystal analysis were obtained by slow diffusion of hexane into a chloroform solution of RUPCY2. Intensity data were collected at 103 K on a Rigaku Single Crystal CCD X-ray Diffractometer (Saturn 70 with MicroMax-007) with Mo Kα radiation (λ = 0.71075 Å) and graphite monochromater. A total of 21375 reflections were measured at a maximum 2θ angle of 49.96°, of which 6042 were independent reflections (R_{int} = 0.1018). The structure was solved by direct methods (SHELXS-97) and refined by the full-matrix least-squares on F_2 (SHELXL-97). All non-hydrogen atoms were refined anisotropically. All hydrogen atoms were placed using AFIX instructions. The following crystal structure has been deposited at the Cambridge Crystallographic Data Centre and allocated the deposition number CCDC1022649.

The crystal data are as follows: $C_{36}H_{54}Cl_2N_2P_2Ru$; FW = 748.72, crystal size 0.20 × 0.20 × 0.20 mm^3, monoclinic, C2/c, a = 34.191(16) Å, b = 13.813(6) Å, c = 14.856(6) Å,

α = 90.00°, β = 93.164(9)°, γ = 90.00°, V = 7006(5) Å^3, Z = 8, D_c = 1.420 g cm^{-3}. The refinement converged to R_1 = 0.0885, wR_2 = 0.2118 ($I > 2\sigma(I)$), GOF = 1.081 (Supplementary Fig. S11).

Supplementary References

(1) Prosser, A. R., Banning, J. E., Rubina, M. & Rubin, M. *Org. Lett.* **12**, 3968–3971 (2010).

(2) Agwada, V. C. *J. Chem. Eng. Data* **27**, 479–481 (1982).

(3) Huang, Z., Reilly, J. E. & Buckle, R. N. *Synlett*, **7**, 1026–1030 (2007).

(4) Miura, T., Held, I., Oishi, S., Naruto, M. & Saito, S. *Tetrahedron Lett.* **54**, 2674–2678 (2013).

(5) Martinelli, J. R., Clark. T. P., Watson, D. A., Munday, R. H. & Buchwald, S. L. *Angew. Chem. Int. Ed.* **46**, 8460–8463 (2007).

(6) Iranp oor, N., Firouzabadai, H. & Khalili, D. *Bull. Chem. Soc. Jpn.* **83**, 923–934 (2010).

(7) Zhang, L., Su, S., Wu, H. & Wang, S. *Tetrahedron* **65**, 10022–10024 (2009) .

(8) Zou, B., Dreger, K., Lichtenfeld, C. M., Grimme, S., Schäfer, H. J., Fuchs, H. & Chi, L. *Langmuir* **21**, 1364–1370 (2005).

(9) Pasha, M.A. & Jayashankara, V. P. *Synthetic Communications* **36**, 1787–1793 (2006).

(10) Kung, C.-H. & Kwon, C.-H. *Med. Chem. Res.* **19**, 498–513 (2010).

(11) Li, W., Xie, J.-H., Yuan M.-L. & Zhou, Q. L. *Green Chem.* **16**, 4081–4085 (2014).

(12) Langer, R., Fuchs, I., Vogt, I., Balaraman, E., Diskin-Posner, Y., Shimon, L. J. W., Ben-David, Y. & Milstein, D. *Chem. Eur. J.* **19**, 3407 – 3414 (2013).

(13) Benincori, T., Pagani, S. B., Fusco, R. & Sannicolo, F. J. *Chem. Soc. Perkin Trans.* 2721–2728 (1988).
